# Innovative dual-gene delivery platform using miR-124 and PD-1 via umbilical cord mesenchymal stem cells and exosome for glioblastoma therapy

**DOI:** 10.1186/s13046-025-03336-4

**Published:** 2025-03-25

**Authors:** Po-Fu Yueh, I-Tsang Chiang, Yueh-Shan Weng, Yu-Chang Liu, Raymond C. B. Wong, Cheng-Yu Chen, Justin Bo-Kai Hsu, Long-Bin Jeng, Woei-Cherng Shyu, Fei-Ting Hsu

**Affiliations:** 1 Institute of Traditional Medicine, School of Medicine, National Yang Ming Chiao Tung University, 112 Taipei, Beitou, Taiwan (ROC); 2 Research assistant Center, Show Chwan Memorial Hospital, 500 Changhua, Taiwan (ROC); 3 Department of Medical Imaging and Radiological Sciences, Central Taiwan University of Science and Technology, 406 Taichung, Taiwan (ROC); 4 Department of Radiation Oncology, Chang Bing Show Chwan Memorial Hospital, 505 Lukang, Changhua, Taiwan (ROC); 5 Department of Biological Science and Technology, China Medical University, 406 Taichung, Taiwan (ROC); 6 Centre for Eye Research Australia, Royal Victorian Eye and Ear Hospital, 3002 East Melbourne, VIC Australia; 7 Ophthalmology, Department of Surgery, University of Melbourne, 3002 East Melbourne, VIC Australia; 8 Department of Radiology, School of Medicine, College of Medicine, Taipei Medical University, Taipei 110, Taipei, Taiwan (ROC); 9 Department of Medical Imaging, Taipei Medical University Hospital, 112 Taipei, Taiwan (ROC); 10 Department of Computer Science and Engineering, Yuan Ze University, 320 Taoyuan, Taiwan (ROC); 11 Organ Transplantation Center, China Medical University Hospital, 404 Taichung, Taiwan (ROC); 12 Cell Therapy Center, China Medical University Hospital, 404 Taichung, Taiwan (ROC); 13 School of Medicine, China Medical University, 404 Taichung, Taiwan (ROC); 14 Graduate Institute of Biomedical Sciences, China Medical University, 404 Taichung, Taiwan (ROC); 15 Neuroscience and Brain Disease Center, China Medical University, 404 Taichung, Taiwan (ROC); 16 Translational Medicine Research Center, Drug Development Center, Department of Neurology, China Medical University & Hospital, 404 Taichung, Taiwan (ROC); 17 Department of Life Sciences, National Central University, 320 Taoyuan, Taiwan (ROC); 18 LOHAS Naturopathic Medical Center, Chang Bing Show Chwan Memorial Hospital, Lukang, Taiwan (ROC); 19 Department of Radiation Oncology, Show Chwan Memorial Hospital, Changhua, Taiwan (ROC); 20 Evidence-Based Medicine Center, Chang Bing Show Chwan Memorial Hospital, Lukang, Taiwan (ROC); 21 Center of Quality Management, Chang Bing Show Chwan Memorial Hospital, Lukang, Taiwan (ROC)

**Keywords:** Glioblastoma, Gene therapy, miR-124, Umbilical cord mesenchymal stem cells, CDK4/6

## Abstract

Addressing the challenges of identifying suitable targets and effective delivery strategies is critical in pursuing therapeutic solutions for glioblastoma (GBM). This study focuses on the therapeutic potential of microRNA-124 (miR-124), known for its tumor-suppressing properties, by investigating its ability to target key oncogenic pathways in GBM. The results reveal that CDK4 and CDK6—cyclin-dependent kinases that promote cell cycle progression—are significantly overexpressed in GBM brain samples, underscoring their role in tumor proliferation and identifying them as critical targets for miR-124 intervention. However, delivering miRNA-based therapies remains a major obstacle due to the instability of RNA molecules and the difficulty in achieving targeted, efficient delivery. To address these issues, this research introduces an innovative, non-viral dual-gene delivery platform that utilizes umbilical cord mesenchymal stem cells (UMSCs) and their exosomes to transport miR-124 and programmed cell death protein-1 (PD-1). The efficacy of this dual-gene delivery system was validated using an orthotopic GBM model, which closely mimics the tumor microenvironment seen in patients. Experimental results demonstrate that the UMSC/*miR-124-PD-1* complex and its exosomes successfully induce apoptosis in GBM cells, significantly inhibiting tumor growth. Notably, these treatments show minimal cytotoxic effects on normal glial cells, highlighting their safety and selectivity. Moreover, the study highlights the immunomodulatory properties of UMSC/*miR-124-PD-1* and its exosomes, enhancing the activation of immune cells such as T cells and dendritic cells, while reducing immunosuppressive cells populations like regulatory T cells and myeloid-derived suppressor cells. The orchestrated dual-gene delivery system by UMSCs and exosomes showcased targeted tumor inhibition and positive immune modulation, emphasizing its potential as a promising therapeutic approach for GBM.

**Supplementary Information:**

The online version contains supplementary material available at 10.1186/s13046-025-03336-4.

## Introduction

Glioblastoma (GBM) stands as the most prevalent and highly aggressive malignant brain tumor affecting adults. Upon diagnosis, the standard treatment typically involves a multimodal approach that includes surgical resection, radiation therapy, and chemotherapy [1]. Due to the challenges posed by the blood-brain barrier (BBB), several innovative biomaterial-based strategies have been developed to address these limitations [2]. Biomaterials can also be utilized to deliver innovative treatments [3], including gene therapy, chemotherapy [4], photodynamic therapy [5], anti-angiogenic therapy [6], and thermotherapy [7]. However, nanoparticles that originate from non-biological sources often face issues such as organ clearance and accumulation, lack of self-targeting, poor degradation, and regulatory hurdles. As a result, surface functionalization becomes essential and unavoidable to enhance their biocompatibility and therapeutic efficacy [8, 9]. Recent studies have explored using mesenchymal stem cells (MSCs) as living carriers for the active delivery of therapeutic agents to tumor sites, with the advantage of potentially avoiding rejection due to their inherent immunosuppressive characteristics [10, 11]. Moreover, evidence suggests that umbilical cord-derived MSCs (UMSCs) offer significant advantages as transplantable cells compared to adult-derived MSCs from bone marrow or adipose tissue, making them promising candidates for clinical applications in treating various diseases [12]. Meanwhile, exosomes derived from gene-modified mesenchymal stem cells (MSCs) have emerged as a new delivery method, offering a lower potential for tumorigenesis [13].

Although numerous compounds targeting CTLA-4 and PD-L1 (programmed death-ligand 1) have been tested in clinical trials for GBM, none have significantly improved survival outcomes for newly diagnosed or recurrent cases in the past decade [14]. Gliomas are classified as ‘cold tumors,’ characterized by low immune cell infiltration, which makes immunotherapy ineffective as a standalone treatment [15]. Several reports suggest combining immune checkpoint inhibitors (ICIs) with traditional GBM treatments, such as radiation or chemotherapy, may enhance therapeutic efficacy [16]. These combination therapies could provide more potent, specific, and long-lasting anticancer immune responses compared to mono or sequential therapies [17]. Therefore, identifying an optimal combination strategy is crucial for maximizing the effectiveness of immune checkpoint inhibitors (ICIs) in GBM.

MicroRNAs (miRNAs) are small, non-coding RNA molecules that regulate gene expression by binding to messenger RNA (mRNA) and inhibiting protein translation. In cancer, miRNAs play a critical role as regulatory molecules, acting as either oncogenes or tumor suppressors [18]. Notably, miR-124 is a specific miRNA that is consistently downregulated in various types of cancer, and this downregulation is inversely correlated with tumor growth, lymph node metastasis, and poor prognosis [19]. In the case of GBM, miR-124 and miR-137 are significantly downregulated, and restoring their expression levels leads to increased cell cycle arrest in the G0/G1 phase [20]. Patients with miR-124 expression levels below the average typically show shorter survival times [21, 22]. Nanoparticle formulations have recently addressed delivery challenges and off-target issues associated with miRNA [23]. The use of MSCs and their exosome-based miRNA delivery has emerged as a disease-specific targeting method with biocompatible characteristics, especially when compared to other non-organic nanoparticles [24]. While large-scale isolation of exosomes remains a challenge, in this study, we developed a hypoxic condition for gene-modified UMSCs to achieve a greater yield [25].

To effectively deliver and target multiple sites within heterogeneous GBM, we designed umbilical cord-UMSCs as bio-based materials, incorporating the therapeutic PD-1 gene on the surface and miR-124 with G1 arrest capacity for GBM treatment. In addition, exosomes derived from this dual-target UMSCs (UMSC/*miR-124-PD-1*) were isolated to evaluate their anti-GBM capability.

## Materials and methods

### Glioblastoma samples

The human glioma specimens used in this study were obtained with approval from the Taipei Medical University Hospital Institutional Review Board, as per protocol IRB N201901041. We collected MRI scans with contrast enhancement and pathological reports diagnosing these specimens as grade 4 gliomas for subsequent RNA array testing (Welgene Biotech Co., Ltd., Human GE array V2). Human Brain Total RNA (Normal human brain (whole) pooled from 4 males Asians, age: 21–29, Clontech Laboratories, Inc. A Takara Bio Company, CA, USA, CATALOG No. 636530).

### Open sources analysis and platform

Sample from The Cancer Genome Atlas Program (TCGA) was utilized by GEPIA platform (Gene Expression Profiling Interactive Analysis, http://gepia.cancer-pku.cn/). The survival pattern of CDK4 and CDK6 expression was analyzed using a 50% cut-off value to differentiate between high and low expression levels. MicroRNA target annotation and correlation between specific genes was predicted by TargetScan (https://www.targetscan.org/).

### PiggyBac (PB) transposon miR-124/PD-1 plasmid construction

A PiggyBac vector, pPB-CMV-MCS-EF1α-RedPuro, containing the multiple cloning sites (MCS), PiggyBac terminal repeats (PB-TRs), core insulators, and a puromycin selection marker (BSD) fused with RFP driven by the human EF1α, was used as the base vector (System Bioscience). The bicistronic expression construct was developed by inserting the hsa-pre-miR-124 (human miR-124 microRNA precursor, Topgene and PD-1 (PDCD1 Human cDNA ORF, Origene) cDNA fragments into pPB-CMV-MCS-EF1α-RedPuro using a specific restriction enzyme linker (XbaI, NheI, SalI and EcoRI) to construct the pPB-(hsa-pre-miR-124)-PD1 plasmid bicistronic expression plasmid.

### Plasmid transfection of UMSCs

UMSCs were gently detached and harvested using Accutase (SCR, Merck, NJ, USA). Subsequently, a count of 5 × 10^5^ cells was performed, and the cells were pelleted at 200 × g for 5 minutes. The electroporation solution was prepared using the P1 Primary Cell 4D-Nucleofector™ X Kit L (Lonza, Basel, Switzerland). The electroporation mixture consisted of 82 µL of P1 Primary Cell Nucleofector^TM^ Solution and 18 µL of supplement, which were combined with 5 µg of PD-1, miR-124, miR-124/PD-1 and 5 µg of the transposase plasmid for each reaction. The UMSC pellet was carefully resuspended in this electroporation solution mixture and then transferred to the Nucleocuvette^TM^ vessel provided in the kit, ensuring the absence of any bubbles. The Nucleocuvette^TM^ vessel was subsequently placed in the Lonza 4D-Nucleofector (Lonza), and the cells were electroporated using program DO101. Following electroporation, the cells were transferred from the vessel into a pre-warmed T25 flask containing 5 mL of MSC media and 5% PLTGold. The media was refreshed the following day. To select positive cells, puromycin was introduced three days after electroporation at a concentration of 1 µg/mL. This concentration was increased to 2 µg/mL after one week of electroporation.

### Exosome isolation and validation

Exosomes were harvested from UMSC/*miR124-PD-1* supplements after the hypoxia condition for a 72-hour period. The culture supernatant underwent centrifugation at 3,000 × g for 30 min at 4 °C to eliminate dead cells and cell debris. Subsequently, the Total Exosome Isolation Reagent (4478359, Thermo Fisher Scientific) was employed for exosome isolation for western blotting, and a 0.22-µm PES membrane filter was used for diameter detection. Exosome quantification was performed with a BCA protein assay kit (Beyotime Biotechnology). Nanoparticle tracking analysis (NTA) was conducted to observe the size distribution and concentration of extracellular vehicles (EVs) utilizing a NanoSight NS300 system (Malvern Instruments). The analysis was executed with NTA 3.1 software, setting the threshold to 5 and using default parameters. The EVs were appropriately resuspended in PBS [26].

### Cells culture and reagents

GBM8401 and GL261 cells were separately obtained from the American Type Culture Collection (ATCC) and DSMZ (the Leibniz Institute DSMZ (Germany). The cells were cultured in a growth medium consisting of minimum essential medium (MEM) or Dulbecco’s modified Eagle’s medium (DMEM), supplemented with 10% fetal bovine serum (FBS), 1% sodium pyruvate, 2 mM L-glutamine, 100 units/mL penicillin, and 100 mg/mL streptomycin. They were maintained in a humidified incubator at 37 °C with a 5% CO_2_ atmosphere. The reagent used in this study was listed in Supplementary Table 1.

### Western blot assay

Total proteins from the cells were extracted with lysis buffer (50 mM Tris-hydrochloride [HCl, pH 8.0], 120 mM NaCl, 0.5% NP-40, and 1 mM phenylmethanesulfonyl fluoride. A cytosol extraction kit was used to extract the cytosolic cytochrome-c from the cells, following the manufacturer’s instructions. The expression levels of various proteins after treatment were determined using Western blotting. The proteins were visualized using the MultiGel-21 imaging system (TOP BIO CO., Taipei, Taiwan), and their band intensities were quantified using Image J (version 1.50, National Institutes of Health, Bethesda, MD, USA).

### Orthotopic glioblastoma model

Six-week-old male C57BL/6 mice was purchased from the National Laboratory Animal Center and housed in a pathogen-free animal facility. The animals were fed sterilised mouse chow and water. C57BL/6 mice (20–25 g) were used for intracerebral GL261 cell implantations. The animals were anaesthetised with 1–2% isofluorane. In brief, 10,000 glioblastoma cells in 3 µL Mg^2+^/Ca^2+^-free Hanks’ balanced salt solution (HBSS) was slowly (15–20 s) injected into the left-brain region at a depth of 3 mm from the dural surface of the mice. The detail procedure was described in previous studies [27, 28]. The mice were separated into various groups and administered once with different modulation form of UMSCs treatment (internal carotid artery injection): UMSCs (2 × 10^5^/treat), UMSC/*PD-1*, UMSC/*miR-124*, UMSC/*miR-124-PD-1 and isolated exosome from* UMSC/*miR-124-PD-1* (1 × 10^9^/treat). The MSCs for animal treatment were all be dissolved in 100 µL normal saline.

### Intra carotid artery injection (ICA)

To introduce engineered UMSCs into the brain, we employed intra-carotid artery (ICA) injection administration in C57B/L6 male mice. First, the mice were anesthetized using a Zoletil (25 mg/kg) plus xylazine (7.5 mg/kg) solution administered intraperitoneally. A 2 cm incision was made, starting 0.5 cm below the lower jaw and extending to the suprasternal fossa. Micro tweezers were used to carefully remove any muscle and connective tissue covering the right common carotid artery. Once the right CCA was exposed, the bottom of the CCA and the extra carotid artery (ECA) were ligated with 6.0 surgical sutures, respectively. To prevent backflow of blood, the upper section of the CCA was secured with a loop using a 6.0 surgical suture. A precise incision was made on the CCA using micro scissors. Subsequently, a PE-90 tube attached to a 30 g needle containing engineered UMSCs (100 µl volume) was gently inserted into the CCA incision. The tube’s position was secured with a micro arterial clamp during the injection process. After the cell injection, the upper loop of the CCA was ligated, and the incision was closed using surgical sutures.

To aid in the mice’s recovery, they were placed on a heating blanket. This meticulous procedure ensured the targeted delivery of engineered UMSCs into the brain, paving the way for further research and analysis.

### Immune cells validation from mice tissue

The single-cell suspensions isolated from the spleen, tumor-draining lymph nodes (TDLNs), and bone marrow were collected for flow cytometry analysis. The function of CD8 + T-cells (cytotoxic T lymphocytes, CTL) was determined based on the expressions of intracellular interferon (IFN)-γ and interleukin (IL)-2 using flow cytometry. Additionally, memory CTL was assayed by CD8^+^, CD62L^−^, and CD44^+^. CD11c^+^/CD24^+^/MHCII^+^ dendritic cells were assayed as positive markers. The accumulation of CD11b^+^CD86^+^ M1 type and CD11b^+^CD206^+^ M2 type of macrophage were also evaluated. Furthermore, the percentages of regulatory T cells (Tregs) and myeloid-derived suppressor cells (MDSCs) were used to evaluate immunosuppressive function, which plays a key role in the tumor microenvironment. Single-cell suspensions were stained with anti-FOXP3-Alexa Fluor 488/CD4-APC/CD25-PE antibodies using a mouse Treg flow kit according to the manufacturer’s protocol. CD11b-FITC and Gr-1-PE antibodies were used for detecting MDSCs. The percentages of these cell types were determined using the FACS Calibur flow cytometer, and data were analysed using FlowJo software [29].

### Biodistribution and homing ability of UMSCs

UMSC/*miR-124-PD-1* were harvested and incubated with a diluted DiR solution (320 µg/mL, XenoLight DiR cell tracing dye, PerkinElmer, Waltham, MA, USA) in PBS at 37 °C for 30 min. Mice bearing GL261 tumors was injected ICA with 2 × 10^5^ DiR-labeled UMSC/*miR-124-PD-1* 100 µL in PBS, and anaesthetised with 1–2% isoflurane 10 min before imaging. The DiR signal from UMSC/*miR-124-PD-1* was monitored with the IVIS Lumina LT system (Xenogen, PerkinElmer) at 2, 24, 48, 96 h after injection. The photons emitted from the tumor was assayed using the IVIS50 imaging system. The acquisition time was 30 s. Regions of interest (ROIs) were draw around the tumor and quantified using the Living Image software as photons·s^− 1^cm(2)-1·sr^− 1^.

### Hematoxylin and Eosin (H&E), immunohistochemistry (IHC), and Immunofluorescence (IHC) staining

Mice brains were fixed by transcardial perfusion with saline, followed by perfusion and immersion in 4% paraformaldehyde, and embedded in optimal cutting temperature compound (OCT). OCT-embedded brain tissue from mice was subjected to H&E or IHC or IF staining. All staining was performed according to routine protocols. For IF staining, OCT-embedded slices were permeabilized with 0.3% Triton X-100/PBS, blocked with 10% goat serum (Vector S-1000)/PBS, and incubated with primary antibodies diluted in 2% BSA/1× PBS overnight: luciferase (elabscience) (1∶100) and GFAP (elabscience) (1∶100). Thereafter, slices were incubated with Alexa Fluor 555 or Alexa 488-conjugated secondary antibodies (Jackson immunoresearch) for 30 min, followed by mounting with Prolong Gold antifade reagent containing DAPI for nuclear counter-staining. Slides were photographed with a Leica SP-8 confocal microscope, and images were processed with Image J. At least five slides from each group were analysed. The antibodies used in this material was listed in Supplementary Tables 2–3.

### Statistical analysis

Quantitative data are presented as the mean ± standard deviation (SD) from three independent experiments. Statistical significance was assessed using one-way analysis of variance (ANOVA) in GraphPad Prism version 7.0 (San Diego, CA), with a significance level of *p* < 0.05. To ensure reliability and reproducibility, each experiment was conducted independently at least three times, enabling robust comparisons between the control and treatment groups.

**Some of the materials and methods were listed in supported information.**

## Results

### miR-124 is identified as a potential target of CDK4/CDK6 in glioblastoma

**Fig. 1:**
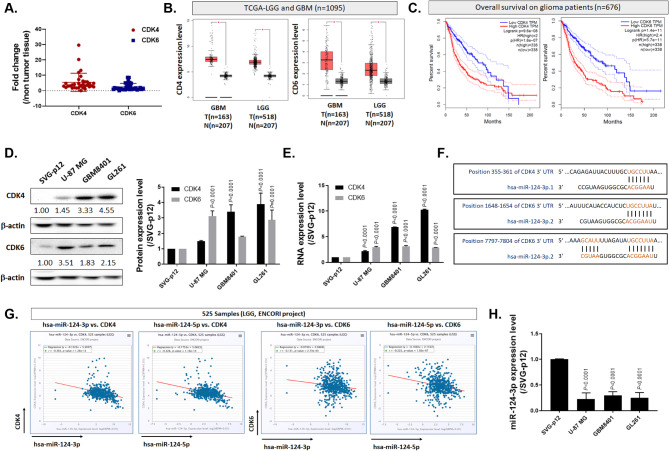
Identifying the Potential Role of CDK4 and CDK6 in GBM and Validating Them as Potential Target Factors Using miR-124. (**A**) RNA expression levels of *CDK4* and *CDK6* in GBM patients from Taiwan and (**B**) TCGA database assayed by RNA array and GEPIA platform. (**C**) Survival outcomes of GBM in different *CDK4* and *CDK6* expression levels. The dot line was represented as 95% confidence interval. (**D**) Protein and (**E**) RNA expression levels of *CDK4* and *CDK6* in glial cells and three different GBM cells. (**F**) The potential target region and the hypothesized regulation of miR-124 on both CDK4 and CDK6 assayed by TargetScan predicts tools. (**G**) The negative regulation between miR-124 and *CDK4/CDK6* in glioma assayed by ENCORI Pan-Cancer Analysis Platform. (**H**) The miR-124 expression in glial cells and three different GBM cells assayed by q-PCR

To identify the potential target, we conducted RNA microarray analysis on 38 glioblastoma samples obtained from Taipei Medical University, Taiwan (Fig. 1A). Notably, the expression levels of *CDK4* and *CDK6* were significantly elevated compared to normal brain tissue, suggesting their potential relevance for treatment. Subsequently, in Fig. 1B, we validated these findings by examining data from an open-source database (The Cancer Genome Atlas Program, TCGA), which also revealed high expression levels of *CDK4* and *CDK6* in GBM (glioblastoma). Furthermore, it was observed that patients exhibiting higher expression levels of *CDK4* and *CDK6* tended to have poorer survival outcomes (Fig. 1C). To delve deeper into the role of CDK4 and CDK6, we conducted a protein-level analysis involving one glial cell line (SVG-p12) and three GBM cell lines (U-87-MG, GBM8401, and GL261). Figure 1D illustrates that GBM8401 and GL261 cells exhibited notably higher protein expression levels of CDK4 and CDK6 compared to SVG-p12. Moreover, Fig. 1E demonstrates a similar pattern in RNA expression levels, with *CDK4* and *CDK6* being highly expressed in GBM cells. To target both CDK4 and CDK6, we conducted a search for potential binding miRNAs using the TargetScanHuman website. As illustrated in Fig. 1F, we identified binding sites for both CDK4 and CDK6 that can be matched with miR-124. CDK4 and CDK6, which play critical roles in regulating the cell cycle during the G1 phase, can serve as essential targets for controlling cell proliferation [25]. To further confirm the role of miR-124 in regulating CDK4 and CDK6, we conducted an interaction analysis using ENCORI. As shown in Fig. 1G, there is a negative correlation between the expression level of miR-124 and that of CDK4 and CDK6 in glioma samples. Additionally, we validated the expression of miR-124 through qPCR and confirmed that miR-124 exhibits relatively low expression in GBM cells compared to glial cells (Fig. 1H). In summary, our findings suggest that increasing the levels of miR-124 may represent a potential therapeutic strategy for addressing GBM. Additionally, developing the delivery strategies for miR-124 toward GBM is important.

### Elevating the expression of miR-124 in GBM cells has the potential to reduce their proliferation, metastatic tendencies and immunosuppressive regulation

To validate how miR-124 exerts control over GBM progression, we conducted transfections of GL261 and GBM8401 cells with miR-124 mimic. As depicted in the Supplementary Fig. 1A, this led to an increase in the expression level of miR-124-3p in GBM cells. Conversely, the expression levels of both *CDK4* and *CDK6* were reduced by the miR-124 mimic, as shown in Supplementary Fig. 1B. The expression of CDK4, CDK6, and cyclin D1 proteins was also reduced by mimic miR-124 (Supplementary Fig. 1C). Palbociclib treatment alone, used as a positive control in Supplementary Fig. 1D, suggests that CDK4/CDK6 inhibition may lead to G1 phase arrest. Furthermore, the overexpression of miR-124 appeared to decrease proliferation, as evidenced by a reduction in colony formation among GBM cells (Supplementary Fig. 2A). We also observed an increase in Annexin-V positive apoptotic cells in response to miR-124 mimic in GBM cells (Supplementary Fig. 2B). Moreover, mimic miR-124 was found to mitigate the SDF-1 induced invasion and migration abilities of GBM cells (Supplementary Fig. 3A-B). In addition, key metastasis-associated proteins such as VEGF, uPA, and MMP-9 were downregulated by miR-124 mimic in GBM cells (Supplementary Fig. 3A-C). The predicted binding sites of miR-124 on both STAT3 and NF-κB were validated using the TargetScanHuman website (Supplementary Fig. 4A). Notably, the phosphorylation of upstream transcription factors, STAT3 and NF-κB, was suppressed by miR-124 (Supplementary Fig. 4B). Furthermore, our findings suggest that miR-124 mimic may have the potential to suppress immunosuppressive factors such as IDO-1 and PD-L1 in GBM cells (Supplementary Fig. 4C). In conclusion, our findings suggest that miR-124 may regulate GBM progression by targeting the STAT3/NF-κB-mediated signaling pathway. Immunosuppressive factors in the tumor microenvironment can also be reduced by miR-124, hinting at the potential for combination with immunotherapy.

### The potential dual-gene delivery system and GBM apoptosis induction involving miR-124 and PD-1 using UMSCs

**Fig. 2:**
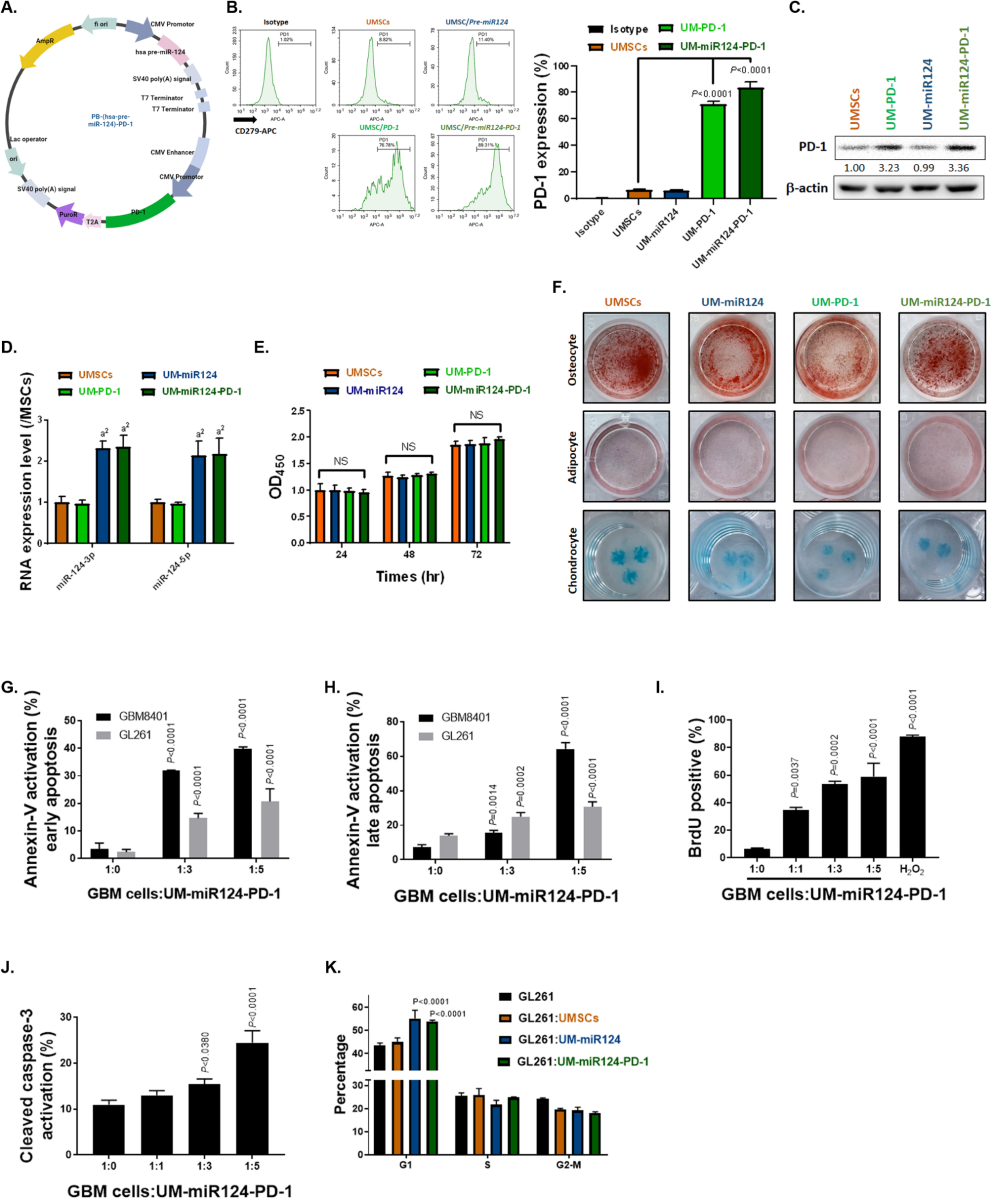
Characteristics of pre-miR-124 and PD-1 dual-gene-modified UMSCs and their capacity to induce apoptosis in GBM. (**A**) Construction of the pre-miR-124 and PD-1 dual-gene vector on PiggyBac transposon vectors. (**B**) Surface expression levels of PD-1 after electroporation with various vectors and selection of stable clones assayed by flow cytometry. (**C**) Protein expression of PD-1 in various vectors-transfected UMSCs assayed by Western blotting. (**D**) MiR-124 expression in various vectors-transfected UMSCs assayed by qPCR. (**E-F**) Proliferation and differentiation assays performed on various vectors-transfected UMSCs. (**G-H**) Early and late Annexin-V activation, (**I**) accumulation of BrdU, and (**J**) cleavage of caspase-3 in GBM cells co-cultured with different ratios of UMSC/*miR-124-PD-1* assayed by flow cytometry. (**K**) Cell cycle regulation of GBM cells when co-cultured with different ratios of UMSC/*miR-124-PD-1* assayed by flow cytometry

First and foremost, we designed a delivery system for miR-124 and PD-1 into UMSCs using the PiggyBac transposon vector (Fig. 2A). Following electroporation, we observed the expression of PD-1 (CD279) on the surface of transfected UMSCs (Fig. 2B). Additionally, we detected the protein expression of PD-1 in UMSC/*PD-1* and UMSC/*miR-124-PD-1* (Fig. 2C). Subsequently, we confirmed the expression of miR-124-3p in our transfected UMSC/*miR-124* and UMSC/*miR-124-PD-1* through qPCR analysis (Fig. 2D). To ensure that the characteristics of the transfected UMSCs remained unchanged after electroporation, we conducted proliferation assays and validated their differentiation capacity. As depicted in Fig. 2E, we observed a similar proliferation rate between wild-type UMSCs and those gene-modified with various vectors, including UMSC/*PD-1*, UMSC/*miR-124* and UMSC/*miR-124-PD-1*. Moreover, the osteocyte, adipocyte, and chondrocyte differentiation potential appeared to be consistent in both wild-type UMSCs and UMSCs gene-modified with various vectors (Fig. 2F). Figure 2G-H and Supplementary Fig. 5A demonstrates that the activation of Annexin-V may be dependent on the number UMSC/*miR-124-PD-1*. Specifically, a ratio of one GBM cell to five UMSC/*miR-124-PD-1* cells results in stronger Annexin-V activity compared to a ratio of one to three. Furthermore, an increase in the number of co-cultured UMSC/*miR-124-PD-1* with GL261 cells led to an increase in TUNEL-positive GBM cells (Fig. 2I and Supplementary Fig. 5B). The activation of cleaved caspase-3, a key marker of apoptosis, was also induced in GL261 cells by UMSC/*miR-124-PD-1* (Fig. 2J and Supplementary Fig. 5C). Notably, G1 arrest was observed in GL261 cells after co-culture with UMSC/*miR-124-PD-1* (Fig. 2K and Supplementary Fig. 5D). In summary, UMSC/*miR-124-PD-1* expresses both PD-1 and miR-124 and may also regulate apoptosis by modulating the cell cycle through the release of miR-124.

### The co-culture of exosomes derived from UMSC/*miR-124-PD-1* has the potential to induce apoptosis in GBM cells

**Fig. 3:**
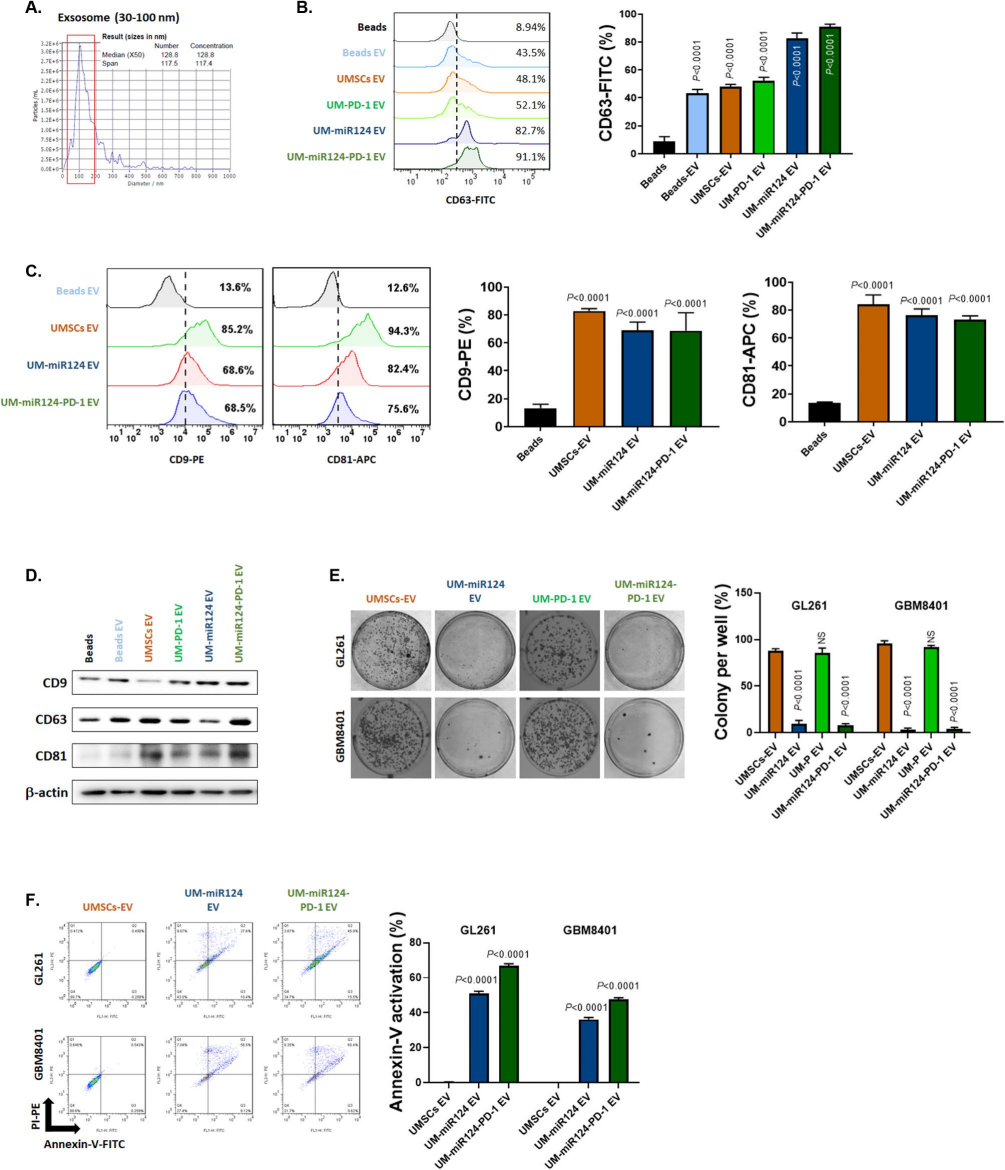
Characteristics of UMSC/*miR-124-PD-1* derived exosome and their capacity to induce apoptosis in GBM. (**A**) Evaluation of exosome size derived from UMSC/*miR-124-PD-1* by NTA. (**B-D**) Expression levels of CD9, CD63, CD81 on UMSC/*miR-124-PD-1*-derived exosomes assayed by flow cytometry and Western blotting. (**E**) Colony formation results in GBM cells co-cultured with exosomes derived from various vectors-transfected UMSCs. (**F**) Annexin-V activation in GBM cells co-cultured with exosomes derived from various vectors-transfected UMSCs assayed by flow cytometry

Furthermore, we isolated exosomes from both wild-type UMSCs and UMSCs gene-modified with various vectors. The median size of UMSC/*miR-124-PD-1* derived exosomes measured by NTA are 128.8 nm (Fig. 3A). To validate whether these isolated compounds could be identified as exosomes, we used CD63, a well-established exosome marker. Beads conjugated with or without commercial exosomes served as positive and negative controls. As shown in Fig. 3B, CD63 expression was notably high in the extracted exosomes from UMSCs and those gene-modified with various vectors, including UMSC/*PD-1*, UMSC/*miR-124* and UMSC/*miR-124-PD-1*. Surface expression of CD9 and CD81 was also detected in exosomes isolated from both UMSCs and all types of transfected UMSCs (Fig. 3C). Additionally, these cells were found to express CD9, CD63, and CD81 proteins (Fig. 3D). Colony formation decreased with exosomes isolated from UMSC/*miR-124* and UMSC/*miR-124-PD-1*, but not with those from UMSC/*PD-1*. Interestingly, colony count did not show significant changes in co-cultures with exosomes from UMSCs or UMSC/*PD-1*, suggesting that the tumor inhibition effect is primarily attributed to miR-124 rather than PD-1 (Fig. 3E). Additionally, we confirmed the activation of the Annexin-V signal through microscopy in GBM cells co-cultured with exosomes from UMSC/*miR-124* and UMSC/*miR-124-PD-1* (Fig. 3F and Supplementary Fig 6). We also conducted tests to determine whether these exosomes might affect normal glial cells (SVG-p12) when co-cultured together. As shown in supplementary Fig. 7, no Annexin-V activation was observed under co-culture conditions ranging from 1:1 to 1:5. This result suggests the safety of UMSC-derived exosomes. In summary, both UMSC/*miR-124-PD-1* and its derived exosomes exhibit the ability to suppress GBM progression, with the primary contribution coming from the release of miR-124.

### The migration, penetration, infiltration into GBM, and the bio-distribution of UMSC/*miR-124-PD-1*

**Fig. 4:**
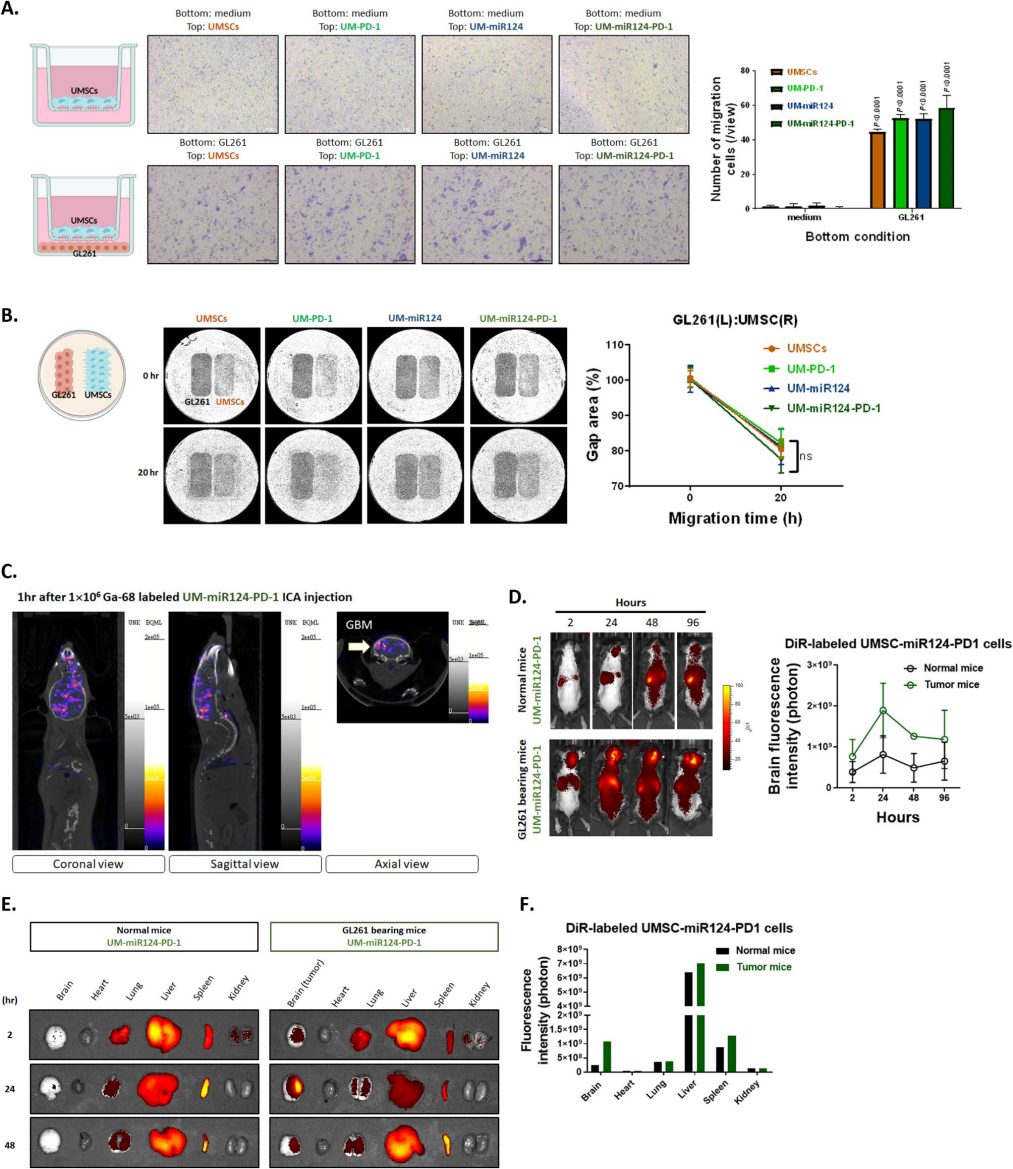
Infiltration, migration and bio-distribution characteristic of UMSC/*miR-124-PD-1*. The migration toward GBM cells is validated on various vectors-transfected UMSCs by (**A**) transwell assayed and (**B**) wound healing assay. (**C**) The infiltration of Ga-68 labeled UMSC/*miR-124-PD-1* after ICA injection is detected by SPECT/CT scan. (**D**) Whole body distribution of DiR labeled UMSC/*miR-124-PD-1* and its quantification signaling from brain area w/o tumor is determined by IVIS scan. (**E**) Ex vivo of DiR labeled UMSC/*miR-124-PD-1* signaling from different organs at 2, 24 and 48 h is determined by IVIS scan in tumor bearing and normal mice

Firstly, we aimed to determine whether UMSCs possess the capacity to transmigrate towards GBM cells. As illustrated in Fig. 4A, only the bottom well-seeded with GL261 groups could identify the migratory effect of all types of UMSCs. Furthermore, we employed a wound-healing assay to investigate whether UMSCs could also migrate toward GBM cells. After 20 h of incubation, UMSCs demonstrated a clear ability to migrate toward GBM cells, with no significant differences observed between the groups (Fig. 4B). To ensure effective inoculation of our gene-modified UMSCs into the brain area, we established an internal carotid artery (ICA) injection route for treatment. We verified our injection efficacy by administering Ga-68 labeled UMSC/*miR-124-PD-1* and conducting SPECT/CT scans after 2 h of injection. In Fig. 4C, we observed the accumulation of Ga-68 labeled UMSC/*miR-124-PD-1* in the brain tumor area, which was confirmed by the three-directional SPECT/CT images. These results indicate that our UMSC/*miR-124-PD-1* can successfully infiltrate towards GBM through ICA injection routes. Furthermore, we utilized DiR tracer to label our UMSC/*miR-124-PD-1* for dynamic tracking of its accumulation. As illustrated in Fig. 4D, the signal within the brain area is significantly higher in GBM-bearing mice compared to normal mice. The largest accumulation was observed at 24 h and began to decrease, reducing unnecessary accumulation. We also extracted organs from mice to perform ex vivo DiR detection in the brain, heart, lungs, livers, spleen, and kidney at different time points. In Fig. 4E, UMSC/*miR-124-PD-1* signal could only be detected in the brains of GBM-bearing mice as opposed to normal mice. Similar to the in vivo imaging, the highest signal of UMSC/*miR-124-PD-1* in the extracted brain is observed at 24 h after injection (Fig. 4F). In conclusion, UMSC/*miR-124-PD-1* demonstrated specific targeting ability towards GBM, which may reduce needless targeting of other organs.

### Effective Inhibition of GBM by UMSC/*miR-124-PD-1* and its derived exosomes

**Fig. 5:**
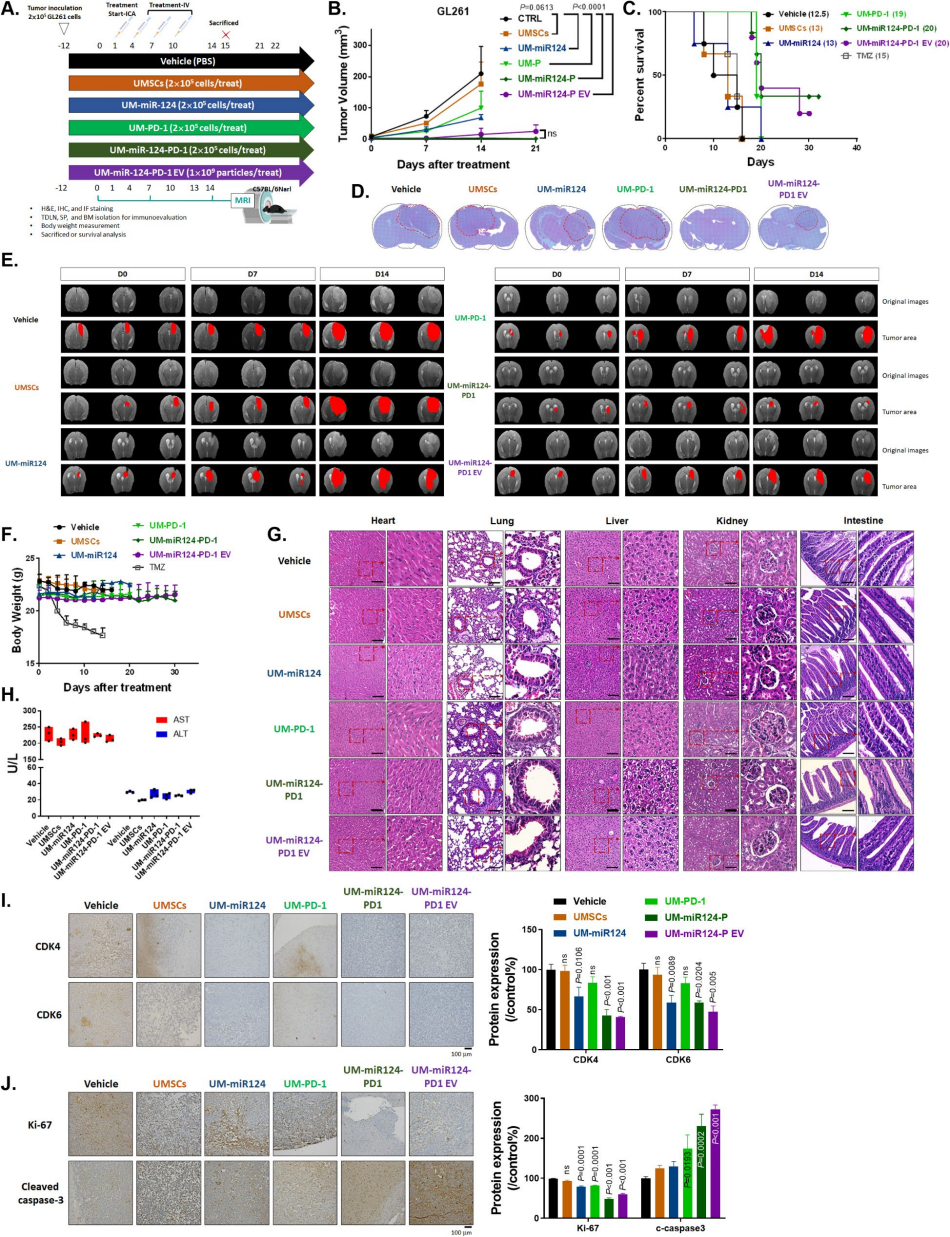
Therapeutic efficacy of UMSC/*miR-124-PD-1* and its exosome on GBM-bearing mice. (**A**) Experimental flowchart for treatment evaluation in the GBM model using UMSCs transfected with various vectors. The (**B**) tumor volume assessed by MR T2-RARE scan and (**C**) survival outcome calculated using the Kaplan-Meier method for UMSCs transfected with various vectors are presented. (**D**) Whole-brain H&E stain from one represented mice is displayed. (**E**) Representative MR tumor images with three consecutive slices from each group of mice are displayed. (**F**) The body weight of each group of mice during treatment is presented. (**G**) Pathological evaluation of normal organs from each group of mice isolated on day 15 using H&E staining. (**H**) Biochemical analysis of liver function evaluated from mice serum on Day 15. (**I-J**) Protein expression of CDK4, CDK6, Ki-67 and cleaved caspase-3 in tumor tissue are assayed by IHC staining

The experimental workflow for UMSC therapy in GL261-bearing mice is depicted in Fig. 5A. Cells or exosome particles were administered via a single internal carotid artery (ICA) injection and three intravenous [IV injections, each administered at three-day intervals, totaling four injections. Tumor size validation was conducted using MRI scans performed once a week. One group of mice underwent immunoregulation testing and was sacrificed on day 15, while another group was designated for survival analysis, with endpoints defined in accordance with ethical standards for animal experiments. Remarkably, significant tumor inhibition was observed in the UMSC/*miR-124-PD-1* and its derived exosomes treated group (Fig. 5B). Additionally, no apparent tumor induction was observed in the UMSCs treated group when compared to the non-treated control group. Moreover, not only tumor inhibition occur, but the survival outcome of the UMSC/*miR-124-PD-1* and its derived exosomes treated group was notably superior to that of the other groups (Fig. 5C). Compared to standard temozolomide (TMZ) treatment, UMSC/*miR-124-PD-1* and its derived exosomes exhibited significantly improved survival rates. The median survival for UMSC/*miR-124-PD-1* and its derived exosomes is 20 days, compared to 15 days for TMZ. Furthermore, a representative whole-brain H&E stain illustrated the anti-glioblastoma (GBM) progression efficacy of UMSC/*miR-124-PD-1* and its derived exosomes (Fig. 5D). No obvious tumors were found in the UMSC/*miR-124-PD-1* group, indicating the efficacy of the treatment. Figure 5E displays representative tumor progression from MRI scans of each group, further demonstrating the superior tumor-suppressive capacity of UMSC/*miR-124-PD-1* and its derived exosomes. The mean growth time to reach a 150 mm^3^ volume is nearly 100 times greater in UMSC/*miR-124-PD-1* compared to the vehicle (Supplementary Table 4). A synergistic tumor inhibition effect was observed in both UMSC/*miR-124-PD-1* and its derived exosomes compared to monotherapy (Supplementary Table 5). These treatments did not induce general toxicity in mice, as evidenced by the stability of mice body weight throughout the entire treatment process (Fig. 5F). However, TMZ treatment resulted in significant body weight fluctuations, underscoring its clinical limitations. Furthermore, no significant pathological alterations were observed in the heart, lungs, livers, kidney, and intestine (Fig. 5G). Importantly, MSCs are known to accumulate in lung or liver tissue; however, no evidence of potential damage was found. Serum from mice was also used to test functional markers of liver, AST, ALT and γGT, which showed no significant changes in any treatment condition (Fig. 5H and Supplementary Table 6). The kidney function marker, CREA, also remains consistent across all treatment groups (Supplementary Table 6). In tumor tissue, the expression levels of miR-124 targets, including CDK4 and CDK6, were reduced in UMSCs expressing miR-124 group (Fig. 5I). Moreover, in tumor IHC staining, the proliferation marker Ki-67 exhibited a decrease in response to treatment with UMSC/*miR-124-PD-1* and its derived exosomes (Fig. 5J). Conversely, the apoptosis marker, cleaved caspase-3, showed a corresponding increase. The successful delivery of our cells and exosomes into the brain tumor region was achieved through ICA injection combined with three IV injection methods, thereby reducing unnecessary accumulation in other organs.

### Immunostimulant effect of UMSC/*miR-124-PD-1* and its derived exosomes in vitro and in vivo

**Fig. 6:**
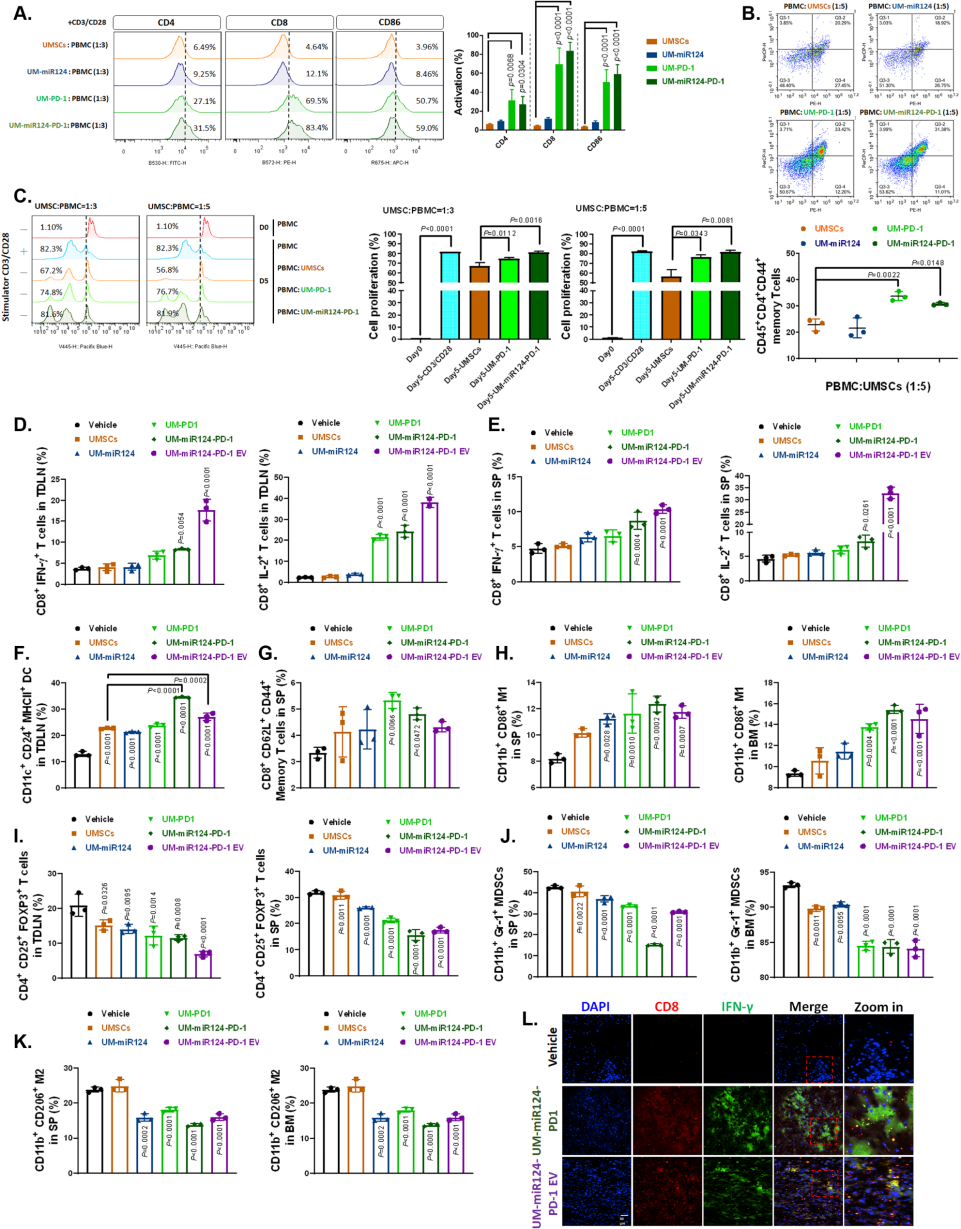
Immunoregulation of UMSC/*miR-124-PD-1* and its exosome on GBM-bearing mice. (**A**) CD4, CD8, CD86 induction signal, activation of (**B**) memory T cells, and (**C**) T cell proliferation in GBM co-cultured with UMSCs transfected with various vectors are assayed by flow cytometry. The accumulation of CD8^+^IFN-γ^+^ or CD8^+^IL-2^+^ CTL in mice (**D**) TDLN and (**E**) SP is assayed by flow cytometry. (**F**) CD11c^+^CD24^+^MHCII^+^ DCs in mice TDLN, (**G**) CD8^+^CD62L^+^CD44^+^ memory T cells in mice SP, and (**H**) CD11b^+^ CD86^+^ M1 in mice SP and BM are assayed by flow cytometry. (**I**) CD4^+^CD25^+^FOXP3^+^ Tregs in mice TDLN and SP, (**J**) CD11b^+^Gr-1^+^ MDSCs in mice SP and BM, and (**K**) CD11b^+^CD206^+^ M2 in mice SP and BM are assayed by flow cytometry. (**L**) CD8 and IFN-γ positive CTL cells are assayed by IF staining

To begin, we assessed the immunomodulatory potential of UMSC/*miR-124-PD-1* through co-culture with PBMCs. As depicted in Fig. 6A, the populations of CD4, CD8, and CD86 were observed to increase in the presence of UMSCs transfected with the PD-1 component, including UMSC/*PD-1* and UMSC/*miR-124-PD-1*. Additionally, the immunostimulatory effect of UMSC/*miR-124-PD-1* was found to have a long-term impact, as confirmed by the accumulation of CD45^+^CD4^+^CD44^+^ memory T cells (Fig. 6B). Furthermore, we validated that the proliferation of CD8^+^ T cells could be enhanced by varying the ratio of UMSC/*miR-124-PD-1*. In Fig. 6C, co-cultures of PBMCs and UMSC/*miR-124-PD-1* at a ratio of one to five demonstrated a more robust CD8^+^ T cell proliferation pattern compared to a ratio of one to three. In the subsequent steps, we conducted in vivo experiments to validate the immunoregulatory effects of various vector-transfected UMSCs. CD8^+^ T cells expressing IFN-γ or IL-2 from the tumor-draining lymph node (TDLN) were found to be activated in the UMSC/*miR-124-PD-1* and its exosomes treated group (Fig. 6D and Supplementary Fig. 8A). A similar activation pattern was also observed in splenocytes (SP), as depicted in Fig. 6E and supplementary Fig. 9A, for both the UMSC/*miR-124-PD-1* group and its exosomes group. Interestingly, UMSC/*miR-124-PD-1*-derived exosomes exhibited a superior cytotoxic T cell activation effect compared to the UMSCs themselves, which may be attributed to the exosomes’ enhanced tumor-penetration capabilities. In TDLN, CD11c^+^CD24^+^MHCII^+^ dendritic cells (DC) were activated by all types of transfected UMSCs (Fig. 6F and Supplementary Fig. 8B). Additionally, the accumulation of memory T cells in the SP was observed in UMSCs overexpressing PD-1 (Fig. 6G and Supplementary Fig. 9B). Furthermore, as shown in Fig. 6H, Supplementary Fig. 9C and Supplementary Fig. 10A, M1-like macrophages with anti-tumor potential were found to accumulate in response to all types of transfected UMSCs in both the SP and bone marrow (BM). We not only conducted isolated immune-related organ assessments but also validated the accumulation patterns of tumor-infiltrating immune cells within the tumor tissue using IF and IHC staining. As illustrated in Fig. 6L, supplementary Fig. 11A and 11C, the accumulation of CD8/IFN-γ T cells and CD86 M1 macrophage was significantly higher in the UMSC/*miR-124-PD-1* treated group compared to other treatments. In tumor IHC-stained tissue, the expression of CD8 and CD86 was elevated in the UMSC/*miR-124-PD-1* and its derived exosomes group (Supplementary Fig. 12A and C). In summary, UMSC/*miR-124-PD-1* and the exosomes derived from them have the potential to induce positive immunoregulation in GBM effectively.

### Immunosuppressive effect of UMSC/*miR-124-PD-1* and its derived exosomes in vitro and in vivo

In addition to observing the immunostimulatory function of gene-modified UMSCs, we also validated their impact on immunosuppressive cells. Tregs (CD4^+^CD25^+^FOXP3^+^) in TDLN and SP were found to be diminished by all types of transfected UMSCs (Fig. 6I, supplementary Fig. 8C and 9D). Furthermore, myeloid-derived suppressor cells (MDSCs) were effectively suppressed by the UMSC/*miR-124-PD-1* and its exosomes-treated group in spleen and bone marrow (Fig. 6J and supplementary Fig. 9E and 10B). The opposing function of M1, recognized as tumor-associated macrophages (M2 type), was also decreased by the UMSC/*miR-124-PD-1* and its exosomes-treated group in SP and BM (Fig. 6K, Supplementary Fig. 9C and 10 A). The accumulation of CD4/FOXP3 Treg cells and CD206 M2 macrophages in the tumor, as stained by IF, was significantly lower in the UMSC/*miR-124-PD-1* treated group compared to other treatments (Supplementary Fig. 11B-C). The immunosuppressive factors, such as IDO, FOXP3, and CD206, which were stained by IHC, were also found to be decreased in mice tumor tissue by UMSC/*miR-124-PD-1* and its derived exosomes Supplementary Fig. 12B and 12 C). In summary, the immunosuppression within the TME was effectively controlled by UMSC/*miR-124-PD-1* and its exosomes by reducing activation and accumulating immunosuppressive cells.

## Discussion

The standard-of-care therapy currently results in a median survival rate of only 12–15 months for the aggressive disease, glioblastoma. Consequently, there is a pressing unmet need for innovative strategies to effectively address the treatment challenges. Some of the primary obstacles hindering progress include the aggressive growth of tumors in vital organs, rendering local or monotherapy less effective. Additionally, the presence of the BBB shielding tumor cells [30], their inherent resistance to apoptosis, and the absence of a singular, targetable oncogenic pathway all pose formidable hurdles for treatment. In this study, our objective is to leverage the unique capabilities of UMSCs to deliver two potential therapeutic genes (miR-124 and PD-1) directly into the tumor. UMSCs and its derived exosome have the natural ability to infiltrate within the tumor, making them a promising vehicle for targeted therapy [31]. It’s not only the gene-modified UMSCs themselves that exhibit genetic alterations, but their secreted exosomes also carry similar genetic modifications, which demonstrate specific efficacy against cancer [32, 33].

Exosomes, nanosized extracellular vesicles released by various cells, are more easily internalized when they exhibit a broad size distribution, as smaller exosomes are favored by cells [34]. Engineered exosomes have shown promising results in preclinical studies for regenerating bone, cartilage, skin, cardiac, neuronal, and other tissues, highlighting their potential as biomaterials for delivering therapeutic genes or drugs [35]. Studies have shown that exosomes transport RNA more effectively than other nanoparticles, enhancing the functional efficiency of small RNA delivery [36]. Liang et al. and Limoni et al. have successfully delivered miRNA and siRNA to target colon and breast cancer, respectively [37, 38]. The Ohno group also successfully utilized gene-modified exosomes as biomaterials to deliver miRNA to EGFR-expressing breast cancer [39]. Exosomes are emerging as next-generation platforms for nanomedicine in cancer therapy; therefore, evaluating their targeting capabilities for specific cancers is crucial for developing exosome-based treatments.

In recent years, cell cycle-related genes have been recognized as potential targets for cancer treatment [40, 41]. In Fig. 1, it is important to emphasize that CDK4 and CDK6 display elevated expression levels in GBM samples and hold significant associations with survival outcomes. We then specifically identified CDK4 and CDK6 as pivotal targets under the regulation of miR-124 (Fig. 1F). Importantly, miR-124 exhibits relatively low expression in GBM cells when compared to normal glial cells, underscoring its potential specificity as a treatment target (Fig. 1H). In addition, preclinical model suggested that the ectopic expression of mature miR-124 in a GBM cell line resulted in significant inhibition of migration and invasion, demonstrating a role for miR-124 in promoting tumour invasiveness [42]. After mimic miR-124 on highly CDK4 and 6 expressed GBM cells, the proliferation, and invasion/migration were suppressed (Supplementary Figs. 1–4). It has been demonstrated that the overexpression of miR-124 increases apoptosis in a colon cancer model [43]. In Figs. 2E and 3I-L, the apoptosis of GBM cells after mimic miR-124 or co-cultured with our UMSC/*miR-124-PD-1* are also confirmed. There also some evidence indicated miR-124 regulates cell migration and proliferation were attributed to its targeting of Ras/Raf/ERK1/2 [44]. Our group indicated the regulation of tumor inhibition by miR-124 is associated with inactivation of STAT3 and NF-κB mediated signaling transduction (Supplementary Fig. 4A-B). Lee et al., demonstrated that the miR-124 mimics delivered by the bone marrow- and adipose-MSCs may decrease the migration of the U87 cells and the self-renewal of the glioma stem cells, respectively, suggesting that the MSCs were able to transfer the exogenous miRNAs in an efficient and functional way [45]. Additionally, Sharif’s group also proved that MSCs can delivery exogenous miR-124 to suppress the cells migration and to increase the sensitization of chemotherapy of GBM [46]. While the studies mentioned above suggested the potential of targeting miR-124, they did not include an in vivo therapeutic evaluation model or an assessment of survival outcomes in GBM. Figure 5A-E illustrates that UMSCs delivering miR-124 exhibited tumor inhibition and extended survival, whereas single-target approaches remained limited in their effectiveness. Moreover, the infiltrated potential and delivery efficacy within tumor area was confirmed by transwell assay, wound healing assay, Ga-68- and DiR- labeled UMSC/*miR-124-PD-1* (Fig. 4) that support the tumor targeting effect of UMSCs.

Prior to 2015, the central nervous system (CNS) was commonly regarded as an “immune privileged” organ. This notion was based on the presence of an intact BBB and the absence of a lymphatic system, which limited immune responses within the CNS [47]. However, Louveau’s group made a groundbreaking discovery by revealing the existence of a CNS lymphatic system [48]. Nonetheless, the presence of immune suppressive cells and the expression of immune checkpoint molecules represent two major factors that enable the tumor to elude immune surveillance [49, 50]. The expression of PD-L1, along with the accumulation of Tregs and MDSCs, is associated with a poor prognosis and the induction of apoptosis in activated cytotoxic T-cells [51, 52]. While monotherapy with ICIs has limitations, numerous reports have suggested that combining immune checkpoint inhibitors with conventional treatments for GBM, such as radiation or chemotherapy, may enhance therapeutic efficacy [53]. PD-1 acts as a brake on the immune system by interacting with its ligands, PD-L1 and PD-L2, which are expressed on tumor cells, dendritic cells, and other immune cells [54, 55]. To counteract this, PD-1 antibodies were developed to restore T cell activity by blocking the PD-L1 signaling from tumors [56, 57]. In our model, the tumor-tropic PD-1-expressing UMSC may mimic the function of anti-PD-1 antibodies, thereby reducing the interaction between tumor cells and T cells and boosting T cell activity. In this study, we indicated that a positive immune regulation can be achieved by delivering PD-1 via UMSCs to block the interaction between PD-L1 and T cells. Both co-culture and in vivo systems demonstrated the activation of CTLs, dendritic cells (DCs), memory T cells, and M1 macrophages in the presence of UMSCs expressing PD-1 (Fig. 6A-H and L). Conversely, the population of immune regulatory cells with a negative impact, such as Tregs, MDSCs, and M2 macrophages, was reduced by UMSC/*miR-124-PD-1* administration (Fig. 6I-K). In addition to the systemic immunoregulation of UMSC/*miR-124-PD-1*, we also observed a distinct immune cell accumulation pattern within the tumor area.

Due to the characteristics of UMSCs, here, we proposed that UMSCs can be cell carriers to deliver multi-targeted vectors, which may markedly enhance the therapeutic efficacy against this lethal disease. We successfully established UMSCs transduced dual functions vector, including miR-124 for tumor suppression and PD-1 expression vector for PD-L1 blockage. We confirmed that our UMSC/*miR-124-PD-1* and its exosome may enhance the antitumor activity and suppress the immunosuppression effect of tumors (Figs. 5 and 6). Our breakthrough is the successful development of an innovative biomaterial delivery platform that employs UMSCs for dual gene therapy, potentially extending the survival of GBM patients.

## Electronic supplementary material

Below is the link to the electronic supplementary material.

Supplementary Material 1

Supplementary Material 2

## Data Availability

No datasets were generated or analysed during the current study.

## Declarations

### Studies involving animal subjects

The animal study conducted in this research was reviewed and approved by the Institutional Animal Care and Use Committee (IACUC) at China Medical University, Taichung, Taiwan (ID: CMU CMUIACUC-2019-042).

### Inclusion of identifiable human data

There is no inclusion of identifiable human data in the manuscript.

## References

[1] Wu W, Klockow JL, Zhang M, Lafortune F, Chang E, Jin L, Wu Y, Daldrup-Link HE. Glioblastoma multiforme (GBM): an overview of current therapies and mechanisms of resistance. Pharmacol Res. 2021;171:105780. 34302977 10.1016/j.phrs.2021.105780 PMC8384724

[2] Nozhat Z, Heydarzadeh S, Shahriari-Khalaji M, Wang S, Iqbal MZ, Kong X. Advanced biomaterials for human glioblastoma multiforme (GBM) drug delivery. Biomaterials Sci. 2023;11(12):4094–131. 10.1039/d2bm01996e 37073998

[3] Abdul-Al M, Saeinasab M, Zare A, Barati M, Shakeri S, Keykhosravi E, Momeni-Moghaddam M, Najafzadeh M, Keshel SH, Farzi G, Sefat F. Application of biomaterials for glioblastoma treatment: promises, advances, and challenges. Mater Today Commun. 2022;33:104562.

[4] Yao Z, Jiang X, Yao H, Wu Y, Zhang F, Wang C, Qi C, Zhao C, Wu Z, Qi M, Zhang J, Cao X, Wang Z, Wu F, Yao C, Liu S, Ling S, Xia H. Efficiently targeted therapy of glioblastoma xenograft via multifunctional biomimetic nanodrugs. Biomaterials Res. 2022;26(1):71. 10.1186/s40824-022-00309-y PMC9717509 36461108

[5] Xu H-Z, Li T-F, Ma Y, Li K, Zhang Q, Xu Y-H, Zhang Y-C, Zhao L, Chen X. Targeted photodynamic therapy of glioblastoma mediated by platelets with photo-controlled release property. Biomaterials. 2022;290:121833. 36201945 10.1016/j.biomaterials.2022.121833

[6] Xiao M, Shi Y, Jiang S, Cao M, Chen W, Xu Y, Xu Z, Wang K. Recent advances of nanomaterial-based anti-angiogenic therapy in tumor vascular normalization and immunotherapy. Front Oncol 12 (2022). 10.3389/fonc.2022.1039378 PMC9745116 36523993

[7] Grauer O, Jaber M, Hess K, Weckesser M, Schwindt W, Maring S, Wölfer J, Stummer W. Combined intracavitary thermotherapy with iron oxide nanoparticles and radiotherapy as local treatment modality in recurrent glioblastoma patients. J Neurooncol. 2019;141(1):83–94. 30506500 10.1007/s11060-018-03005-x PMC6341053

[8] Ezike TC, Okpala US, Onoja UL, Nwike CP, Ezeako EC, Okpara OJ, Okoroafor CC, Eze SC, Kalu OL, Odoh EC, Nwadike UG, Ogbodo JO, Umeh BU, Ossai EC. Nwanguma, advances in drug delivery systems, challenges and future directions. Heliyon. 2023;9(6):e17488. 37416680 10.1016/j.heliyon.2023.e17488 PMC10320272

[9] Elumalai K, Srinivasan S, Shanmugam A. Review of the efficacy of nanoparticle-based drug delivery systems for cancer treatment. Biomedical Technol. 2024;5:109–22.

[10] Zhang T, Lin R, Wu H, Jiang X, Gao J. Mesenchymal stem cells: A living carrier for active tumor-targeted delivery. Adv Drug Deliv Rev. 2022;185:114300. 35447165 10.1016/j.addr.2022.114300

[11] Kim DW, Staples M, Shinozuka K, Pantcheva P, Kang SD, Borlongan CV. Wharton’s jelly-derived mesenchymal stem cells: phenotypic characterization and optimizing their therapeutic potential for clinical applications. Int J Mol Sci. 2013;14(6):11692–712. 23727936 10.3390/ijms140611692 PMC3709752

[12] Mebarki M, Abadie C, Larghero J, Cras A. Human umbilical cord-derived mesenchymal stem/stromal cells: a promising candidate for the development of advanced therapy medicinal products. Stem Cell Res Ther. 2021;12(1):152. 33637125 10.1186/s13287-021-02222-y PMC7907784

[13] Cunha Silva L, Branco F, Cunha J, Vitorino C, Gomes C, Carrascal MA, Falcão A, Miguel Neves B. Teresa Cruz, the potential of exosomes as a new therapeutic strategy for glioblastoma. Eur J Pharm Biopharm. 2024;203:114460. 39218361 10.1016/j.ejpb.2024.114460

[14] Zeng YF, Wei XY, Guo QH, Chen SY, Deng S, Liu ZZ, Gong ZC, Zeng WJ. The efficacy and safety of anti-PD-1/PD-L1 in treatment of glioma: a single-arm meta-analysis. Front Immunol. 2023;14:1168244. 37122727 10.3389/fimmu.2023.1168244 PMC10140424

[15] Segura-Collar B, Hiller-Vallina S, de Dios O, Caamaño-Moreno M, Mondejar-Ruescas L, Sepulveda-Sanchez JM, Gargini R. Advanced immunotherapies for glioblastoma: tumor neoantigen vaccines in combination with immunomodulators. Acta Neuropathol Commun. 2023;11(1):79. 37165457 10.1186/s40478-023-01569-y PMC10171733

[16] Wu L, Zhang Z, Bai M, Yan Y, Yu J, Xu Y. Radiation combined with immune checkpoint inhibitors for unresectable locally advanced non-small cell lung cancer: synergistic mechanisms, current State, challenges, and orientations. Cell Communication Signal. 2023;21(1):119. 10.1186/s12964-023-01139-8 PMC10207766 37221584

[17] Gong J, Le TQ, Massarelli E, Hendifar AE, Tuli R. Radiation therapy and PD-1/PD-L1 Blockade: the clinical development of an evolving anticancer combination. J Immunother Cancer. 2018;6(1):46. 29866197 10.1186/s40425-018-0361-7 PMC5987486

[18] Ordóñez-Rubiano EG, Rincón-Arias N, Espinosa S, Shelton WJ, Salazar AF, Cómbita A, Baldoncini M, Luzzi S, Payán-Gómez C, Gómez- DF, Amarillo F, Hakim JG, Patiño-Gómez R. Parra- Medina, the potential of miRNA-based approaches in glioblastoma: an update in current advances and future perspectives. Curr Res Pharmacol Drug Discovery. 2024;7:100193. 10.1016/j.crphar.2024.100193 PMC11268206 39055532

[19] Moghadasi M, Alivand M, Fardi M, Moghadam KS, Solali S. Emerging molecular functions of microRNA-124: Cancer pathology and therapeutic implications. Pathol - Res Pract. 2020;216(3):152827. 31983567 10.1016/j.prp.2020.152827

[20] Silber J, Lim DA, Petritsch C, Persson AI, Maunakea AK, Yu M, Vandenberg SR, Ginzinger DG, James CD, Costello JF, Bergers G, Weiss WA, Alvarez-Buylla A, Hodgson JG. miR-124 and miR-137 inhibit proliferation of glioblastoma multiforme cells and induce differentiation of brain tumor stem cells. BMC Med. 2008;6:14. 18577219 10.1186/1741-7015-6-14 PMC2443372

[21] Mucaj V, Lee SS, Skuli N, Giannoukos DN, Qiu B, Eisinger-Mathason TS, Nakazawa MS, Shay JE, Gopal PP, Venneti S, Lal P, Minn AJ, Simon MC, Mathew LK. MicroRNA-124 expression counteracts pro-survival stress responses in glioblastoma. Oncogene. 2015;34(17):2204–14. 24954504 10.1038/onc.2014.168 PMC4275412

[22] Gourishetti K, Balaji Easwaran V, Mostakim Y, Ranganath Pai KS, Bhere D. MicroRNA (miR)-124: A Promising Therapeutic Gateway for Oncology, Biology, 2023. 10.3390/biology12070922 PMC10376116 37508353

[23] Martino E, D’Onofrio N, Anastasio C, Abate M, Zappavigna S, Caraglia M, Balestrieri ML. MicroRNA-nanoparticles against cancer: opportunities and challenges for personalized medicine. Mol Therapy - Nucleic Acids. 2023;32:371–84. 10.1016/j.omtn.2023.03.021 PMC10148042 37128277

[24] Oveili E, Vafaei S, Bazavar H, Eslami Y, Mamaghanizadeh E, Yasamineh S, Gholizadeh O. The potential use of mesenchymal stem cells-derived exosomes as MicroRNAs delivery systems in different diseases. Cell Communication Signal. 2023;21(1):20. 10.1186/s12964-022-01017-9 PMC9869323 36690996

[25] Dilsiz N. A comprehensive review on recent advances in exosome isolation and characterization: toward clinical applications. Translational Oncol. 2024;50:102121. 10.1016/j.tranon.2024.102121 PMC11418158 39278189

[26] Reclusa P, Verstraelen P, Taverna S, Gunasekaran M, Pucci M, Pintelon I, Claes N, de Miguel-Pérez D, Alessandro R, Bals S, Kaushal S, Rolfo C. Improving extracellular vesicles visualization: from static to motion. Sci Rep. 2020;10(1):6494. 32300120 10.1038/s41598-020-62920-0 PMC7162928

[27] Huang HS, Chiang IT, Lawal B, Weng YS, Jeng LB, Kuo YC, Liu YC, Hsu FT. A novel Isotope-labeled small molecule probe CC12 for Anti-glioma via suppressing LYN-mediated progression and activating apoptosis pathways. Int J Biol Sci. 2023;19(10):3209–25. 37416766 10.7150/ijbs.82266 PMC10321274

[28] Chiang IT, Liu YC, Liu HS, Ali AAA, Chou SY, Hsu TI, Hsu FT. Regorafenib reverses Temozolomide-Induced CXCL12/CXCR4 signaling and triggers apoptosis mechanism in glioblastoma. Neurotherapeutics. 2022;19(2):616–34. 35267171 10.1007/s13311-022-01194-y PMC9226247

[29] Yueh PF, Chiang CS, Tsai IJ, Tseng YL, Chen HR, Lan KL, Hsu FT. A multifunctional pegylated liposomal-encapsulated Sunitinib enhancing autophagy, Immunomodulation, and safety in renal cell carcinoma. J Nanobiotechnol. 2024;22(1):459. 10.1186/s12951-024-02664-5 PMC11293195 39085911

[30] Wu D, Chen Q, Chen X, Han F, Chen Z, Wang Y. The blood–brain barrier: structure, regulation and drug delivery. Signal Transduct Target Therapy. 2023;8(1):217. 10.1038/s41392-023-01481-w PMC10212980 37231000

[31] Aravindhan S, Ejam SS, Lafta MH, Markov A, Yumashev AV, Ahmadi M. Mesenchymal stem cells and cancer therapy: insights into targeting the tumour vasculature. Cancer Cell Int. 2021;21(1):158. 33685452 10.1186/s12935-021-01836-9 PMC7938588

[32] Bagheri E, Abnous K, Farzad SA, Taghdisi SM, Ramezani M, Alibolandi M. Targeted doxorubicin-loaded mesenchymal stem cells-derived exosomes as a versatile platform for fighting against colorectal cancer. Life Sci. 2020;261:118369. 32882265 10.1016/j.lfs.2020.118369

[33] Shams F, Pourjabbar B, Hashemi N, Farahmandian N, Golchin A, Nuoroozi G, Rahimpour A. Current progress in engineered and nano-engineered mesenchymal stem cells for cancer: from mechanisms to therapy. Biomed Pharmacother. 2023;167:115505. 37716113 10.1016/j.biopha.2023.115505

[34] Kar R, Dhar R, Mukherjee S, Nag S, Gorai S, Mukerjee N, Mukherjee D, Vatsa R, Chandrakanth Jadhav M, Ghosh A, Devi A, Krishnan A, Thorat ND. Exosome-Based smart drug delivery tool for Cancer theranostics. ACS Biomaterials Sci Eng. 2023;9(2):577–94. 10.1021/acsbiomaterials.2c01329 PMC9930096 36621949

[35] Hu W, Wang W, Chen Z, Chen Y, Wang Z. Engineered exosomes and composite biomaterials for tissue regeneration. Theranostics. 2024;14(5):2099–126. 38505616 10.7150/thno.93088 PMC10945329

[36] Tai YL, Chen KC, Hsieh JT, Shen TL. Exosomes in cancer development and clinical applications. Cancer Sci. 2018;109(8):2364–74. 29908100 10.1111/cas.13697 PMC6113508

[37] Liang G, Zhu Y, Ali DJ, Tian T, Xu H, Si K, Sun B, Chen B, Xiao Z. Engineered exosomes for targeted co-delivery of miR-21 inhibitor and chemotherapeutics to reverse drug resistance in colon cancer. J Nanobiotechnol. 2020;18(1):10. 10.1186/s12951-019-0563-2 PMC6950820 31918721

[38] Limoni SK, Moghadam MF, Moazzeni SM, Gomari H, Salimi F. Engineered exosomes for targeted transfer of SiRNA to HER2 positive breast Cancer cells. Appl Biochem Biotechnol. 2019;187(1):352–64. 29951961 10.1007/s12010-018-2813-4

[39] Ohno S, Takanashi M, Sudo K, Ueda S, Ishikawa A, Matsuyama N, Fujita K, Mizutani T, Ohgi T, Ochiya T, Gotoh N, Kuroda M. Systemically injected exosomes targeted to EGFR deliver antitumor MicroRNA to breast cancer cells. Mol Ther. 2013;21(1):185–91. 23032975 10.1038/mt.2012.180 PMC3538304

[40] Suski JM, Braun M, Strmiska V, Sicinski P. Targeting cell-cycle machinery in cancer. Cancer Cell. 2021;39(6):759–78. 33891890 10.1016/j.ccell.2021.03.010 PMC8206013

[41] McCord M, Jamshidi P. Targeting the cell cycle to enhance chemotherapy efficacy in glioblastoma. Neurooncology. 2024;26(6):1097–8. 10.1093/neuonc/noae062 PMC11145455 38517031

[42] Fowler A, Thomson D, Giles K, Maleki S, Mreich E, Wheeler H, Leedman P, Biggs M, Cook R, Little N, Robinson B, McDonald K. miR-124a is frequently down-regulated in glioblastoma and is involved in migration and invasion. Eur J Cancer. 2011;47(6):953–63. 21196113 10.1016/j.ejca.2010.11.026

[43] Zhang J, Lu Y, Yue X, Li H, Luo X, Wang Y, Wang K, Wan J. MiR-124 suppresses growth of human colorectal cancer by inhibiting STAT3. PLoS ONE. 2013;8(8):e70300. 23940556 10.1371/journal.pone.0070300 PMC3734178

[44] Gourishetti K, Balaji Easwaran V, Mostakim Y, Ranganath Pai KS, Bhere D. MicroRNA (miR)-124: A promising therapeutic gateway for oncology. Biology (Basel) 12(7) (2023). 10.3390/biology12070922 PMC10376116 37508353

[45] Nowak B, Rogujski P, Janowski M, Lukomska B, Andrzejewska A. Mesenchymal stem cells in glioblastoma therapy and progression: how one cell does it all, biochimica et biophysica acta (BBA) -. Reviews Cancer. 2021;1876(1):188582. 10.1016/j.bbcan.2021.188582 34144129

[46] Sharif S, Ghahremani MH, Soleimani M. Delivery of exogenous miR-124 to glioblastoma multiform cells by Wharton’s jelly mesenchymal stem cells decreases cell proliferation and migration, and confers chemosensitivity. Stem Cell Rev Rep. 2018;14(2):236–46. 29185191 10.1007/s12015-017-9788-3

[47] Carson MJ, Doose JM, Melchior B, Schmid CD, Ploix CC. CNS immune privilege: hiding in plain sight. Immunol Rev. 2006;213:48–65. 16972896 10.1111/j.1600-065X.2006.00441.x PMC2633103

[48] Louveau A, Smirnov I, Keyes TJ, Eccles JD, Rouhani SJ, Peske JD, Derecki NC, Castle D, Mandell JW, Lee KS, Harris TH, Kipnis J. Structural and functional features of central nervous system lymphatic vessels. Nature. 2015;523(7560):337–41. 26030524 10.1038/nature14432 PMC4506234

[49] Vimalathas G, Kristensen BW. Expression, prognostic significance and therapeutic implications of PD-L1 in gliomas. Neuropathol Appl Neurobiol. 2022;48(1):e12767. 34533233 10.1111/nan.12767 PMC9298327

[50] Xue S, Song G, Yu J. The prognostic significance of PD-L1 expression in patients with glioma: A meta-analysis. Sci Rep. 2017;7(1):4231. 28652622 10.1038/s41598-017-04023-x PMC5484664

[51] DiDomenico J, Lamano JB, Oyon D, Li Y, Veliceasa D, Kaur G, Ampie L, Choy W, Lamano JB, Bloch O. The immune checkpoint protein PD-L1 induces and maintains regulatory T cells in glioblastoma. Oncoimmunology. 2018;7(7):e1448329. 29900065 10.1080/2162402X.2018.1448329 PMC5993506

[52] Kumar R, de Mooij T, Peterson TE, Kaptzan T, Johnson AJ, Daniels DJ, Parney IF. Modulating glioma-mediated myeloid-derived suppressor cell development with Sulforaphane. PLoS ONE. 2017;12(6):e0179012. 28666020 10.1371/journal.pone.0179012 PMC5493295

[53] Dovedi SJ, Adlard AL, Lipowska-Bhalla G, McKenna C, Jones S, Cheadle EJ, Stratford IJ, Poon E, Morrow M, Stewart R, Jones H, Wilkinson RW, Honeychurch J, Illidge TM. Acquired resistance to fractionated radiotherapy can be overcome by concurrent PD-L1 Blockade. Cancer Res. 2014;74(19):5458–68. 25274032 10.1158/0008-5472.CAN-14-1258

[54] Iwai Y, Hamanishi J, Chamoto K, Honjo T. Cancer immunotherapies targeting the PD-1 signaling pathway. J Biomed Sci. 2017;24(1):26. 28376884 10.1186/s12929-017-0329-9 PMC5381059

[55] Pardoll DM. The Blockade of immune checkpoints in cancer immunotherapy. Nat Rev Cancer. 2012;12(4):252–64. 22437870 10.1038/nrc3239 PMC4856023

[56] Cha JH, Chan LC, Li CW, Hsu JL, Hung MC. Mechanisms controlling PD-L1 expression in cancer. Mol Cell. 2019;76(3):359–70. 31668929 10.1016/j.molcel.2019.09.030 PMC6981282

[57] Cui JW, Li Y, Yang Y, Yang HK, Dong JM, Xiao ZH, He X, Guo JH, Wang RQ, Dai B, Zhou ZL. Tumor immunotherapy resistance: revealing the mechanism of PD-1 / PD-L1-mediated tumor immune escape. Biomed Pharmacother. 2024;171:116203. 38280330 10.1016/j.biopha.2024.116203

