# Supplementary material for: Innovative dual-gene delivery platform using miR-124 and PD-1 via umbilical cord mesenchymal stem cells and exosome for glioblastoma therapy
Source: J Exp Clin Cancer Res. 2025 Mar 25;44:107. doi: 10.1186/s13046-025-03336-4 (PMC11934454; doi:10.1186/s13046-025-03336-4)

**Supplementary materials**

**Mimic miR-124-3p transfection**

GL261 and GBM-8401 cells were seeded into 6-well plates at a density of 3×10^5^ cells per well and incubated overnight at 37 °C with 5% CO_2_ before transfection. The mixture containing the dilution of mimic miR-124-3p and X-tremeGENE™ 360 Transfection Reagent (Sigma-Aldrich, St. Louis, USA) was incubated for 20 minutes at room temperature. The miRNA mixture was then added to the cells in a dropwise manner. After further incubation for 48 hours at 37 °C, the cells were collected for subsequent experiments.

**UMSCs Isolation, culture and differentiation**

Fresh human umbilical cords (n = 6) were collected after full-term births with informed consent, following the guidelines approved by the Institutional Review Board at China Medical University Hospital and China Medical University (certification no. CMUH107-REC1-176). Umbilical cord segments were longitudinally cut, cord vessels manually removed, and the underlying perivascular tissue was cut into small explants, which were then placed directly in 35 mm tissue culture plastic dishes. The culture medium used was KnockOut Dulbecco's Modified Eagle's Medium with 4.5 g/L glucose (Life Technologies), 2 mM L-glutamine (Life Technologies), supplemented with 10% fetal bovine serum (MSC-qualified; Life Technologies), and 1x Antibiotic-Antimycotic (Life Technologies). After 7–10 days, once cells started to appear, explants were removed. When cells reached 70–80% confluency, they were dissociated and re-plated. Following passage 0 (P0), in all the subsequent passages, UMSCs were cultured in the same medium as mentioned above, except that Antibiotic-Antimycotic was replaced with 1% penicillin-streptomycin (Life Technologies). Cells were dissociated using TrypLE Express (Life Technologies) and plated at a density of 5000 cells/cm^2^. All experiments were performed with UMSCs between passages 4 and 6. Under standard control conditions, UMSC cultures were incubated at 37°C with 5% CO_2_ and 20% O_2_ [1-3].

For adipocyte, osteocyte, and chondrocyte differentiation, cells were cultured in adipogenic, osteogenic, and chondrogenic differentiation media, respectively. Adipocytes were stained with 0.3% oil red O for 10 minutes at room temperature and counterstained with hematoxylin. The osteogenic differentiation level of cells was assessed by Alizarin red S staining (1%) to detect calcium mineralization. The chondrogenic differentiation level of cells was confirmed by Alcian blue staining for sulfated proteoglycans [4].

**Co-culture of UMSCs and U87 GBM cells**

Transwell chambers with 0.4 μm-pore diameter filters that prevent cell infiltration were employed (including UMSCs, UMSCs/*PD-1*, UMSCs/*miR-124*, UMSC/*miR-124-PD-1* and UMSC/*miR-124-PD-1* EV). UMSCs were plated onto the transwell inserts, while GL-261 or GBM8401 cells were seeded in the lower well of the transwell chambers (with varying GBM:UMSC ratios, including 1:1, 1:3, or 1:5). After 48 hr of co-culture, cells in the lower well of glioblastoma cells were collected for further evaluation.

**TaqMan Real-time RT-PCR.**

Real-time RT-PCR analysis for miR-124-3p, CD4, and CD6 was performed in triplicate using the TaqMan MicroRNA assay kit (Ambion) according to the manufacturer's instructions. The RT reaction contained 10 ng total RNA, 1 mM dNTPs, 50U Multiscribe Reverse Transcriptase, 1.5 µl 10× RT buffer, 0.188 µl RNase inhibitor, and 3 µl 5× TaqMan MicroRNA RT primer in each reaction (15 µl). The RT reaction was conducted under the following conditions: 16°C for 30 min; 42°C for 30 min; 85°C for 5 min, and then held at 4°C. After the RT reaction, the cDNA products from the RT reaction were diluted 15 times. PCR was carried out with 1.33 µl of the diluted products in a 20 µl PCR reaction containing 1 µl of TaqMan MicroRNA Assay and 10 µl of TaqMan Universal PCR Master Mix. Real-time PCR was done in triplicates with iQ-SYBR Green Supermix (Bio-rad, CA, USA) and Icycler Instrument (Bio-rad, CA, USA) using 1 µl diluted cDNA as template in a 20 µl reaction volume. PCR reaction was carried out as following: 95°C for 3 min and 40 cycles of 95°C for 20 s, 55°C for 30 s, and 72°C for 20 s.

**Colony formation assay**

GL261 and GBM8401 cells were transfected with miR-124 or treated with UMSC-derived exosomes overnight. Following treatment, the pre-treated cells were seeded into 6-well plates at a density of 1×10^3^ cells/well. After two weeks, the medium was removed, and the cells were fixed with a mixture of methanol and acetic acid (3:1) for 15 min. Subsequently, they were stained with a 0.3% crystal violet solution for 5 min and air-dried. The colony formation rate was determined using the following formula: Colony formation rate = (number of colonies/number of seeded cells) × 100%.

**Flow cytometry (PD-1 detection)**

To assess the expression of PD-1 and in UMSCs, we initiated the process by collecting cells from parental UMSCs, UMSC/*PD-1*, UMSC/*miR-124* and UMSC/*miR-124-PD-1* for surface PD-1 detection. For surface PD-1 staining, all cell types were gently detached and harvested using Accutase. Subsequently, a count of 1 x 10^6^ cells was performed, and the cells were pelleted with 200 rcf for 5 minutes at 4 degrees Celsius. Pelleted cells were the resuspended with staining buffer (1% BSA in PBS) contained with Alexa Fluor® 647 Mouse anti-Human CD279 (PD-1) (560838, BD, NJ, USA) and incubated for 15 minutes at 4 degrees Celsius, protected from light. To detect the expression of PD-1 on the cells, the stained cells were detected using APC channel of NovoCyte Flow Cytometer Systems (Agilent, CA, USA).

**Cell Cycle Analysis**

GL-261 or GBM8401 cells were seeded into 12-well plates at a density of 2 × 10^5^ cells/well and incubated overnight. In addition, for positive control evaluation, we treated GL-261, U-87 MG, and GBM8401 cells with palbociclib (CDK inhibitor) for 24 hr. After co-culture with UMSCs for 6 hr, cells were collected and fixed with 70% ethanol, incubated overnight at -20°C, washed with PBS, and resuspended in 500 µL propidium iodide (PI) buffer (40 µg/mL PI, 100 µg/mL RNase, and 1% Triton X-100 in PBS) for 30 minutes in the dark at room temperature. Forward Scatter (FSC) and Side Scatter (SSC) parameters were used to gate GBM cells because the size of GBM cells was relatively smaller than that of UMSCs. The subG1, G1, S, and G2-M populations were then evaluated using flow cytometry (CytoFLEX flow cytometer, Beckman Coulter, Inc., Brea, CA, USA).

**Apoptosis Analysis**

Apoptosis was validated using the mitochondria assay, and caspase-3, Annexin V activity analysis and TUNEL assay. Cells were seeded in 12-well plates at a density of 2×10^5^ cells/well and incubated overnight. After co-culture with UMSCs for 48 hr, cells were harvested by centrifugation, washed twice with PBS, and resuspended in 500 µL PBS 1 µL fluorescein isothiocyanate (FITC)-DEVD-FMK, and then incubated for 30 minutes at 37°C and were measured using flow cytometry. For the Annexin V assay and TUNEL assay, the experiments followed the protocols of the Annexin V-FITC Apoptosis Detection Kits and APO-BRDU™ Kit (BD Biosciences, San Jose, CA, USA).

**Invasion, and migration transwell assay.**

Transwell chambers, with or without Matrigel coating, were utilized to measure cell invasion and migration. The 8 μm pore-sized Transwell chambers were placed in 96-well plates and coated with 50 μL Matrigel or left uncoated. The chambers were then incubated at 37°C with 5% CO2 for 1 hour. After co-culture, 2×10^5^ cells were collected and seeded in the apical chamber with serum-free medium. Next, DMEM supplemented with 10% FBS was added to the basolateral chamber, followed by incubation for 24 hours. Sterile cotton swabs were used to remove the Matrigel in the Transwell chamber. The invasive or migrating cells on the bottom of the Transwell were fixed with a mixture of 3:1 methanol and acetic acid, stained with 0.5% crystal violet for 15 minutes, and then counted under a light microscope at a magnification of 100×.

**Wound healing assay**

GL261 and UMSCs cells (2×10^5^) were seeded into 6-well plates with ibidi culture-inserts (cat: 80,241, ibidi GmbH, Gr¨ afelfing, Germany) and allowed to incubate overnight. The two-well insert was then removed, and the migration patterns were observed under a microscope at 0, and 20 h. The gap area was quantified using ImageJ software version 1.50 (National Institutes of Health, Bethesda, MD, USA).

**Magnetic Resonance Imaging (MRI).**

Animal MRI data was acquired using a Brucker 7T and 9.4T PharmaScan scanner. Animals was anaesthetised under ~1.2% isoflurane, and the stereotaxic headpiece and holder consisting of ear and tooth bars will be used to immobilise the head. Physiological conditions including heart rate, arterial pulse extension, oxygen saturation, and rectal temperature was continually monitored and kept within normal ranges throughout the experiment. A volume coil was used for radiofrequency (RF) excitation, and a circular surface coil was used for signal detection. T2 weighted image: Following initial localization scans, a rapid acquisition with relaxation enhancement (RARE) sequence with repetition time (TR)/echo time (TE) = 2650/40 ms, field-of-view (FOV) = 2.56 × 2.56 cm, matrix size = 192 × 192, 12 slices and slice thickness of 1 mm, was performed to acquire anatomical images for registration. Tumor volumes were obtained from the T2 weighted images. Images analysis was mentioned in our previous studies [5].

**Blood biochemical tests.**

All procedures followed the guidelines of the National Centre for the Replacement Refinement and Reduction of Animals in Research of the National Institutes of Health. In general, blood samples were withdrawn from the hearts of mice following an overdose of anesthesia, with a volume of 0.6 mL/kg. The collected sera were separated by centrifugation at 750×g for 15 minutes and stored at –20°C until analyzed. Serum aspartate aminotransferase (AST), alanine aminotransferase (ALT) and gamma glutamyl transpeptidase (γGT) activities were measured to validate the liver function after drug treatment. Creatinine (CREA) was used to measure kidney function after drug treatment.

**Nano-single-photon emission computed tomography/computer tomography (nano-SPECT/CT).**

In addition, for UMSC/*miR-124-PD-1* distribution analysis, mice were injected with 68-Ga and scanned by single-photon emission computed tomography. Mice from the vehicle or imipramine group were scanned by a nano-SPECT/CT scanner (Mediso Ltd., Budapest, Hungary) once per week after treatment. Mice were anesthetized using 1-3% isoflurane and scanned for 10 minutes (CT image operation parameters: tube energy 55 kVp × 145 µA, 360 projections, voxel size 145 × 145 × 145 µM).

**Effect on peripheral blood mononuclear cells co-culture with UMSCs.**

Peripheral blood mononuclear cells (PBMCs, 1×10^5^) were cultured in RPMI medium supplemented with 10% FBS and 3.3 ng/mL hIL-2. To assess the capability of UMSCs to induce T cell proliferation, we initiated an overnight co-culture of gene-modified UMSCs (UMSC/PD-1, UMSC/miR-124, UMSC/miR-124-PD-1) with V450-labeled PBMCs at a 1:3 ratio in 24-well plates for 5 days. In the meantime, anti-CD3/28 beads (ImmunoCult™ Human CD3/CD28 T Cell Activator, STEMCELL, BC, Canada) were used as a positive control. On the fifth day, suspended cells were harvested, and T cell gating was performed through staining with multiple cell markers, CD4 and CD8. Proliferation efficiency in the suspended cells was then quantified using NovoCyte Flow cytometry. Moreover, CD86 expression in PBMCs was also evaluated.

**Supplementary Table 1**. **Reagents used in this study.**

| Reagents | Company | Cat no. or product no. |
| --- | --- | --- |
| MSC NutriStem® XF Basal Medium, without Phenol Red | Biological Industries | 05-202-1A |
| MSC NutriStem® XF Supplement Mix | Biological Industries | 05-201-1U |
| UltraGRO™-Advanced | AventaCell | HPCFDCRL10 |
| Fetal Bovine Sera | Hyclone Laboratories, Inc, Utah, UK | SH30396.02HI |
| DMSO | Sigma-Aldrich, St.Louis, MO, USA | CAS 67-68-5 |
| Total Exosome Isolation Reagent (from cell culture media) | Thermo Fisher | 4478359 |
| Pierce™ BCA Protein Assay Kits | Thermo Fisher | 23225 |
| HBSS (1X) | HyClone | SH30588.01 |
| LumiFlash™ Infinity Chemiluminescent Substrate, HRP System | Visual protein | LF16-500 |

**Supplementary Table 2**. **Flow antibodies.**

| Antibodies | Company | Product no. |
| --- | --- | --- |
| Anti-mouse CD8-FITC | BD pharmingen | 553030 |
| Anti-mouse IFN-γ-PE | BD pharmingen | 554412 |
| Anti-mouse IL-2-APC | BD pharmingen | 554429 |
| Anti-mouse CD4-APC | BD pharmingen | 553051 |
| Anti-mouse CD25-PE | BD pharmingen | 553866 |
| Anti-mouse FOXP3-FITC | BD pharmingen | 560403 |
| Anti-mouse CD206-APC | BD pharmingen | 565250 |
| Anti-mouse CD86-PE | BD pharmingen | 551396 |
| Anti-mouse CD11b-FITC | BD pharmingen | 562793 |
| Anti-mouse CD11c-APC | BD pharmingen | 550261 |
| Anti-mouse CD24-FITC | BD pharmingen | 561777 |
| Anti-mouse MHCII-PE | BD pharmingen | 557000 |
| Anti-mouse CD62L-cy5.5 | BD pharmingen | 560513 |
| Anti-mouse CD4-PE | BD pharmingen | 553049 |
| Anti-mouse CD44-APC | BD pharmingen | 559250 |
| Anti-mouse CD45-FITC | BD pharmingen | 553080 |
| Anti-mouse Gr-1-PE | BD pharmingen | 553128 |

**Supplementary Table 3.** **Primary antibodies that used in this study.**

| Antibodies | Company | Product no. |
| --- | --- | --- |
| STAT3 (Tyr705) | Cell Signaling Technology | #9145 |
| STAT3 | Cell Signaling Technology | #9139 |
| NF-κB (Ser 536) | Cell Signaling Technology | #3033 |
| NF-κB | Cell Signaling Technology | #8242 |
| Cyclin D1 | Invitrogen | MA5-16356 |
| CDK4(D9G3E) | Cell Signaling Technology | #12790 |
| CDK6(CDS83) | Cell Signaling Technology | #3136 |
| VEGF | abcam | ab1316 |
| UPA | Elabscience | E-AB-60664 |
| MMP-9 | Invitrogen | PA5-13199 |
| GAPDH | Cell Signaling Technology | #5174 |
| CD9 | Merck Millipore | #CBL162 |
| CD63 | System Biosciences | EXOAB-CD63A-1 |
| CD81 (D3N2D) | Cell Signaling Technology | #56039 |
| PD-1 (PDCD1) | Origene | UM800091 |
| β-actin | Elabscience | E-AB-20058 |

**Supplementary Table 4**. Mean tumor growth time, delay time, and inhibition rate in GL261 tumor bearing mice after treatment with different condition are showed.

| **Treatment** | **MTGT (day)*** | **MTGDT (day)^#^** | **MGIR^$^** |
| --- | --- | --- | --- |
| **Vehicle** | 10.45 | N.A. | N.A. |
| **UMSCs** | 12.63 | 2.18 | 1.21 |
| **UM-miR124** | 32.64 | 22.19 | 3.12 |
| **UM-PD-1** | 22.55 | 12.10 | 2.16 |
| **UM-miR124-PD-1** | 1024.7 | 1014.24 | 98.02 |
| **UM-miR124-PD-1 EV** | 170.72 | 160.26 | 16.33 |

Notes: N.A.: not available. ^*^Mean tumor growth time (MTGT): The expected timeframe when the tumor volume reaches 150 mm^3^. ^#^Mean tumor growth delay time (MTGDT): The disparity between the MTGT of the treated group and that of the vehicle group. ^&^Mean growth inhibition rate (MGIR): The MTGT of the treated group divided by the mean tumor growth time of the vehicle group.

**Supplementary Table 5**. Mean tumor growth inhibition rate and combination index in GL261 tumor bearing mice after treatment with UM-miR124, UM-PD-1, the combination of UM-miR124-PD-1, or the derived exosomes UM-miR124-PD-1 EV.

(A)

| **Xenografts** | **UM-miR124** |  | **UM-PD-1** |  | **UM-miR124-PD-1** | | **Index**^★^ |
| --- | --- | --- | --- | --- | --- | --- | --- |
|  | **MGI**^*^ |  | **MGI**^*^ |  | **Expected**^$^ | **Observed**^#^ |  |
| GL261 | 0.67 |  | 0.52 |  | 0.63 | 0.98 | 0.05 |

(B)

| **Xenografts** | **UM-miR124** |  | **UM-PD-1** |  | **UM-miR124-PD-1 EV** | | **Index**^★^ |
| --- | --- | --- | --- | --- | --- | --- | --- |
|  | **MGI**^*^ |  | **MGI**^*^ |  | **Expected**^$^ | **Observed**^#^ |  |
| GL261 | 0.67 |  | 0.52 |  | 0.53 | 0.88 | 0.26 |

Notes: ^*^Mean growth inhibitory rate (MGI): the 14th day’s mean tumor volume ratio of the treated group/the 14th day’s mean tumor volume ratio of the control group. ^$^Expected growth inhibitory rate: growth inhibition rate of UM-miR124 × growth inhibition rate of UM-PD-1. ^#^Observed growth inhibitory rate: growth inhibition rate of UM-miR124-PD-1. ^★^Index value was calculated by (1- MGI of combination)/(1-Expected growth inhibitory rate). An index <1.0 indicates a synergistic effect.

**Supplementary Table 6**. The serum level of gamma glutamyl transpeptidase (γGT) and creatinine (CREA) from GL261 tumor bearing mice is displayed.

| **Treatment** | **γGT (U/L)** | **CREA** |
| --- | --- | --- |
| **0.1% Vehicle #1** | <3 | 0.38 |
| **0.1% Vehicle #2** | <3 | 0.39 |
| **0.1% Vehicle #3** | <3 | 0.38 |
| **UMSCs #1** | <3 | 0.24 |
| **UMSCs #2** | <3 | 0.22 |
| **UMSCs #3** | <3 | 0.26 |
| **UM-miR124 #1** | <3 | 0.28 |
| **UM-miR124 #2** | <3 | 0.27 |
| **UM-miR124 #3** | <3 | 0.29 |
| **UM-PD-1 #1** | <3 | 0.38 |
| **UM-PD-1 #2** | <3 | 0.35 |
| **UM-PD-1 #3** | <3 | 0.41 |
| **UM-miR124-PD-1 #1** | <3 | 0.32 |
| **UM-miR124-PD-1 #2** | <3 | 0.30 |
| **UM-miR124-PD-1 #3** | <3 | 0.34 |
| **UM-miR124-PD-1 EV #1** | <3 | 0.22 |
| **UM-miR124-PD-1 EV #2** | <3 | 0.20 |
| **UM-miR124-PD-1 EV #3** | <3 | 0.24 |

**Supplementary figures**

**
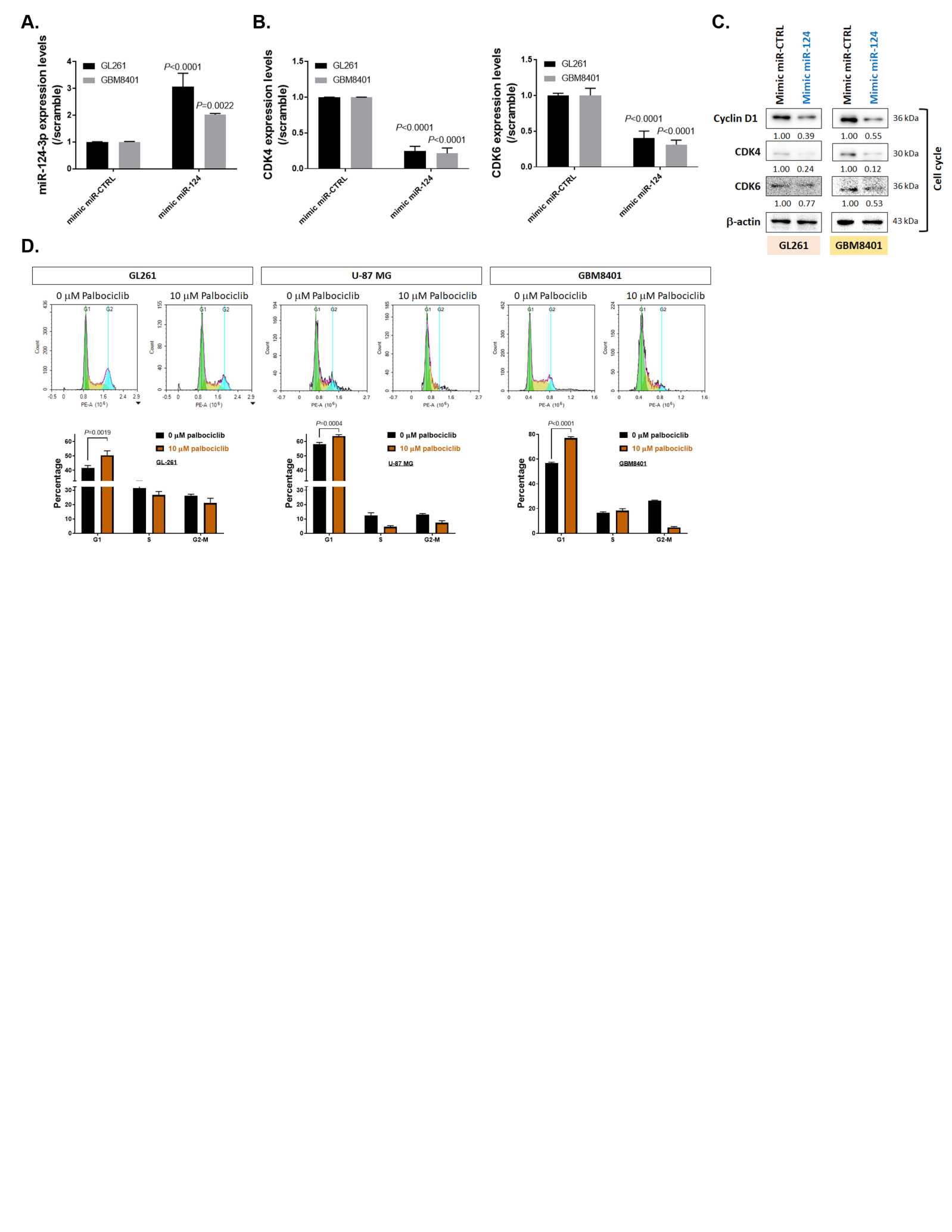
**

**Supplementary figure 1.** The (A) miR-124-3p expression level, (B) CDK4 and CDK6 mRNA expression level, and (C) CDK4 and CDK6 protein expression level after transfecting mimic miRNA-124 in GL261 and GBM8401 cells. (D) Cell cycle analysis of CDK inhibitor (palbociclib) in GL261, U-87 MG and GBM8401 cells.

**
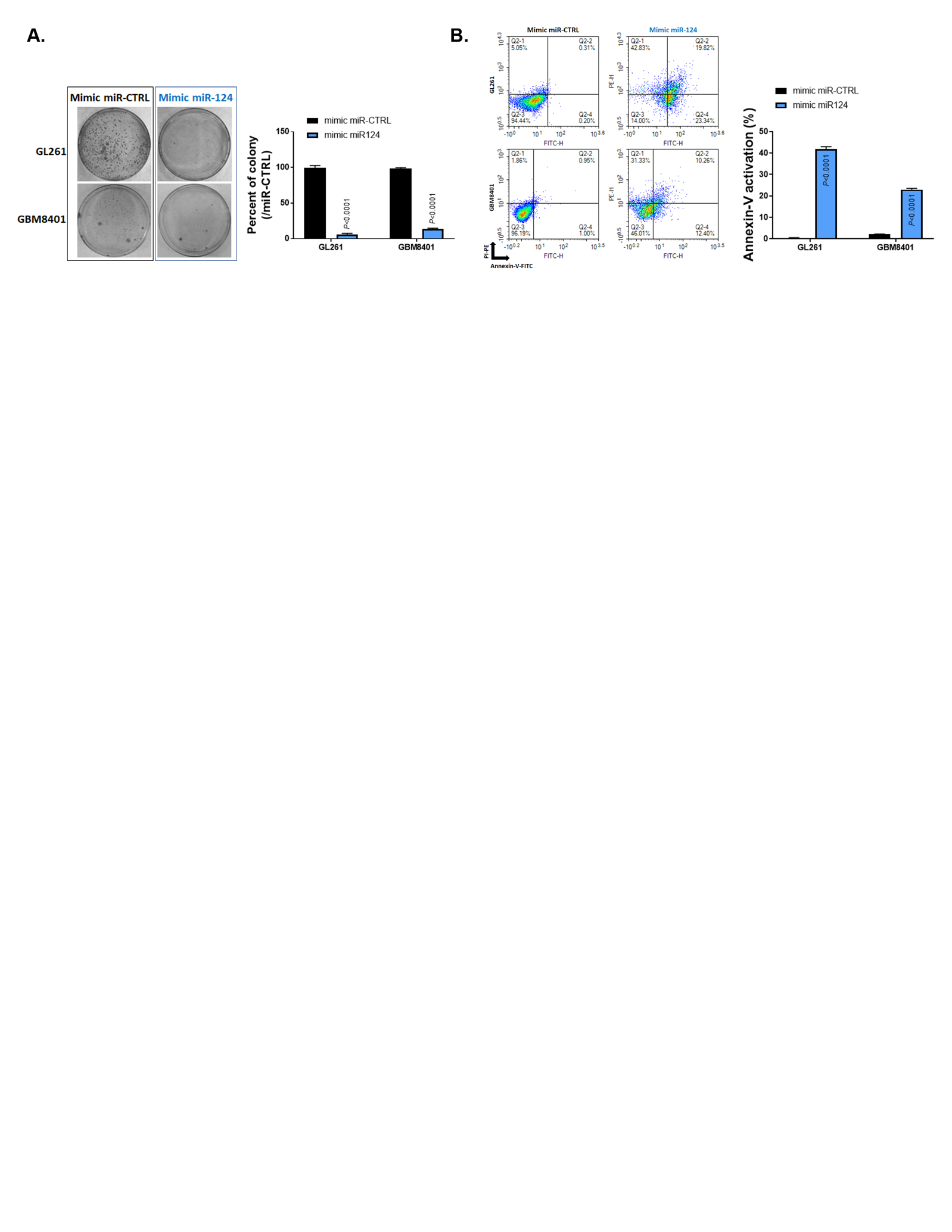
**

**Supplementary figure 2.** The (A) colony formation assay and (B) annexin-v/PI staining after transfecting mimic miRNA-124 in GL261 and GBM8401 cells.


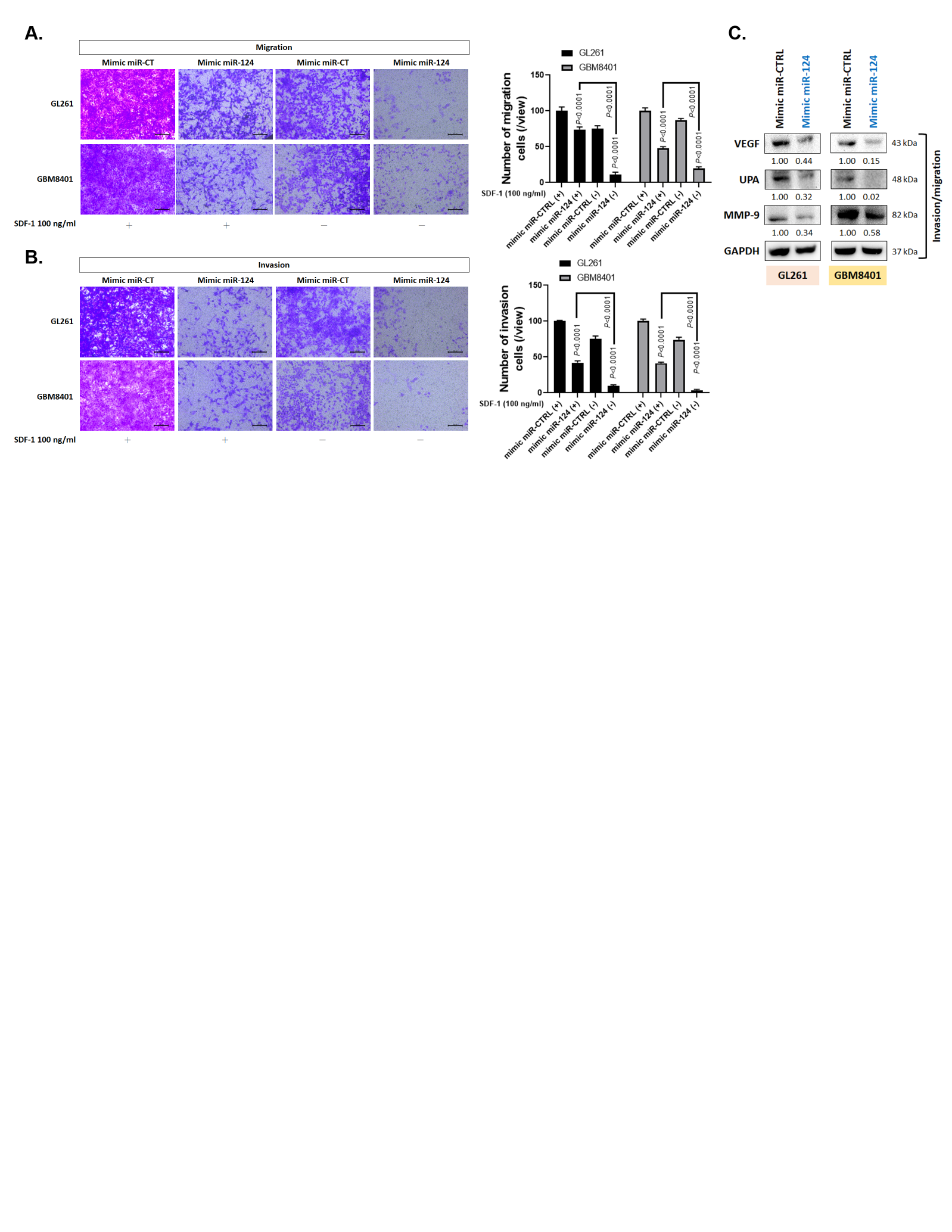


**Supplementary figure 3.** The (A) migration and (B) invasion assay after transfecting mimic miRNA-124 in GL261 and GBM8401 cells. (C) The VEGF, UPA and MMP-9 protein expression level after transfecting mimic miRNA-124 in GL261 and GBM8401 cells.


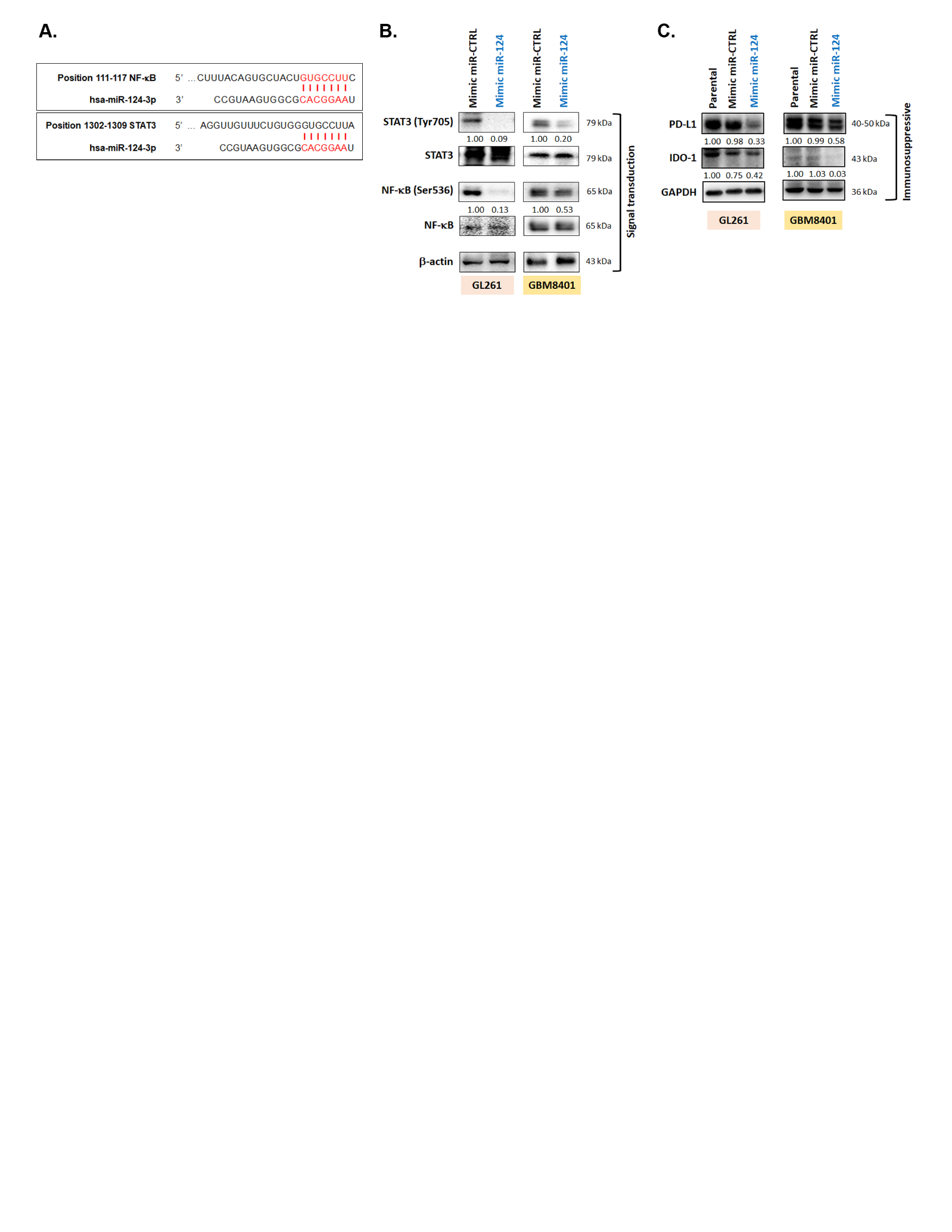


**Supplementary figure 4.** (A) The binding site of miR-124 on STAT3 and NF-κB assayed by TargetScan predicts tools. (B) Expression levels of potential upstream factors STAT3 and NF-κB in miR-124 mimic GL261 and GBM8401 cells assayed by Western blotting. (C) Expression levels of immunosuppressive factors, IDO and PD-L1 in miR-124 mimic GL261 and GBM8401 cells assayed by Western blotting.


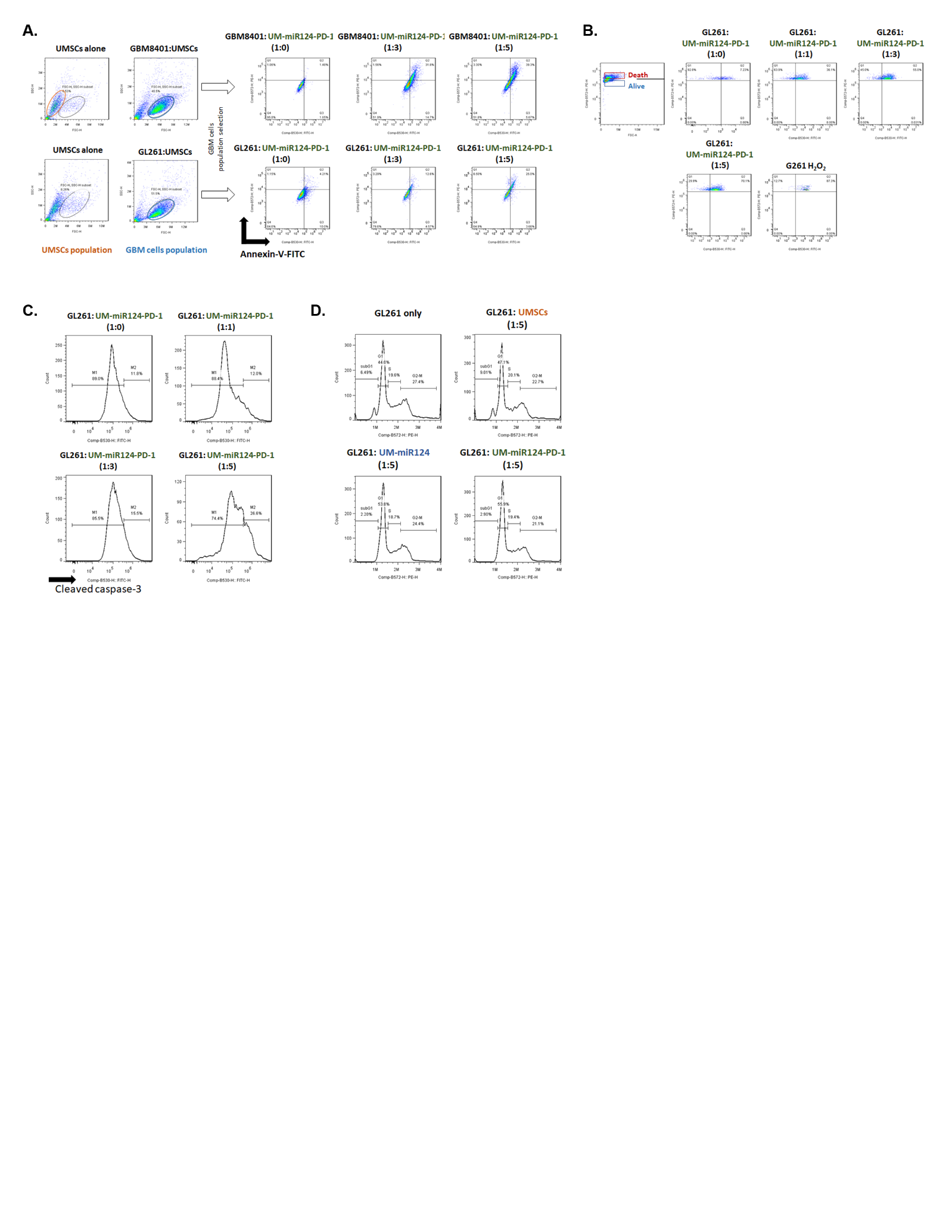


**Supplementary figure 5.** The expression pattern of (A) Annexin-V staining, (B) BrdU staining, (C) cleaved-caspase-3 staining and (D) cell cycle after GL261 or GBM8401 cells co-culture with different ratio UMSC/*miR-124-PD1*.


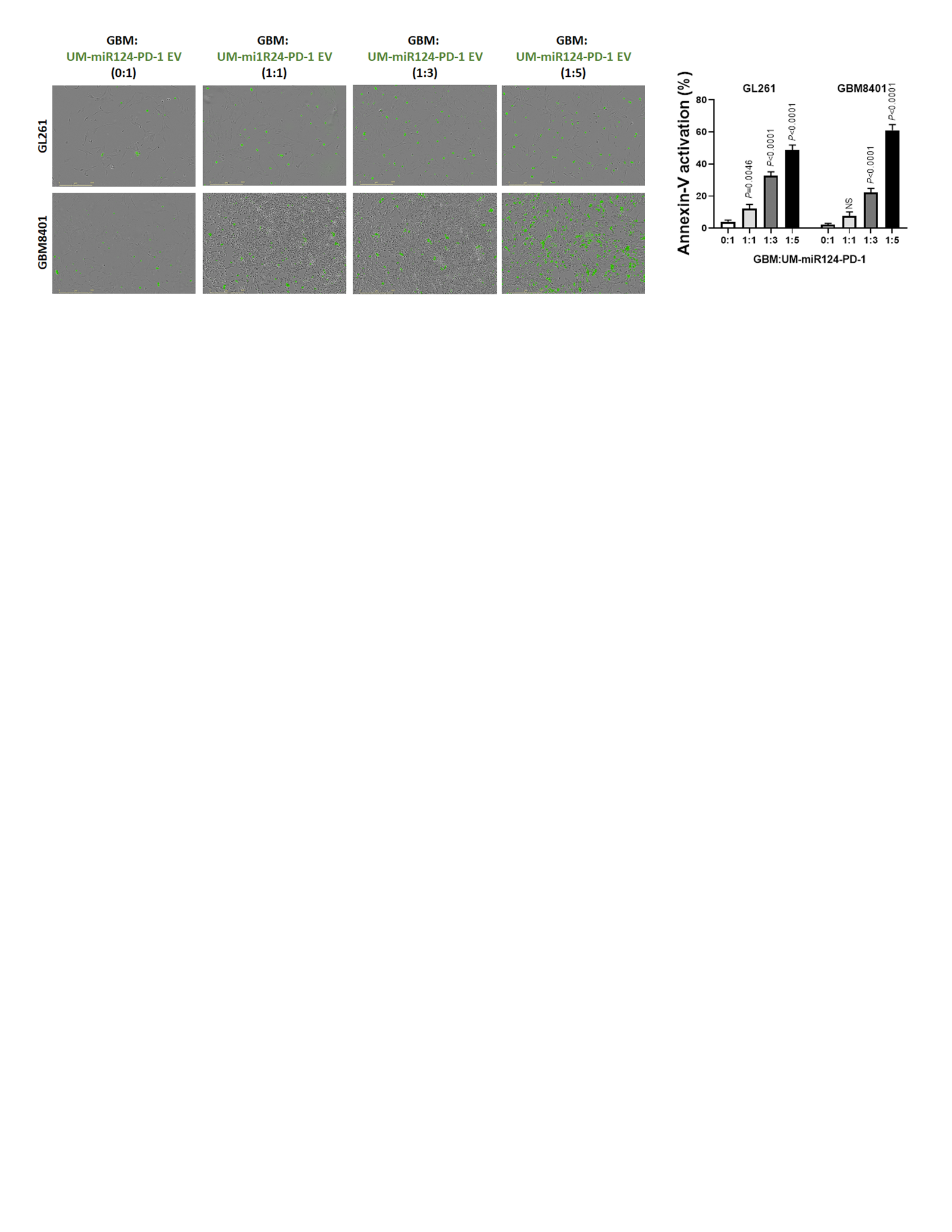


**Supplementary figure 6.** The expression of Annexin-V staining after GL261 or GBM8401 cells co-culture with different ratio UMSC/*miR-124-PD1* derived exosome assayed by IncuCyte S3 system

**
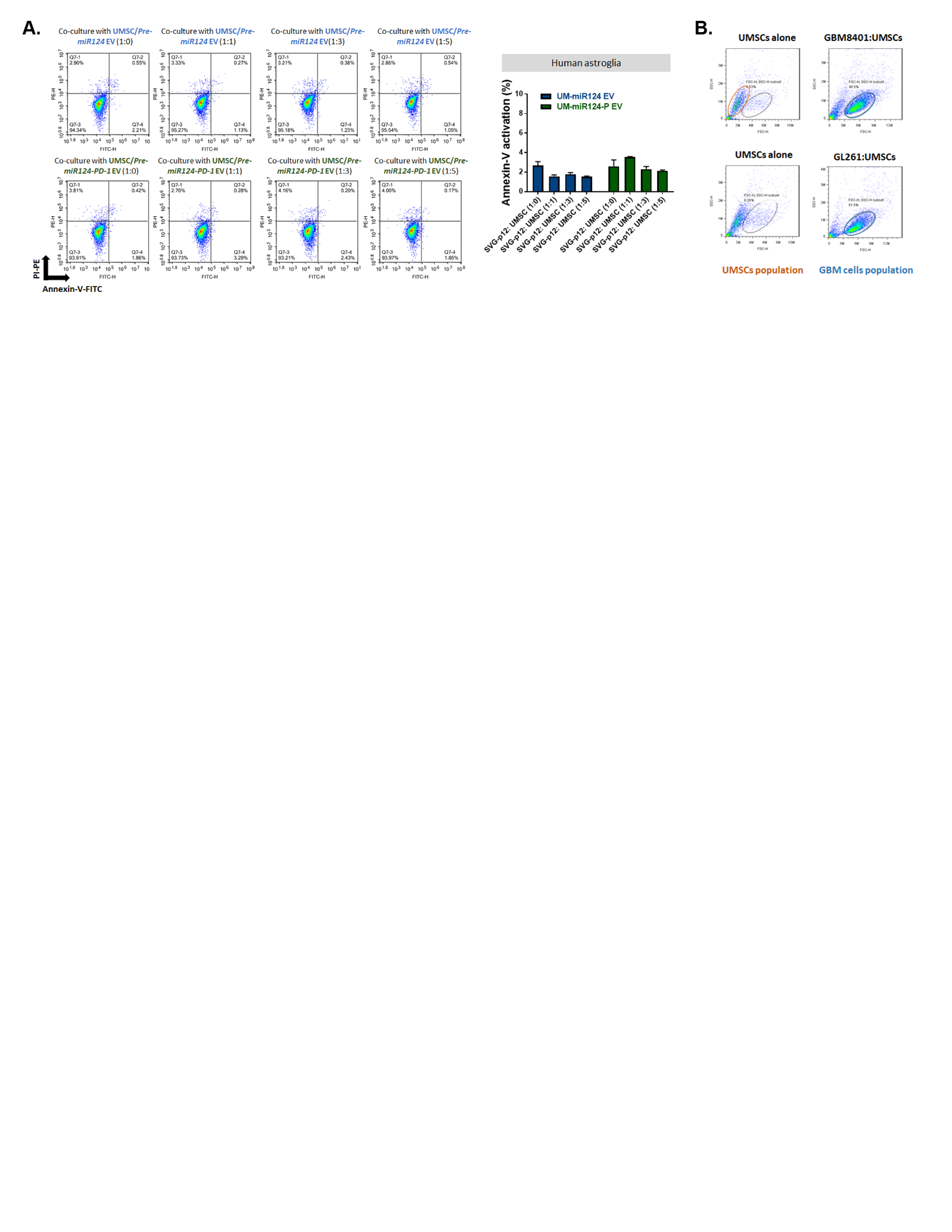
**

**Supplementary figure 7.** (A) The expression pattern of Annexin-V after normal glia cells SVG-p12 co-culture with UMSC/*miR-124* and its exosome. (B) The gate area of GBM8401 or GL261 cells after co-culture with UMSCs from Figure 3L.

**
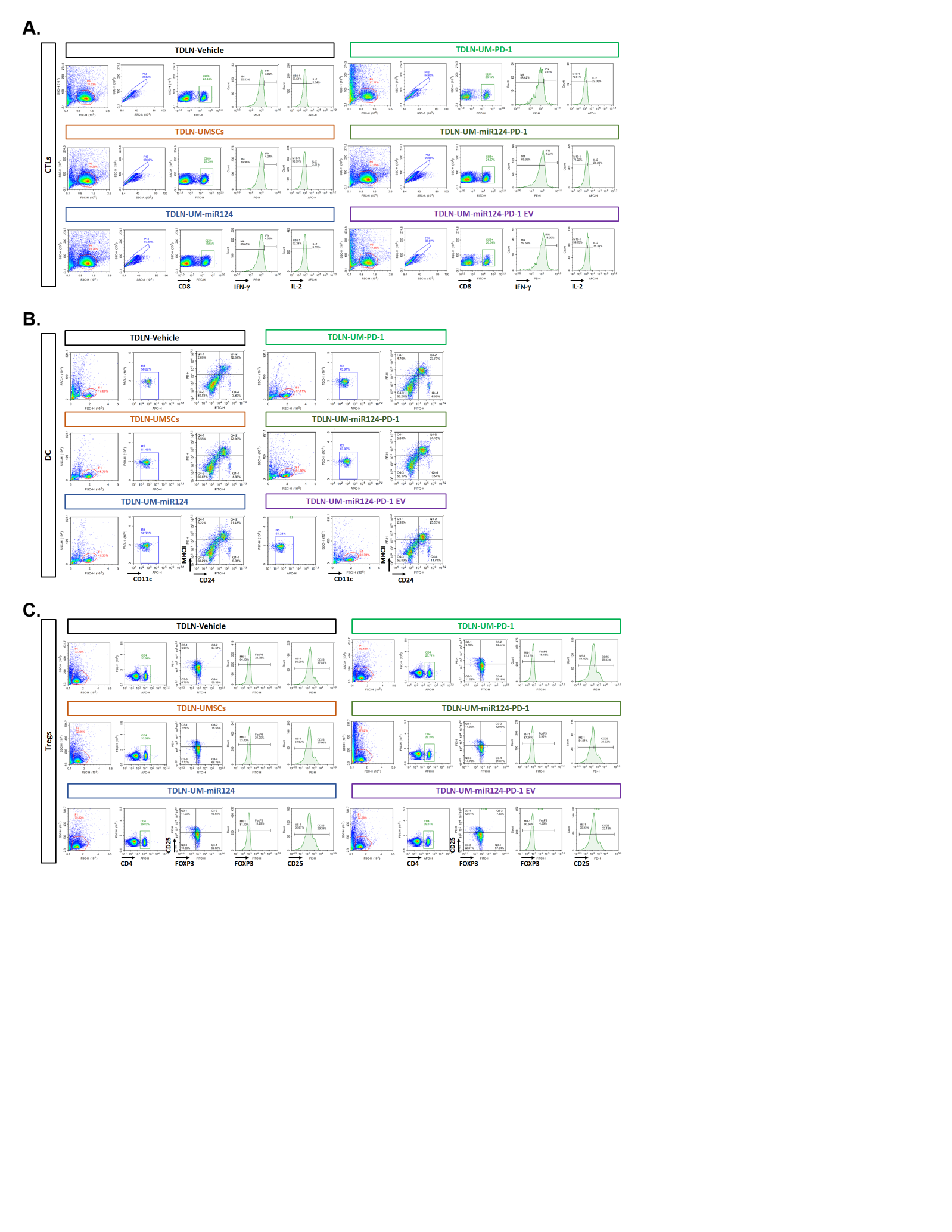
**

**Supplementary figure 8.** The gated pattern of (A) cytotoxic T lymphocytes (CTLs), (B) dendritic cells (DC) and (C) regulatory T cells (Tregs) from tumor-draining lymph nodes (TDLNs) after different treatments.

**
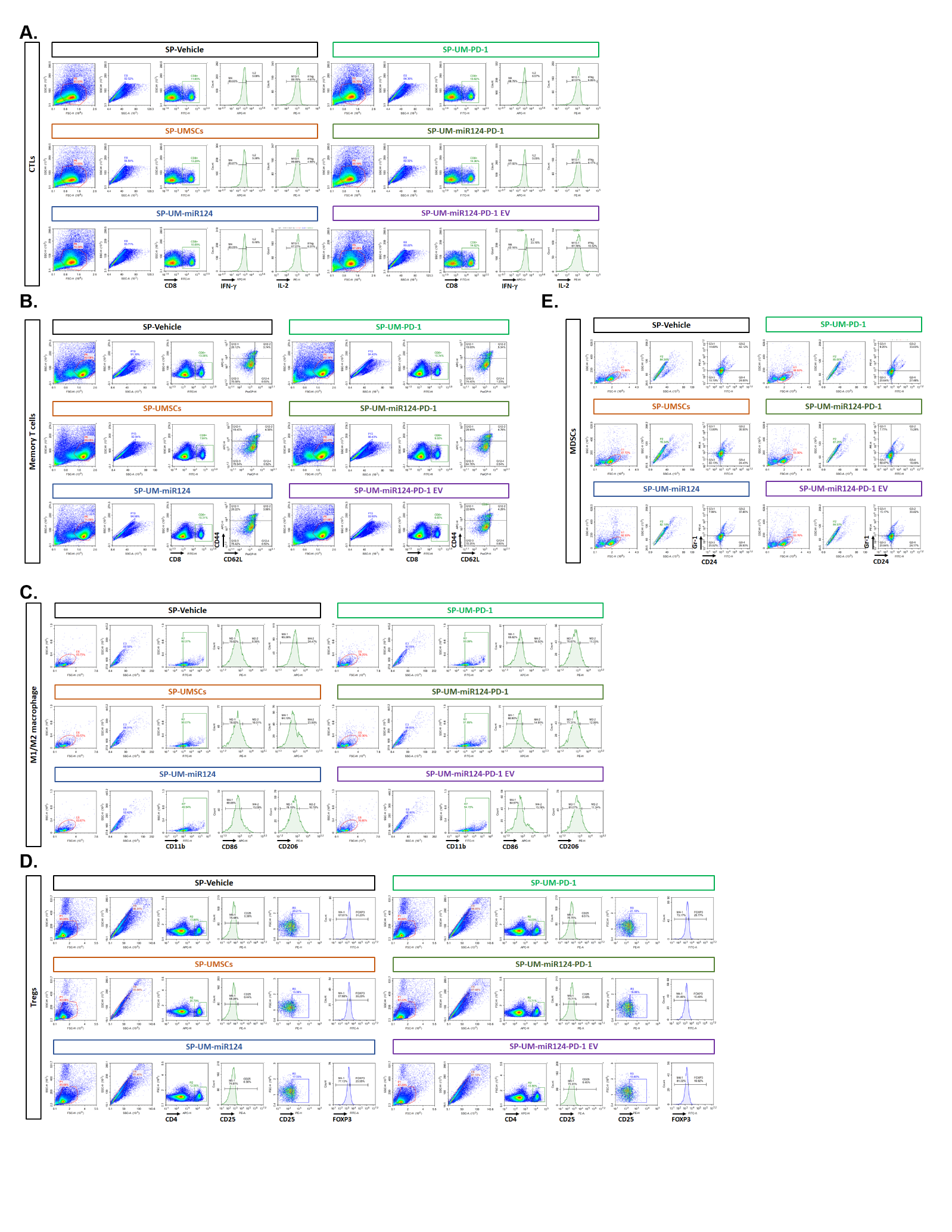
**

**Supplementary figure 9.** The gated pattern of (A) CTLs, (B) memory T cells, (C) M1/M2 macrophages, and (D) Tregs from spleen after different treatments.

**
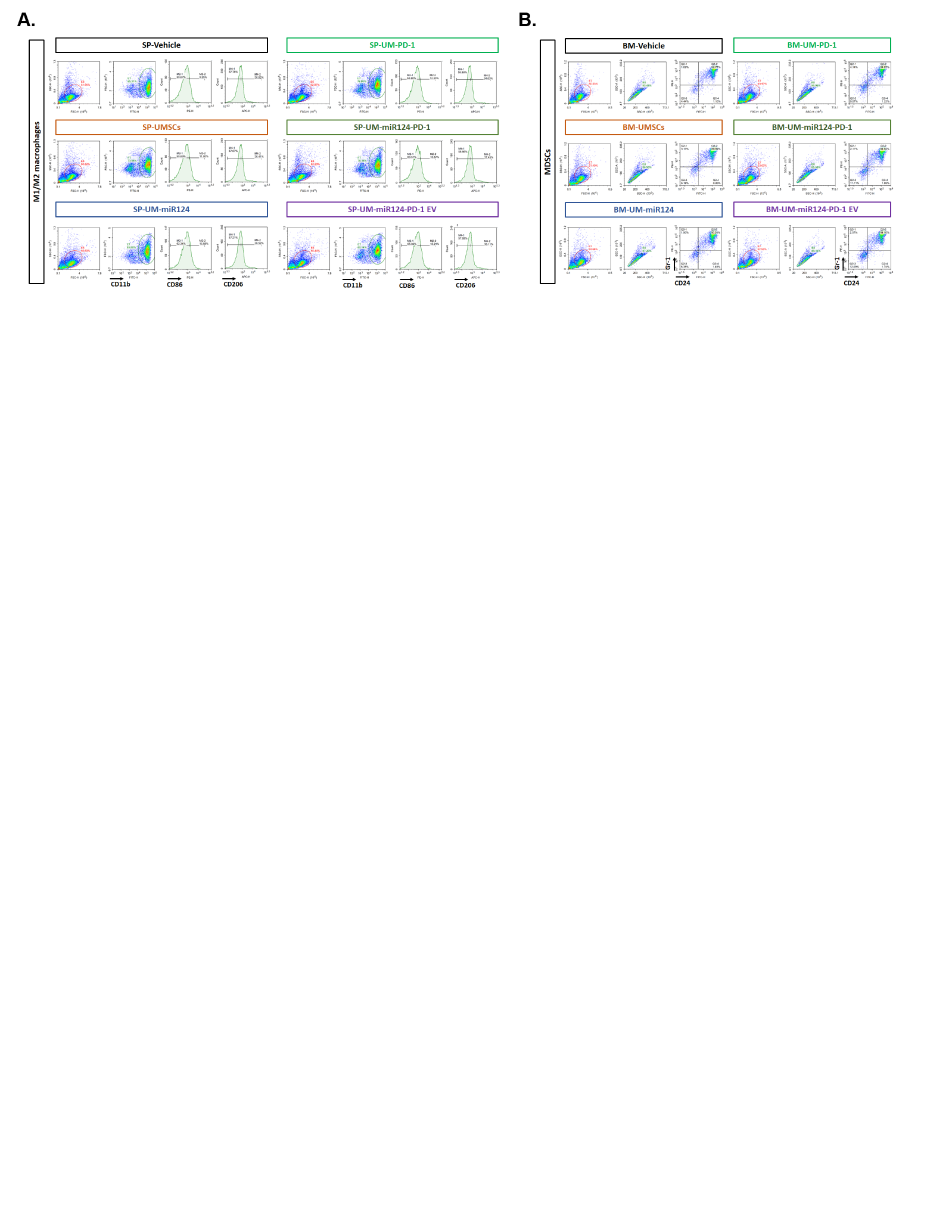
**

**Supplementary figure 10.** The gated pattern of (A) M1/M2 macrophages, and (B) myeloid-derived suppressor cells (MDSCs) from spleen and bone marrow after different treatments.

**
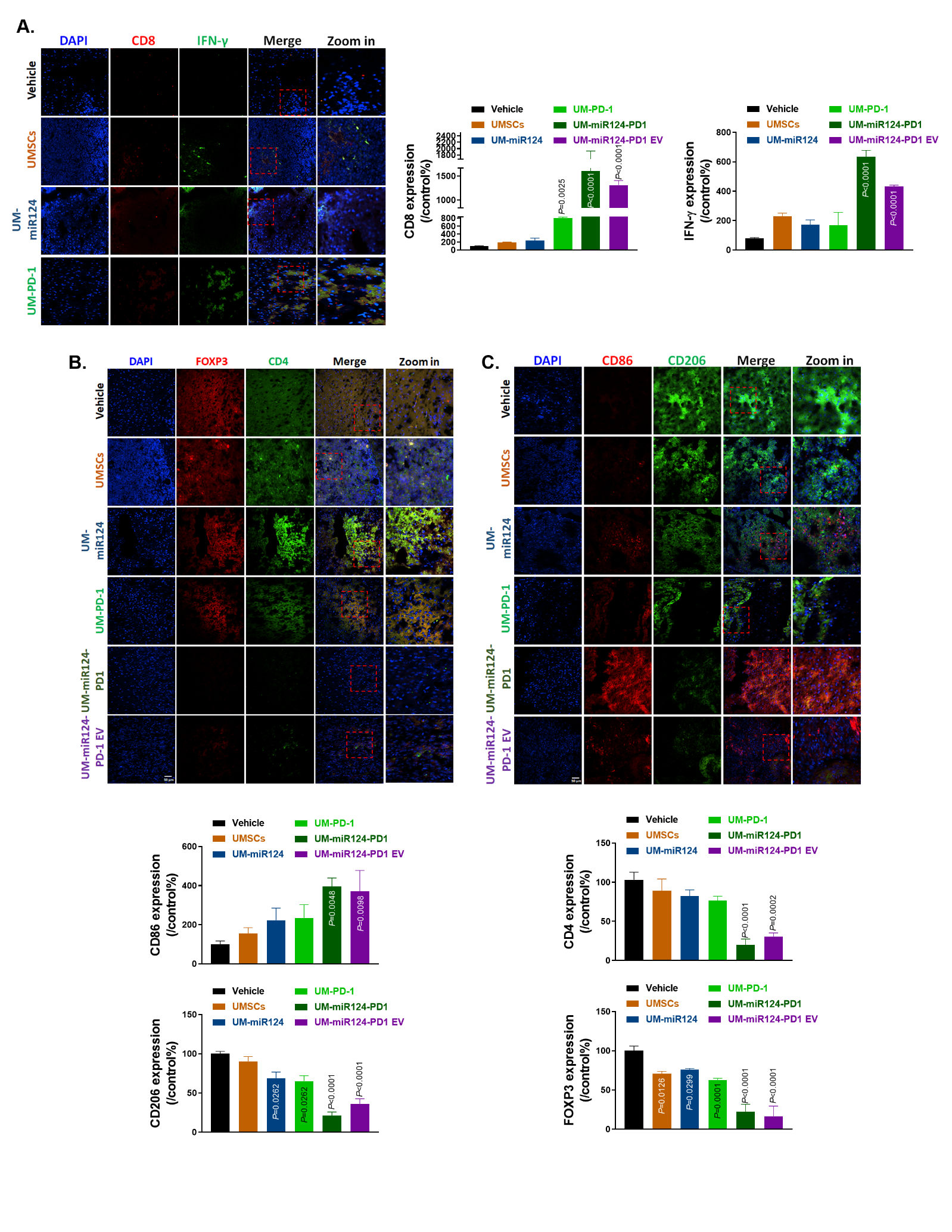
**

**Supplementary figure 11.** The IF staining of tumor tissue with (A) CTLs markers, (B) Tregs markers and (C) M1/M2 macrophages markers.

**
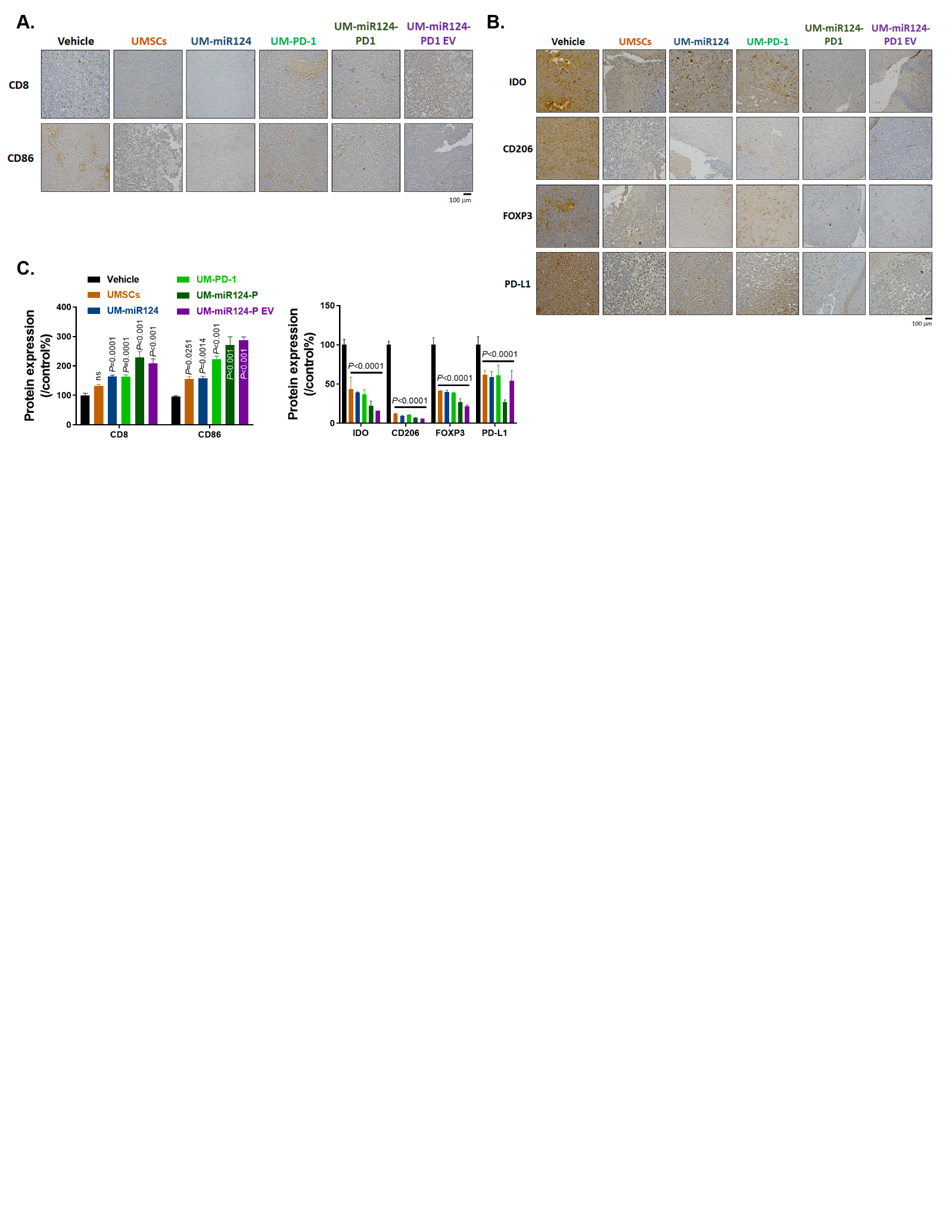
**

**Supplementary figure 12.** The IHC staining of tumor tissue with (A) immunosupportive factors and (B) immunosuppressive factors.

**References**

1. A. Can, S. Karahuseyinoglu, Concise review: human umbilical cord stroma with regard to the source of fetus-derived stem cells, Stem Cells 25(11) (2007) 2886-95.
2. I. Himal, U. Goyal, M. Ta, Evaluating Wharton's Jelly-Derived Mesenchymal Stem Cell's Survival, Migration, and Expression of Wound Repair Markers under Conditions of Ischemia-Like Stress, Stem Cells Int 2017 (2017) 5259849.
3. S.L. Lin, W. Lee, S.P. Liu, Y.W. Chang, L.B. Jeng, W.C. Shyu, Novel Programmed Death Ligand 1-AKT-engineered Mesenchymal Stem Cells Promote Neuroplasticity to Target Stroke Therapy, Mol Neurobiol 61(7) (2024) 3819-3835.
4. U. Nöth, A.M. Osyczka, R. Tuli, N.J. Hickok, K.G. Danielson, R.S. Tuan, Multilineage mesenchymal differentiation potential of human trabecular bone-derived cells, J Orthop Res 20(5) (2002) 1060-9.
5. H.S. Huang, I.T. Chiang, B. Lawal, Y.S. Weng, L.B. Jeng, Y.C. Kuo, Y.C. Liu, F.T. Hsu, A Novel Isotope-labeled Small Molecule Probe CC12 for Anti-glioma via Suppressing LYN-mediated Progression and Activating Apoptosis Pathways, Int J Biol Sci 19(10) (2023) 3209-3225.

**MRI Raw data**

**MRI data of each vehicle group of mice (M1, M5, M8, M10, M11)**


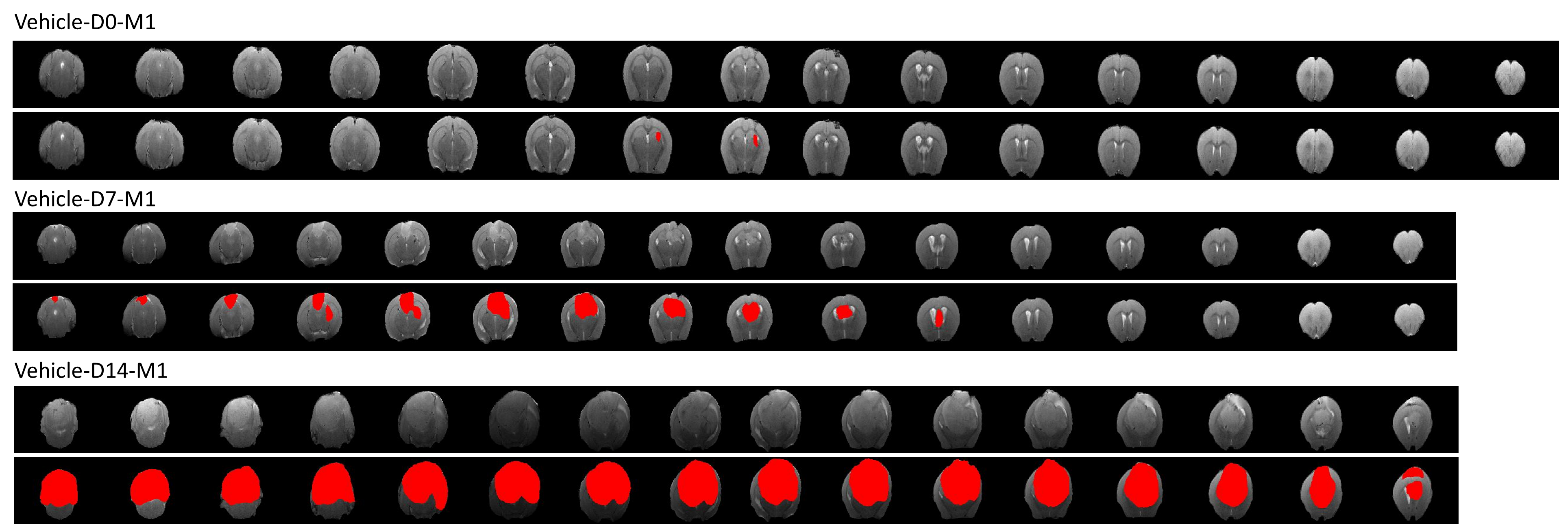


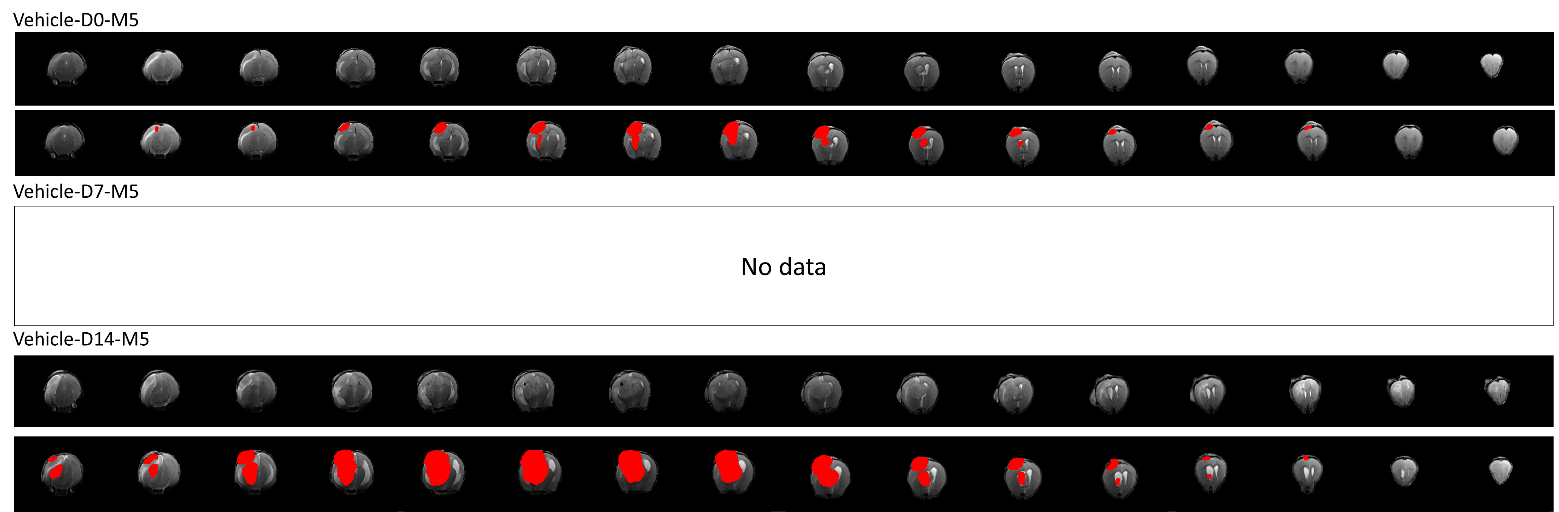


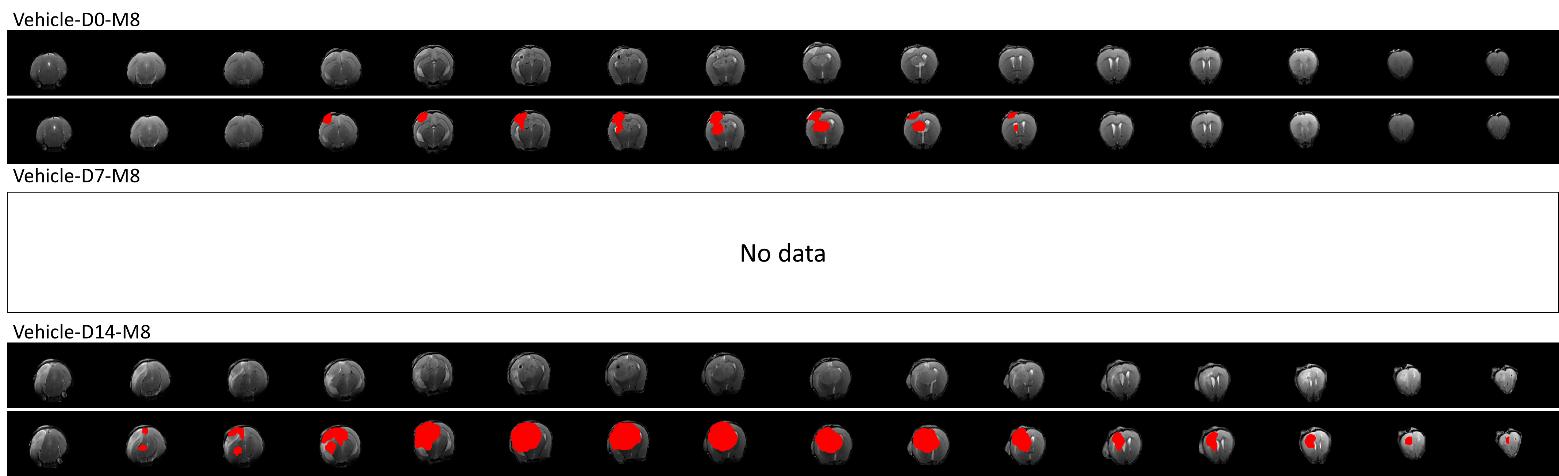


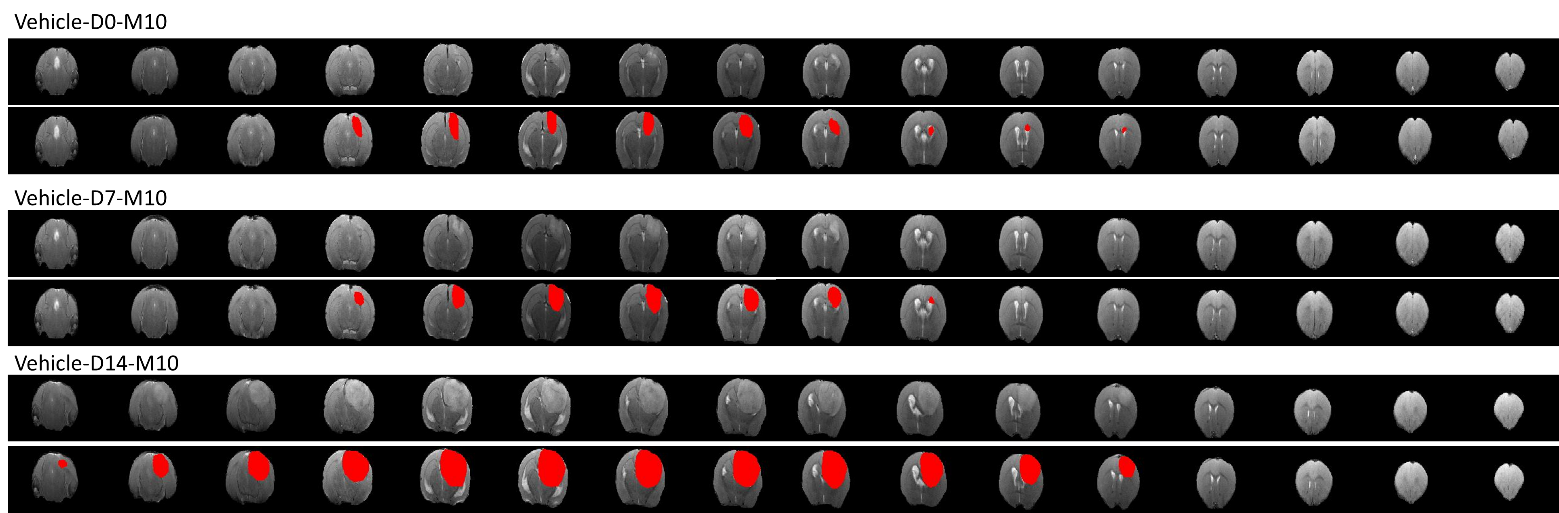


**MRI data of each vehicle group of mice (M1, M5, M8, M10, M11)**
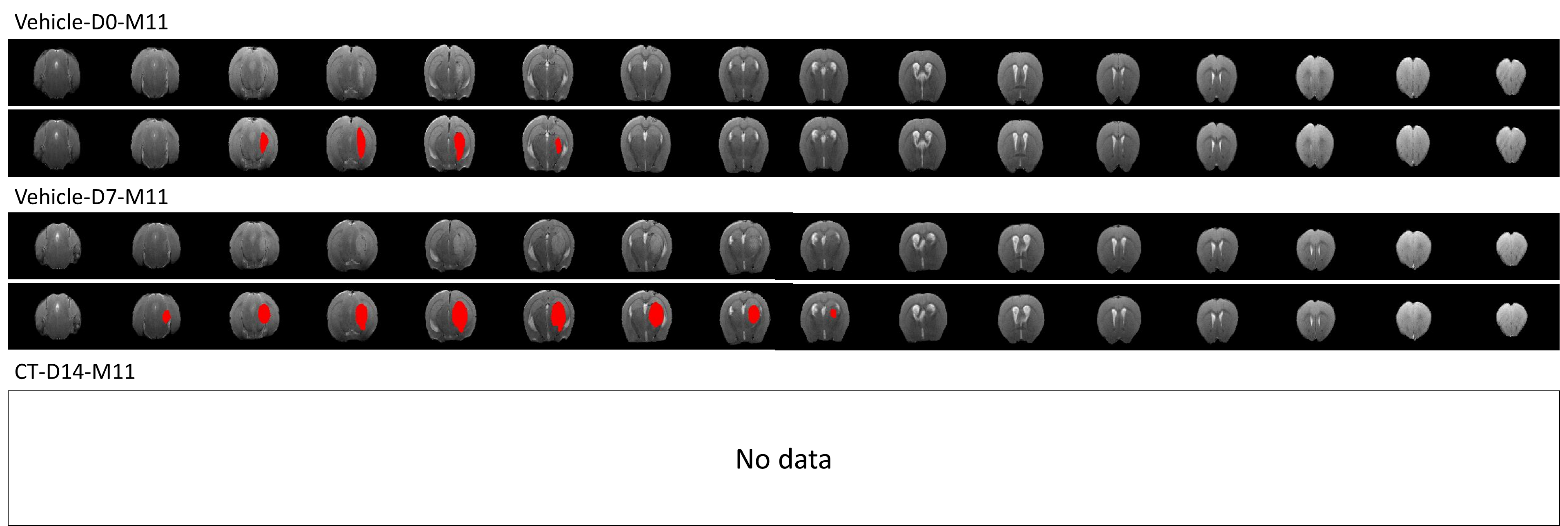


**MRI data of each UMSCs group of mice (M6, M7, M9, M15)**


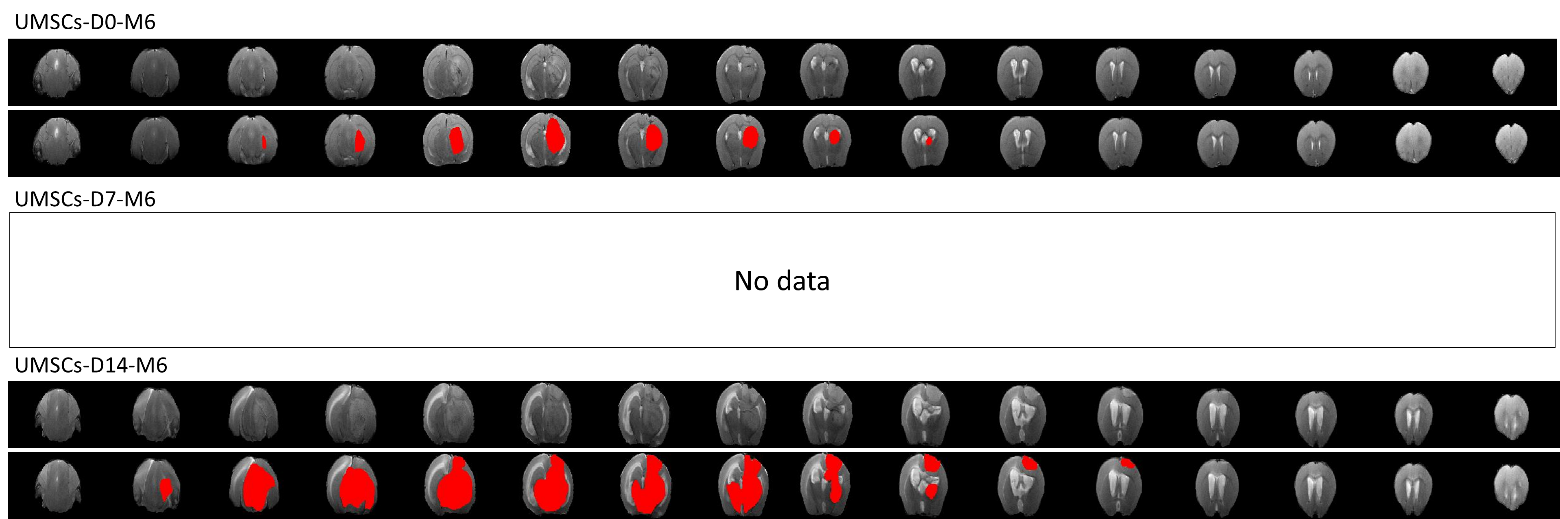


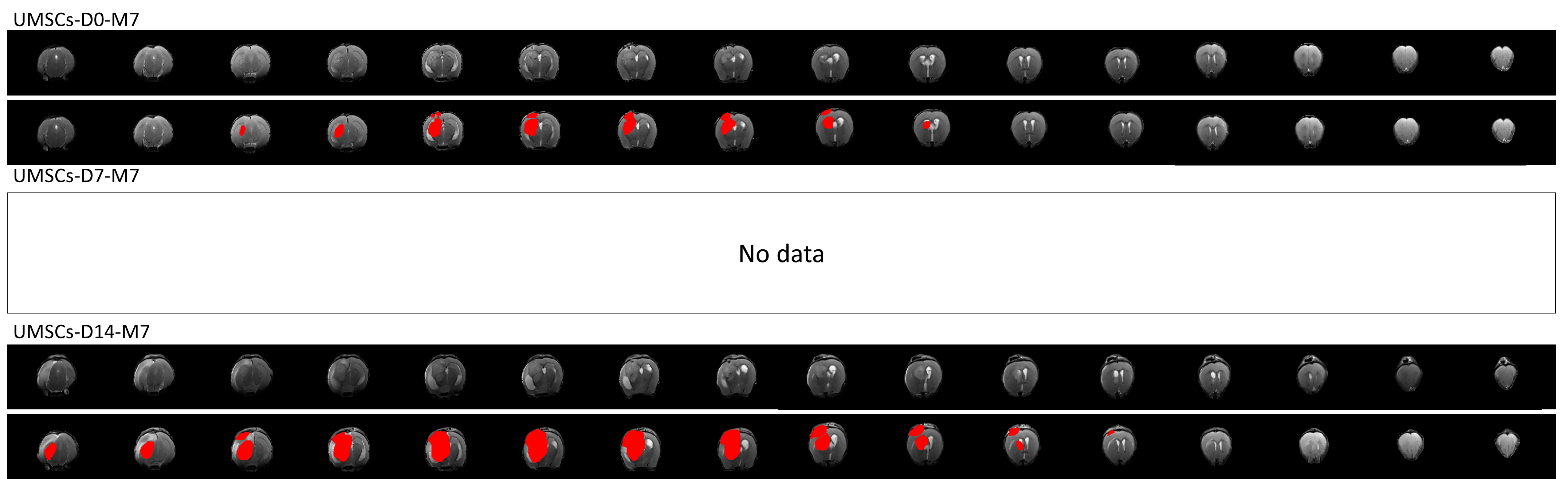


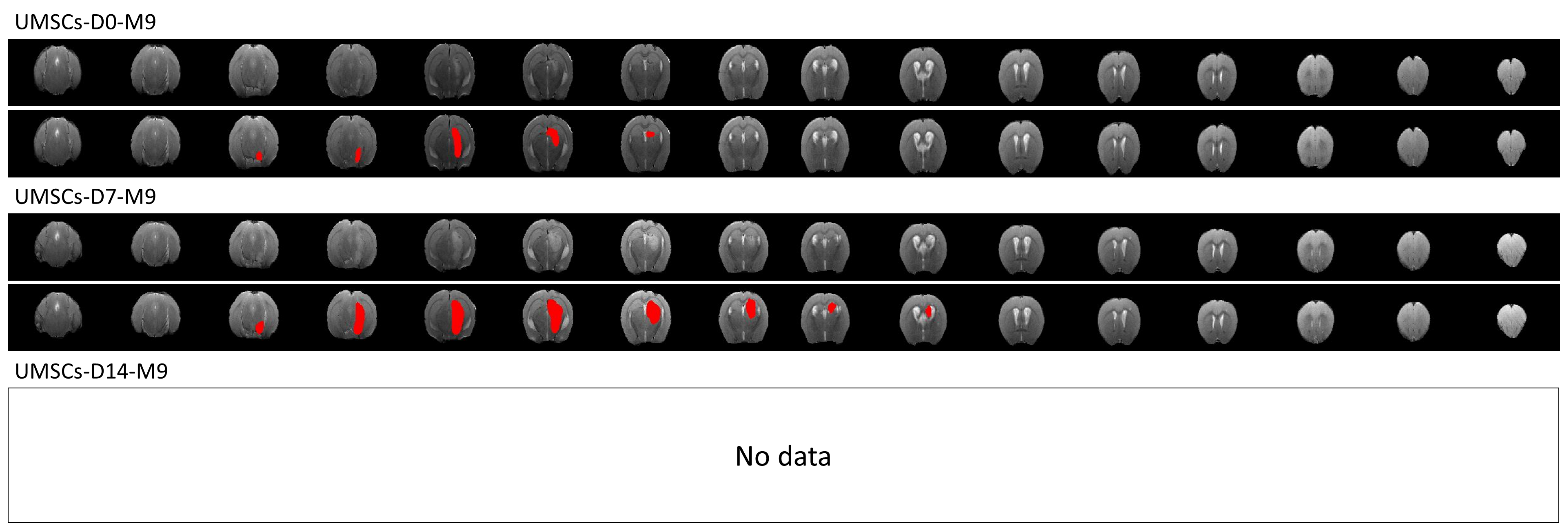


**MRI data of each UMSCs group of mice (M6, M7, M9, M15)**


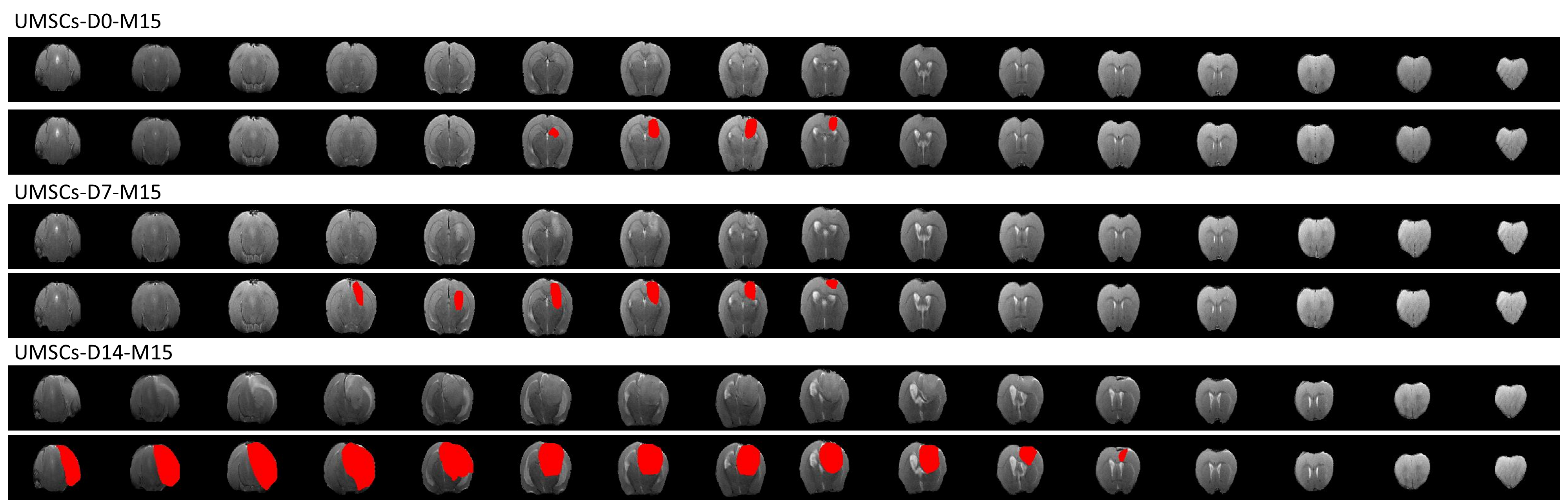


**MRI data of each UM-miR124 group of mice (M4, M12, M13, M14)**


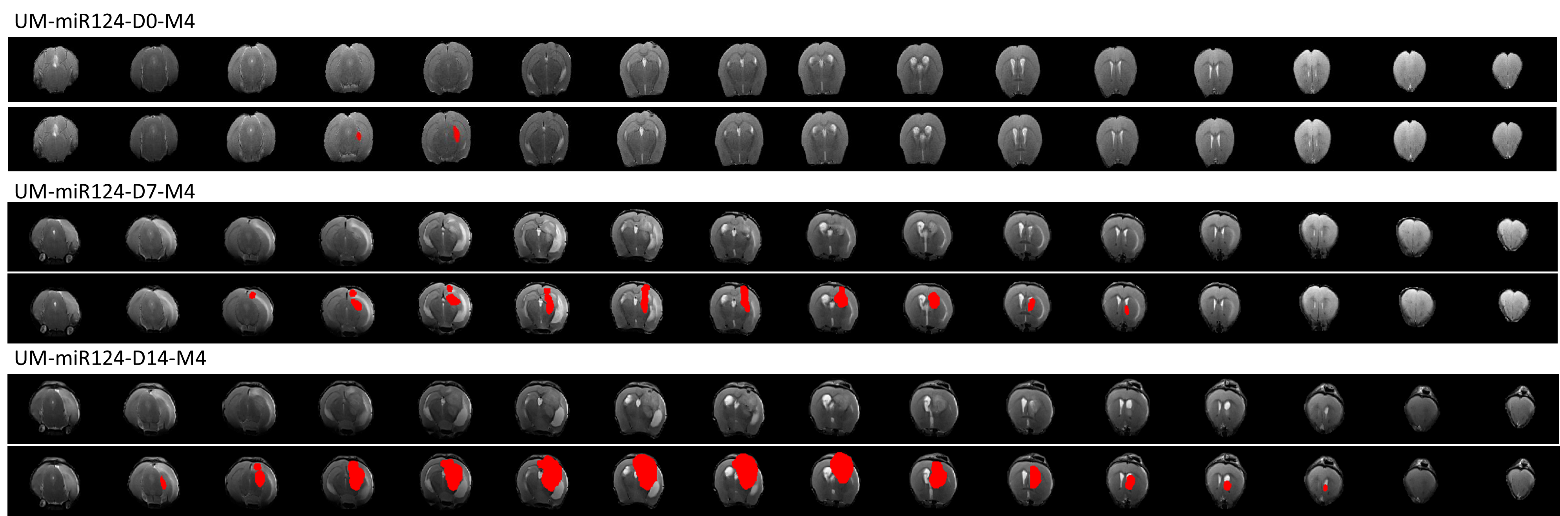


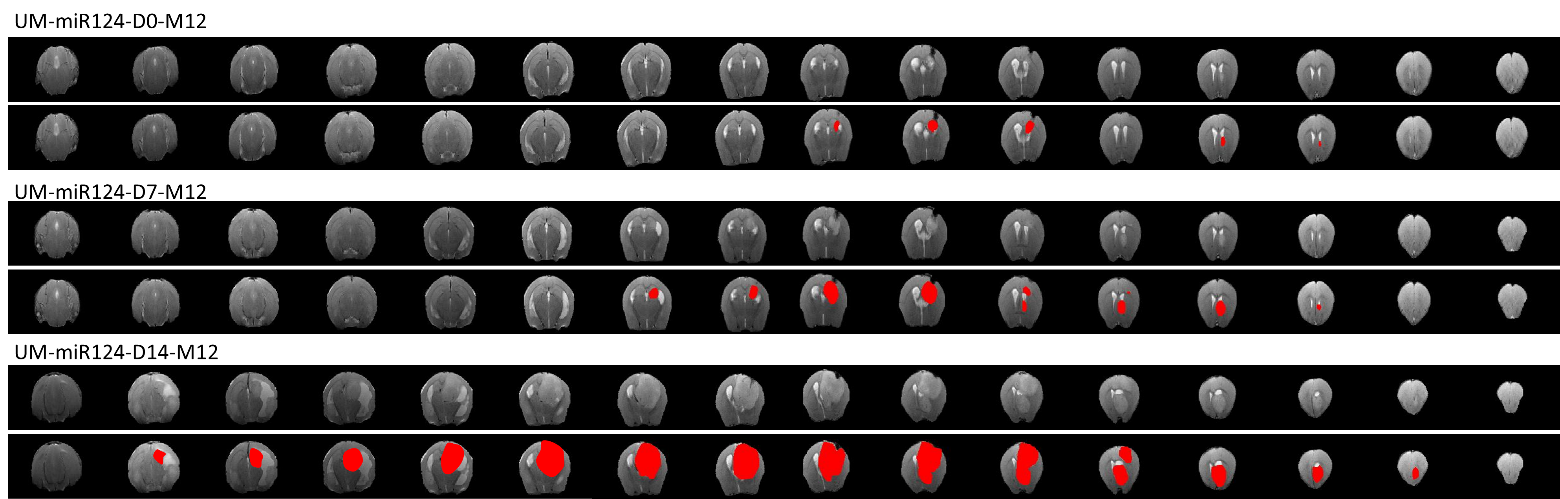


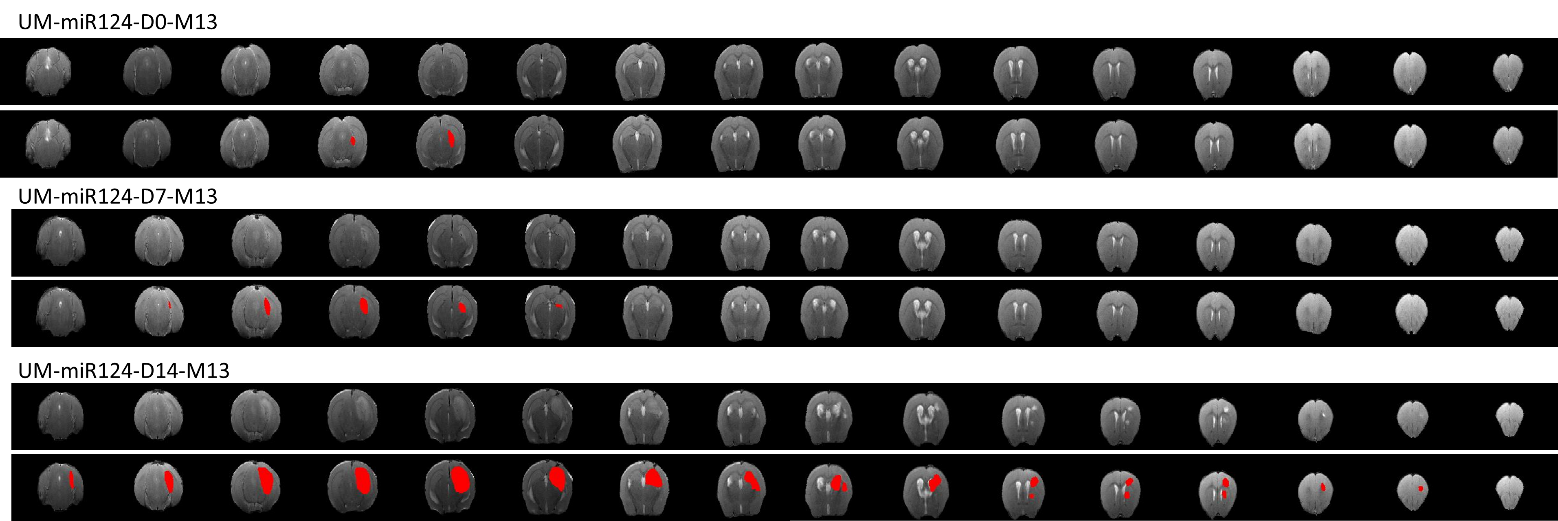


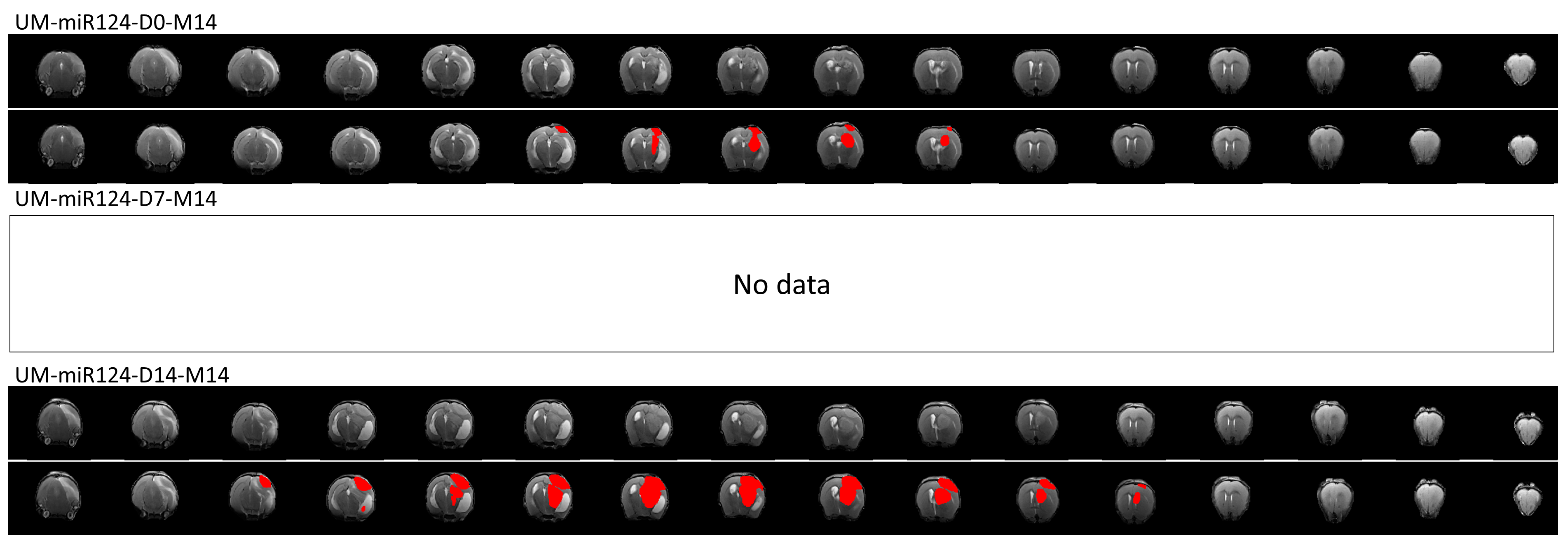


**MRI data of each UM-P group of mice (M2, M3, M16, M18, M20)**


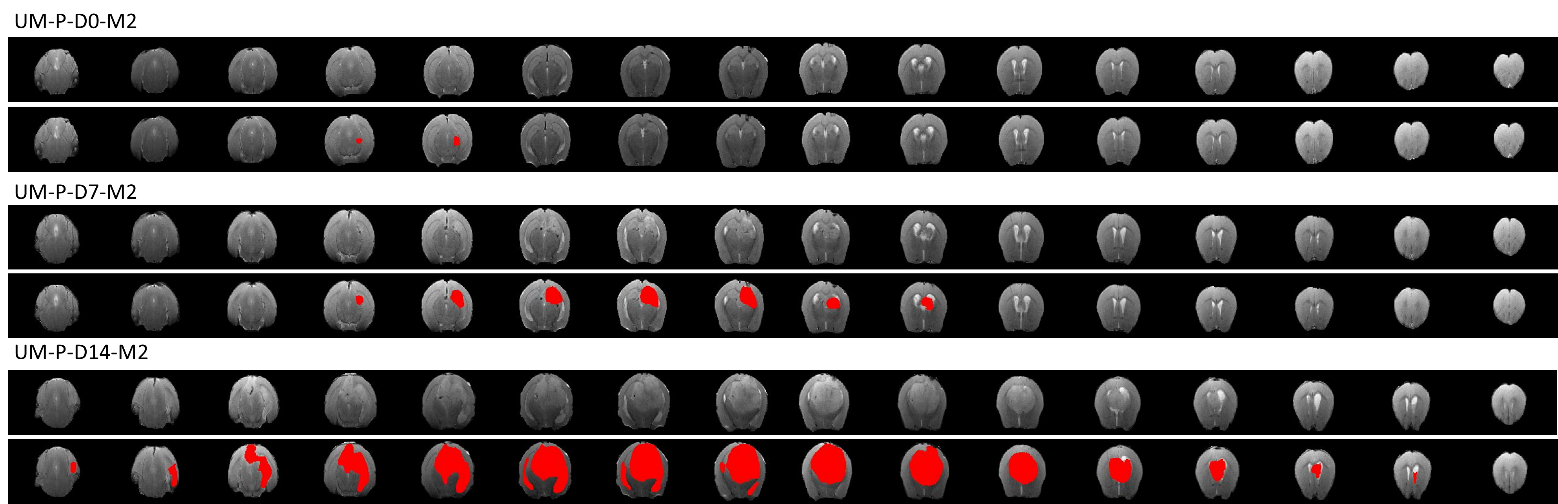


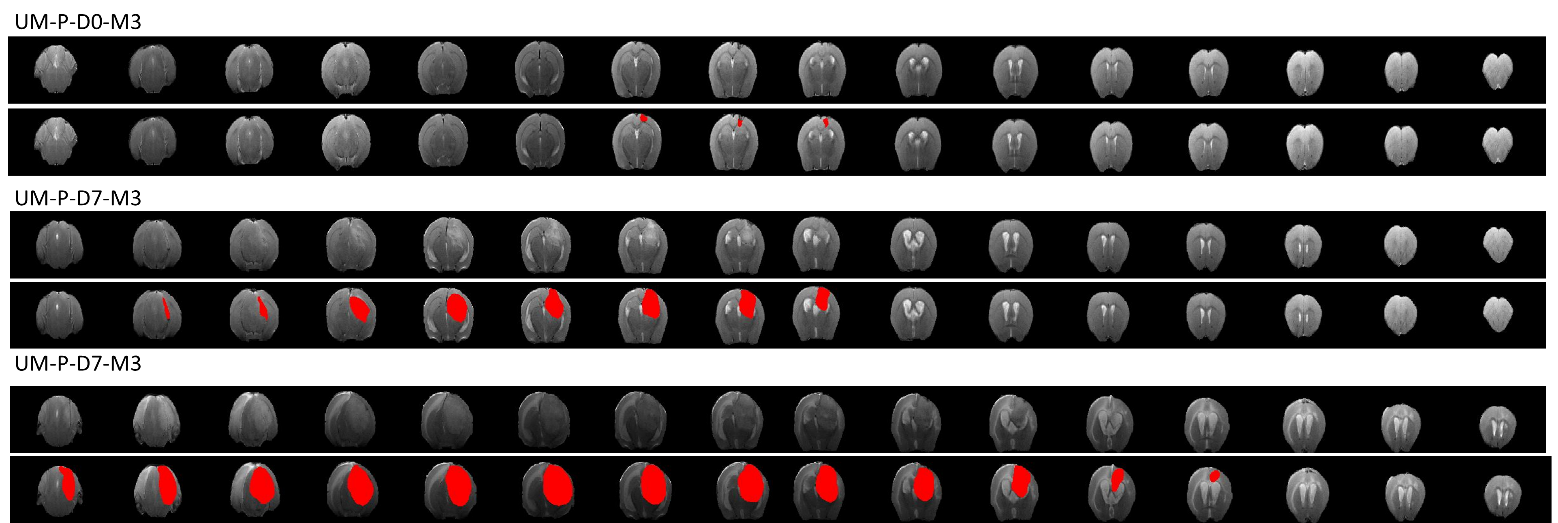


**MRI data of each UM-P group of mice (M2, M3, M16, M18, M20)**


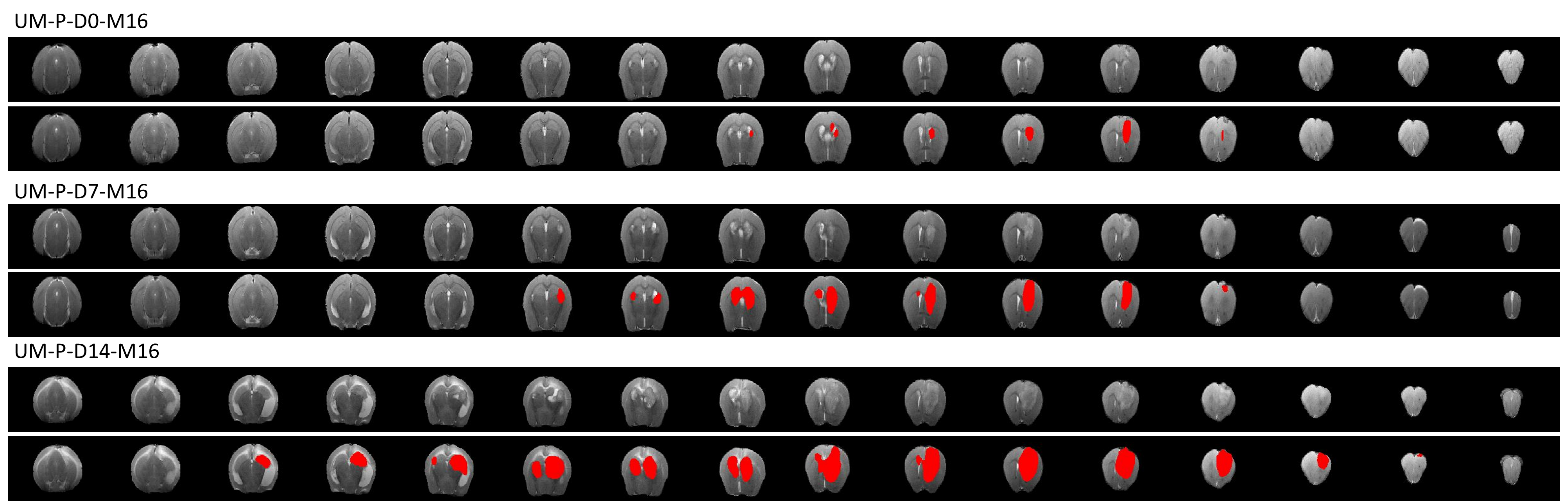


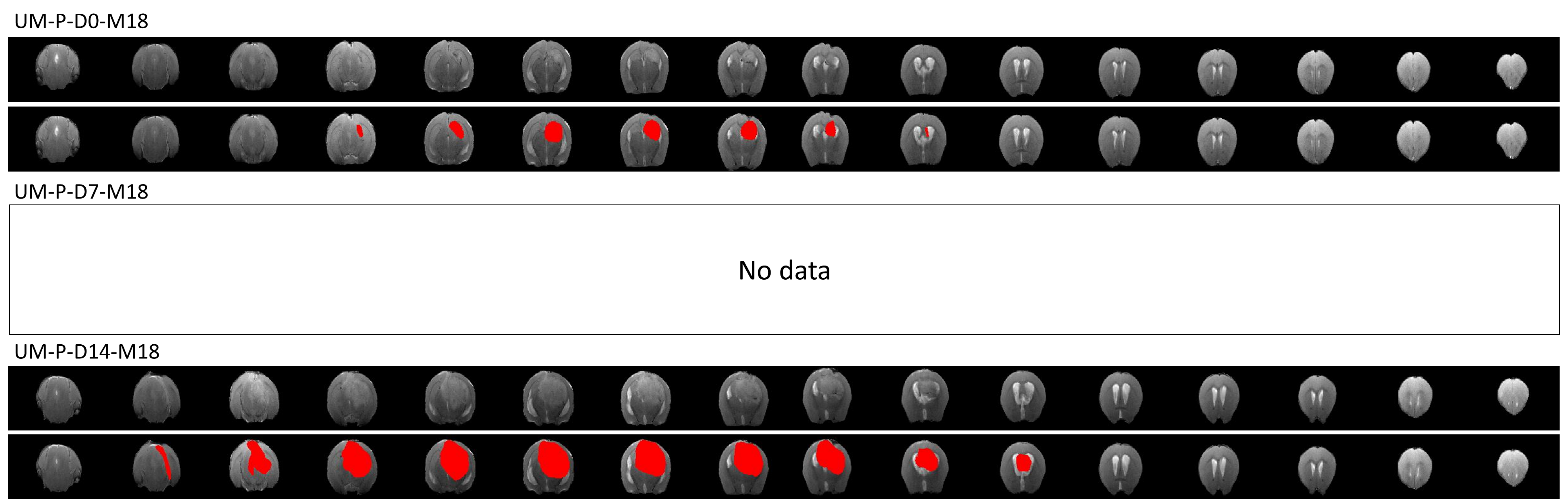


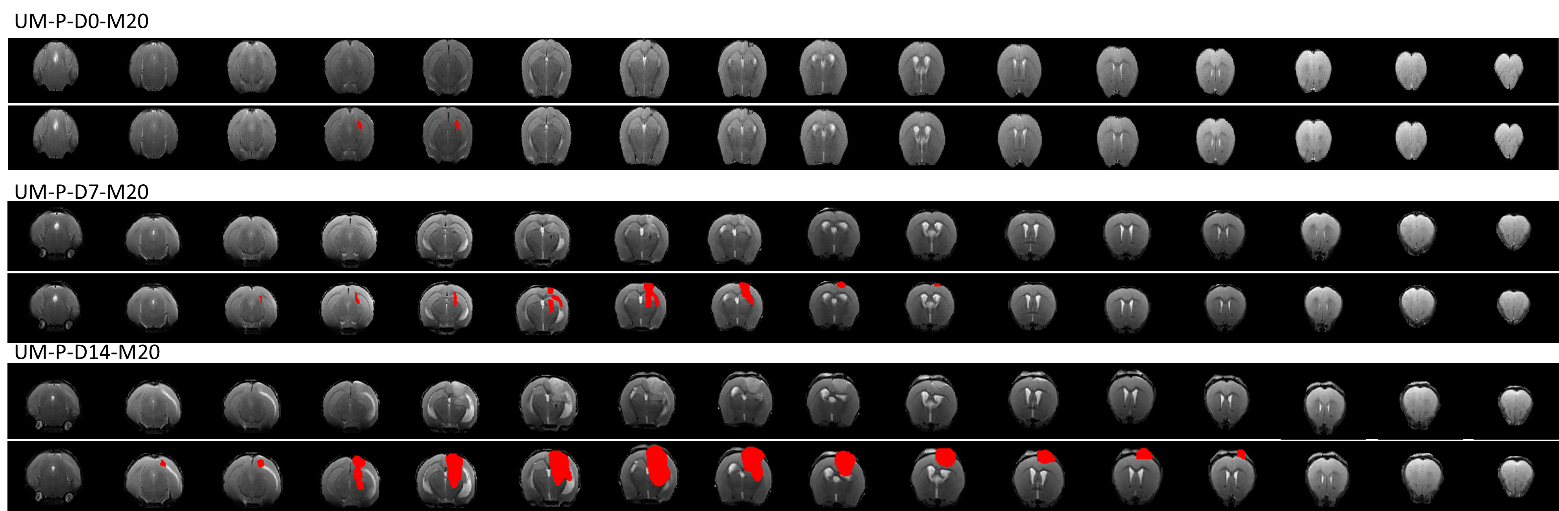


**MRI data of each UM-miR124-P group of mice (M17, M21, M23, M24, M27, M28)**


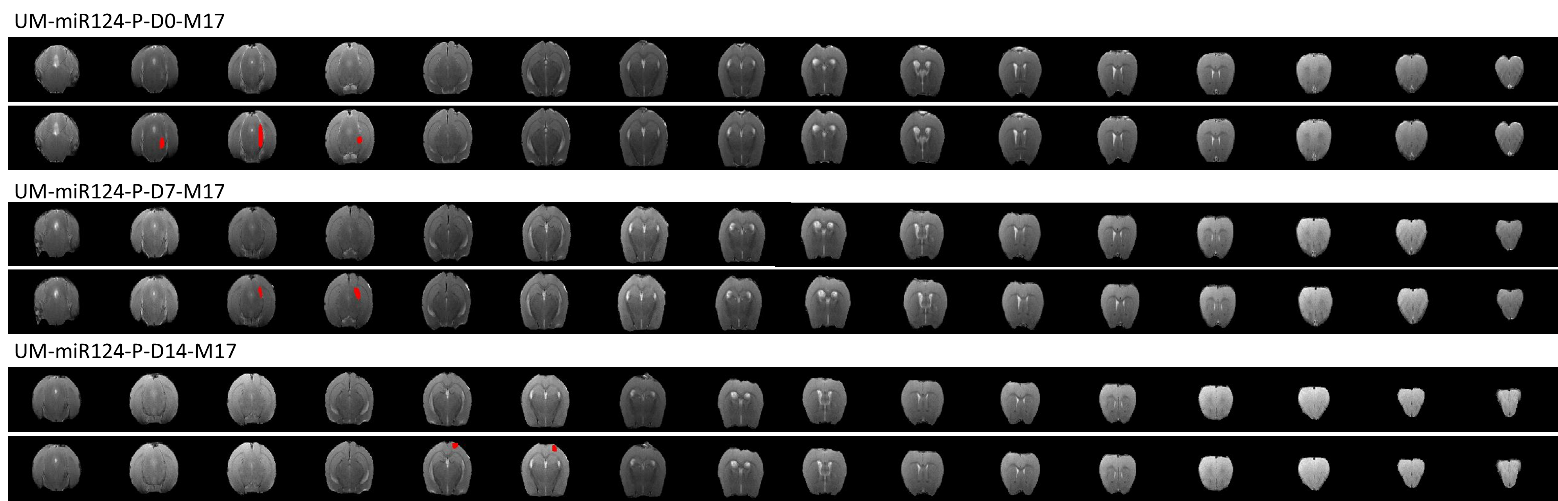


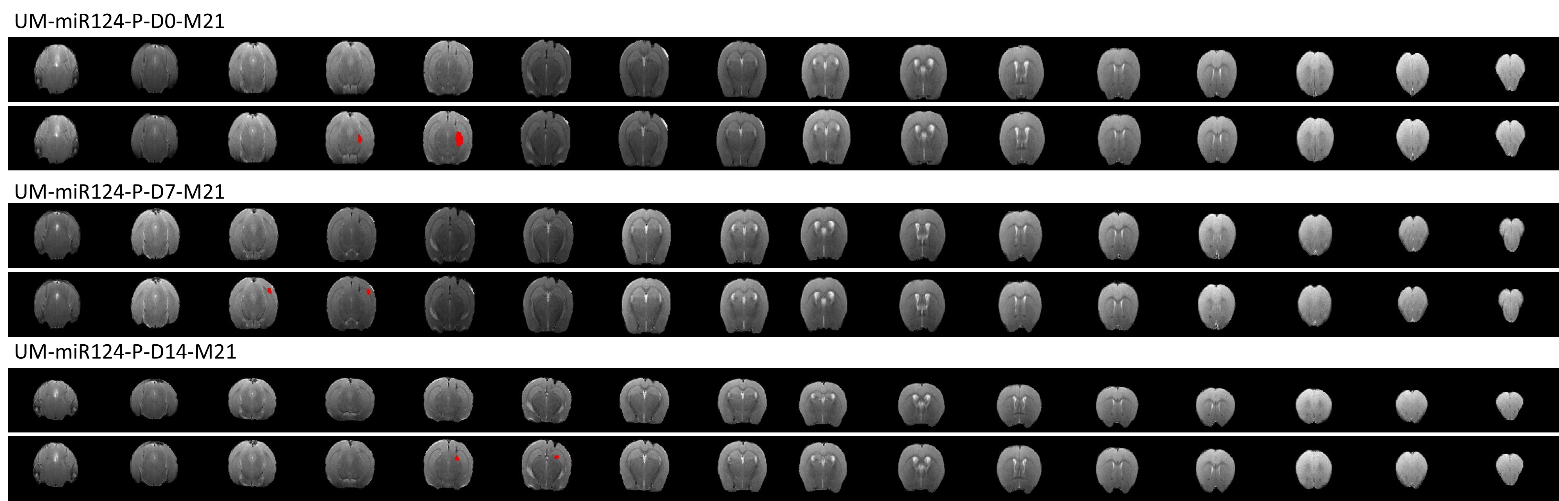


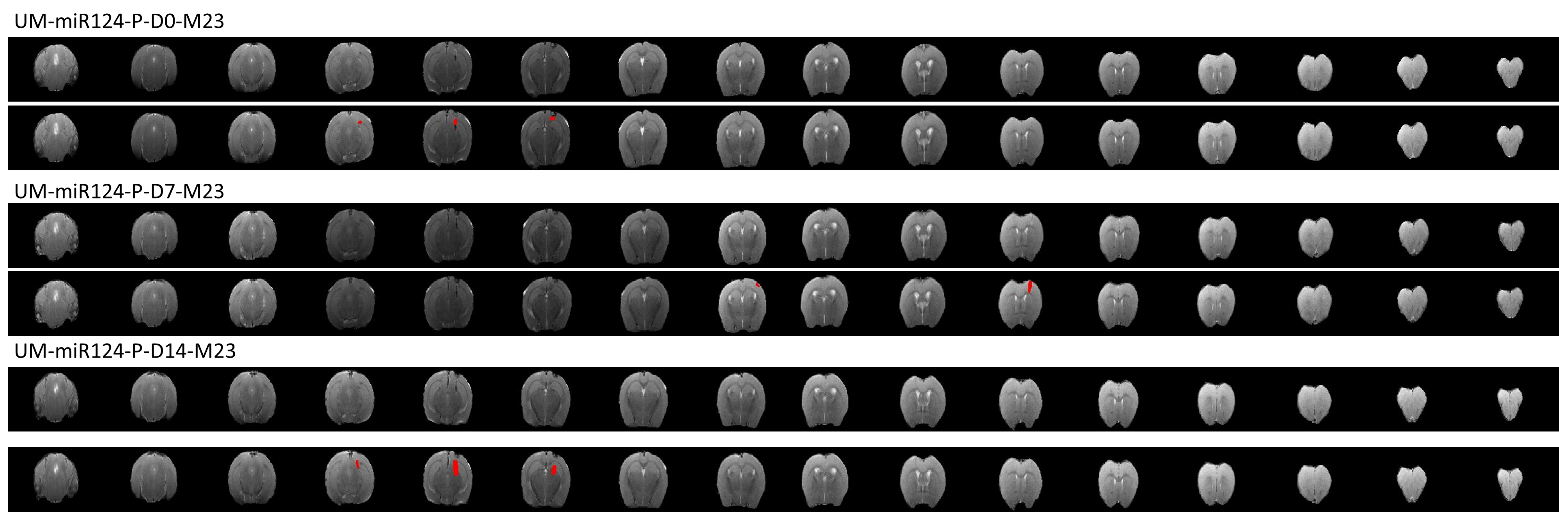


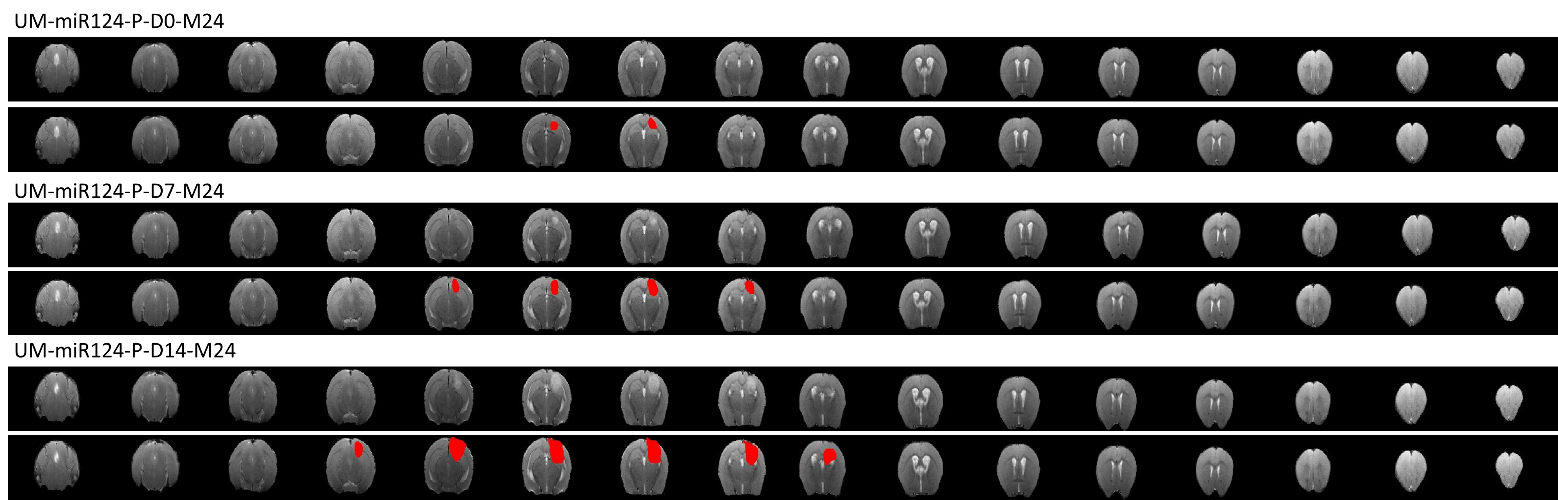


**MRI data of each UM-miR124-P group of mice (M17, M21, M23, M24, M27, M28)**


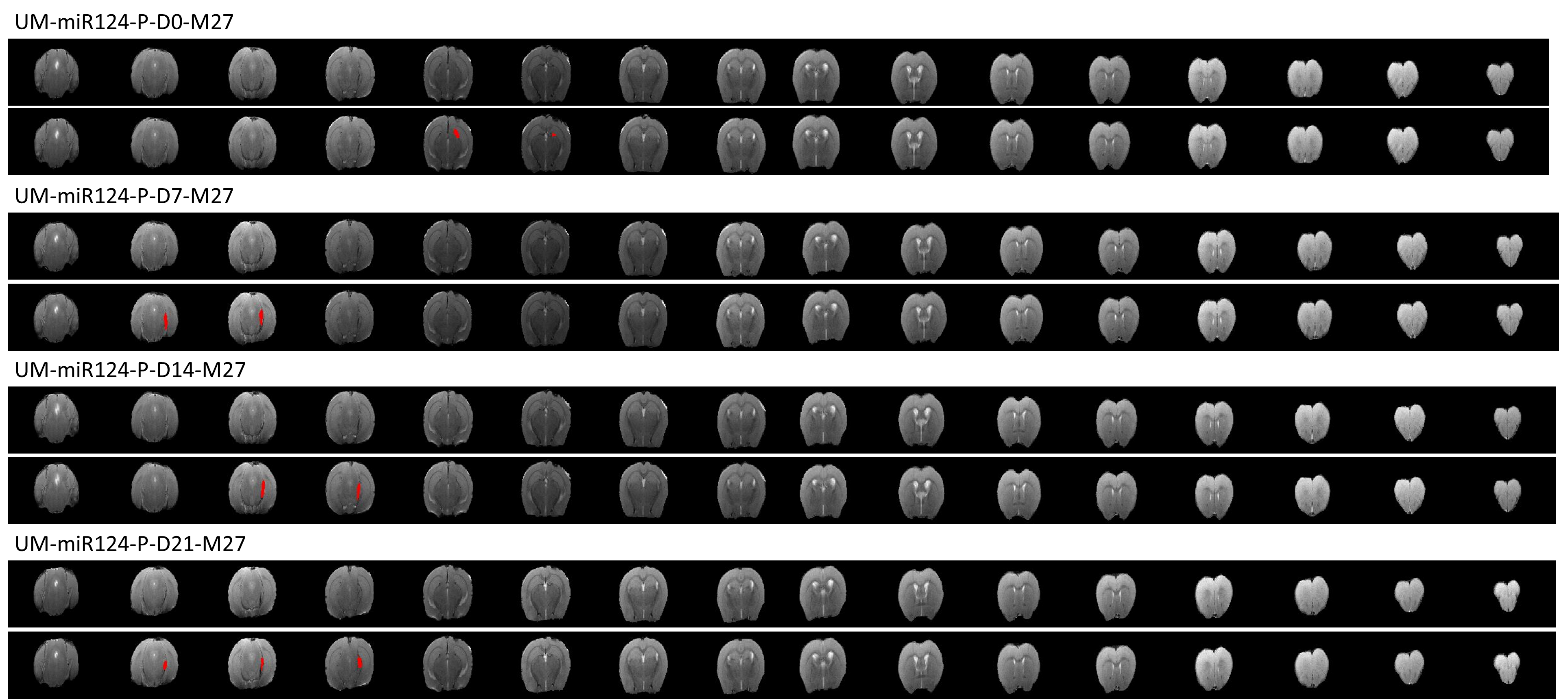


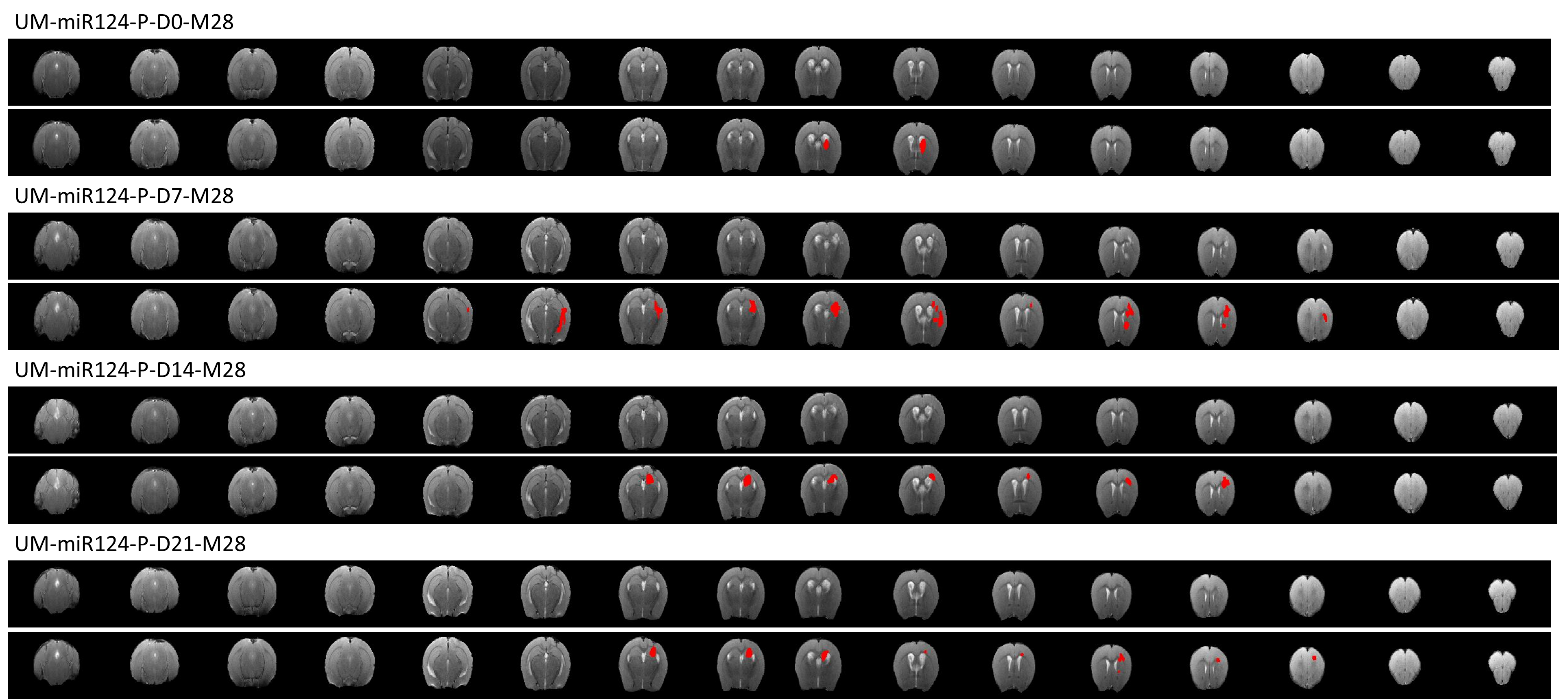


**MRI data of each UM-miR124-P EV group of mice (M19, M22, M25, M26, M29, M30)**


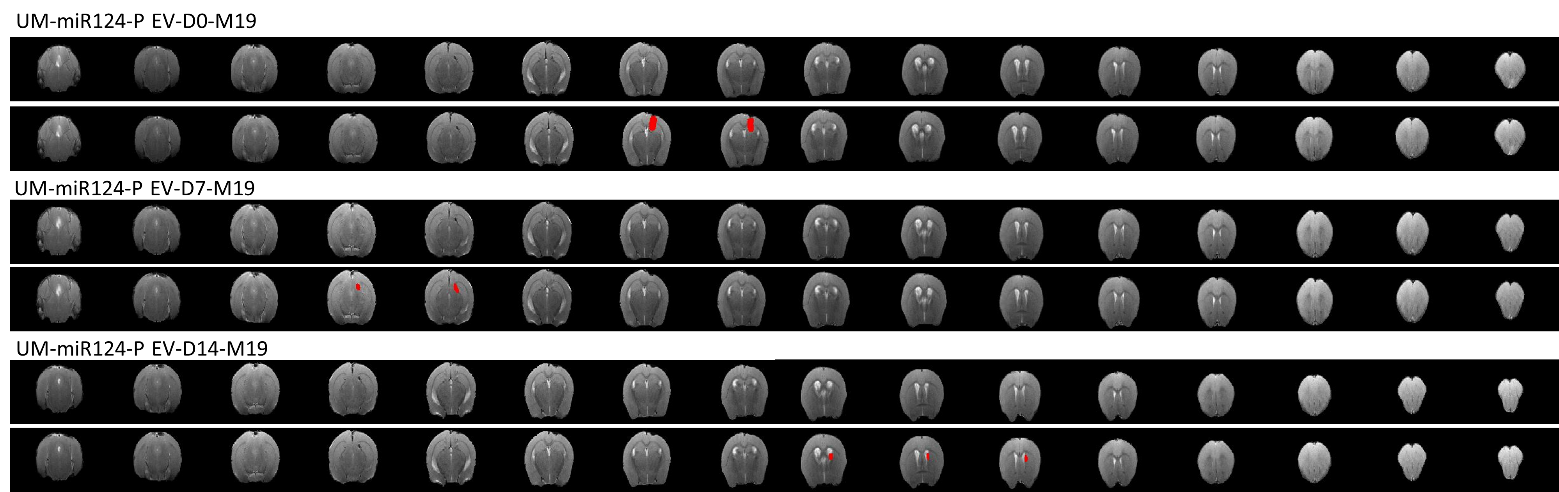


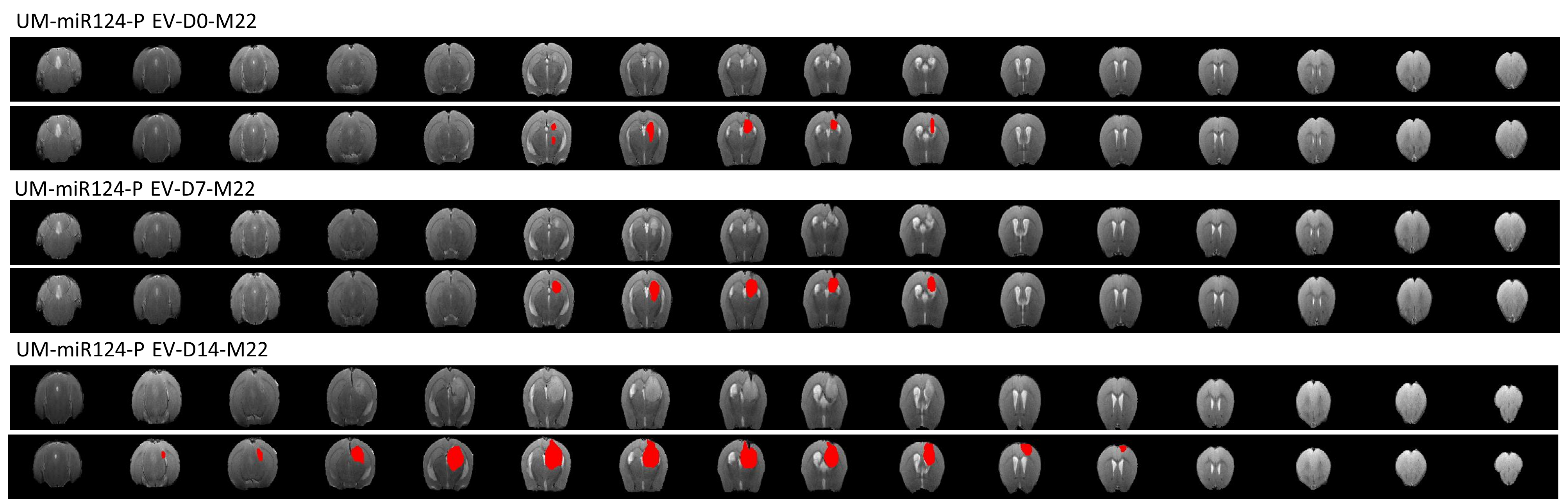


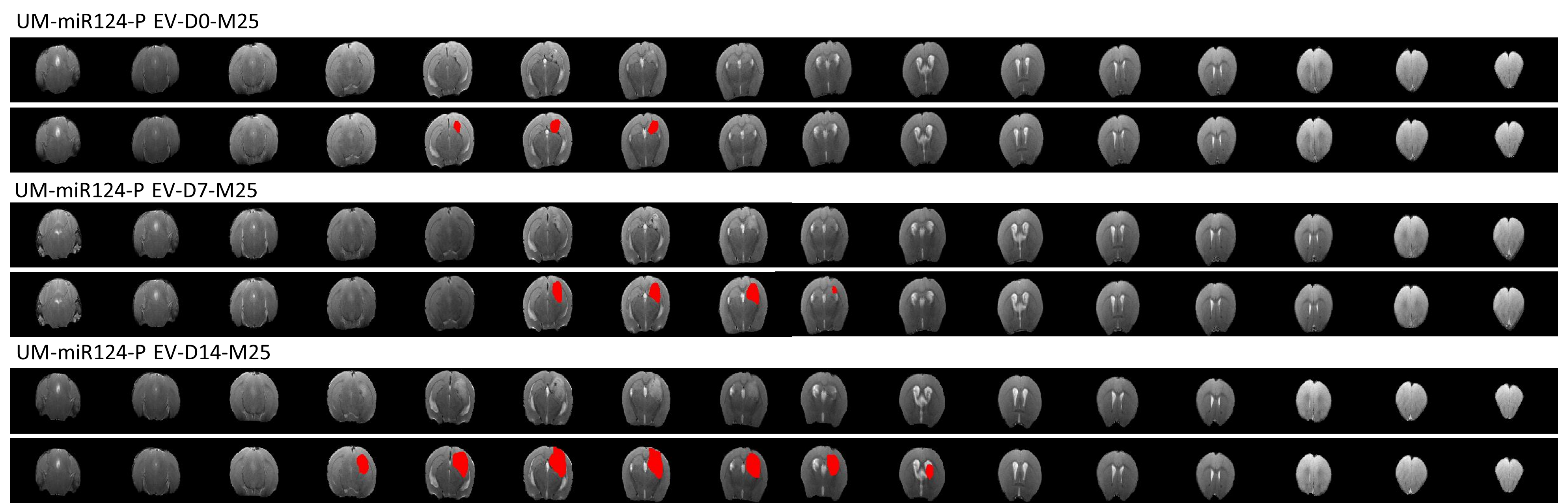


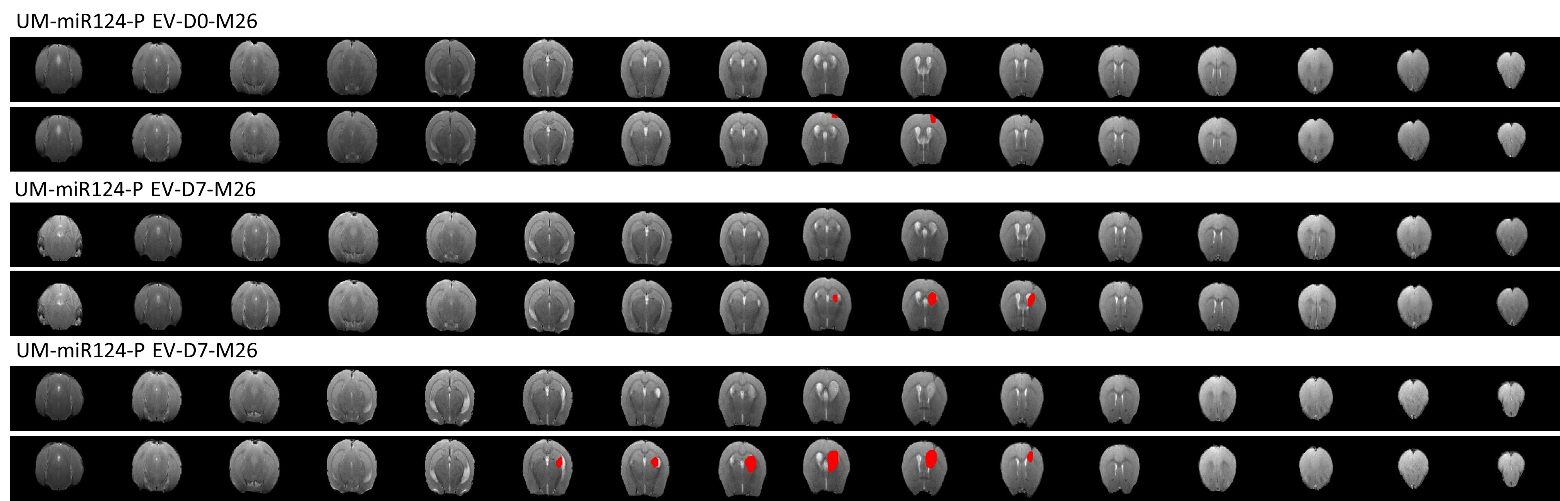


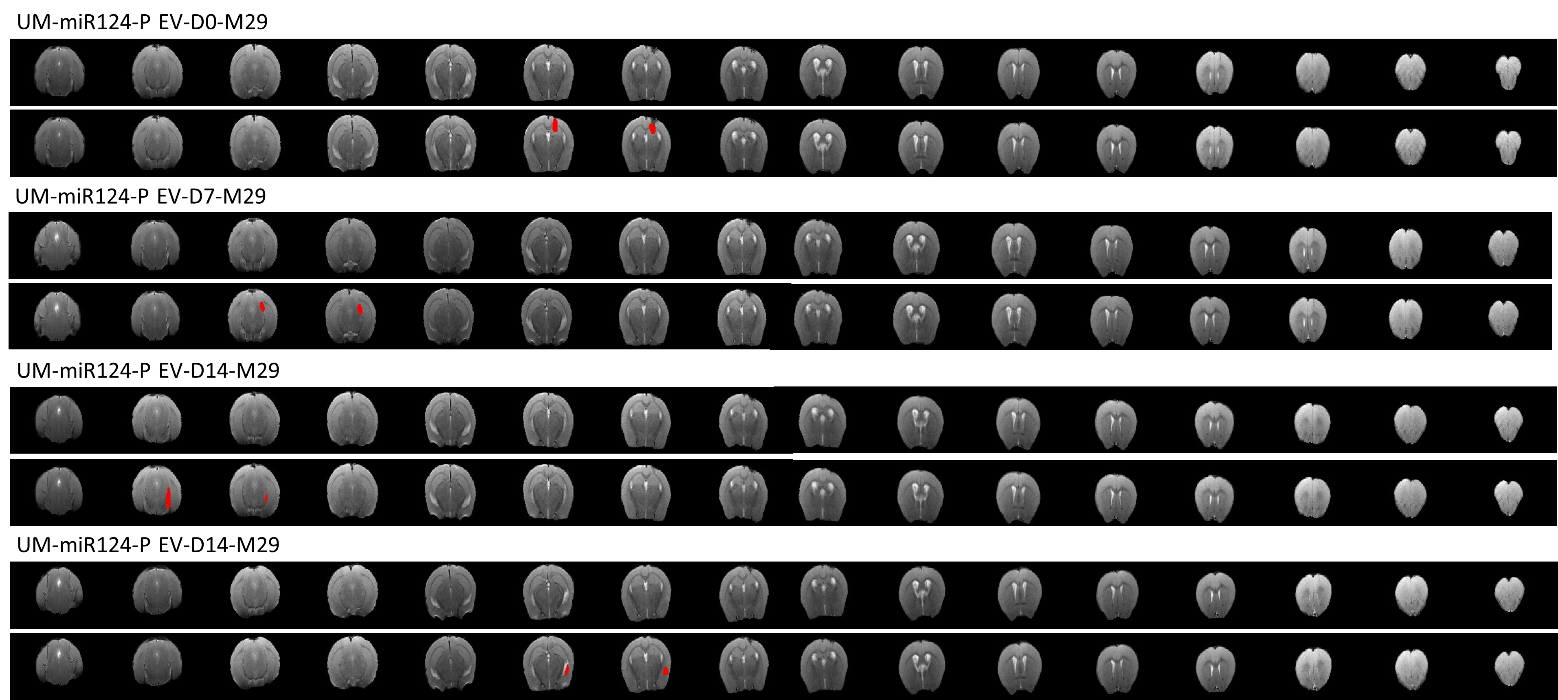


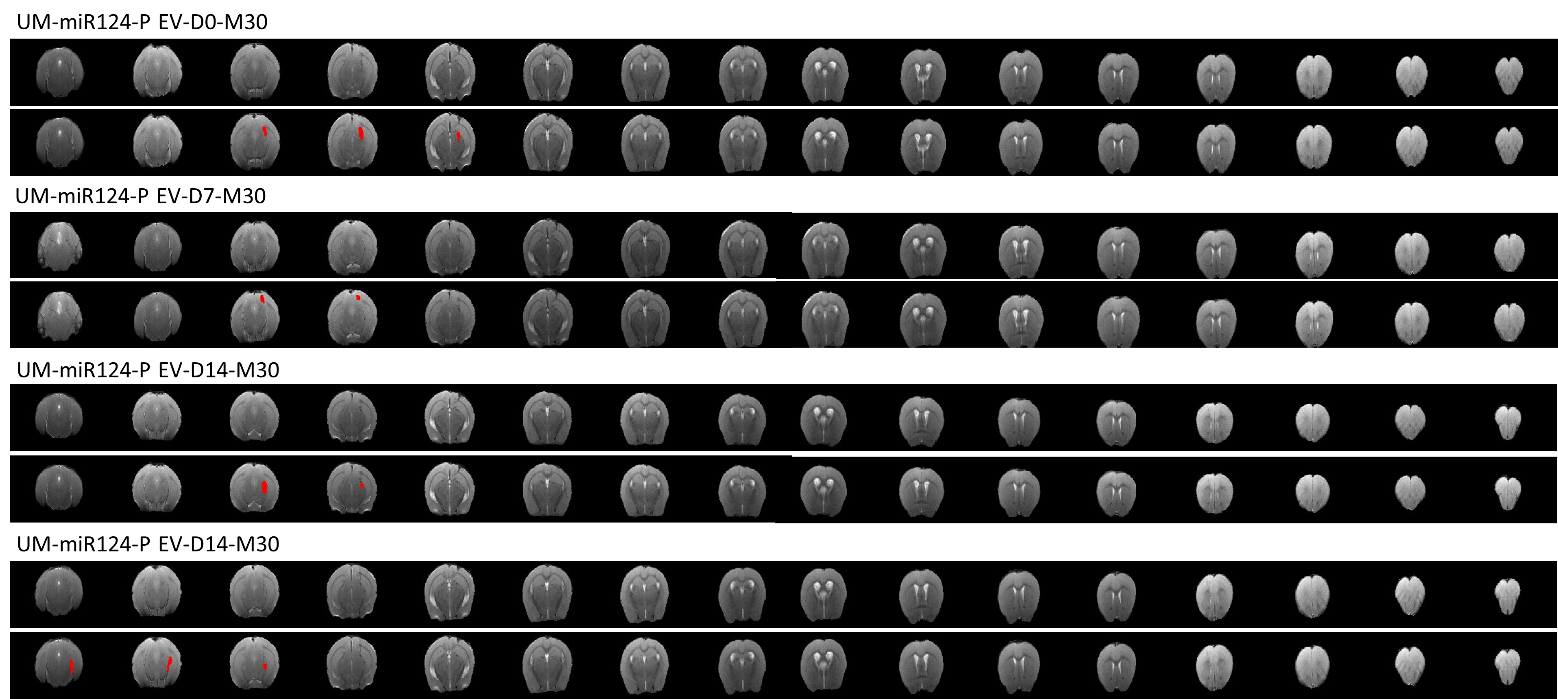

Supplement: Supplementary file 1 — Supplementary Material 1 [file 13046_2025_3336_MOESM1_ESM.docx]
